# Supplementary material for: Rationally derived inhibitors of hepatitis C virus (HCV) p7 channel activity reveal prospect for bimodal antiviral therapy
Source: eLife. 2020 Nov 10;9:e52555. doi: 10.7554/eLife.52555 (PMC7714397; doi:10.7554/eLife.52555)
Supplement: Figure 1—source data 1. [file elife-52555-fig1-data1.zip › SD-figure1/Exp019-JK332-geno/Activity of JK3-32 against gt3a HCV.docx]

## Activity of JK3-32 lead vs gt3a p7

Fresh 2mg stock of JK3-32 tested against S52-JFH1 (gt3a p7) in duplicate. Control IC_50_ curve was performed against J4-JFH1 (gt1b p7) (previous determined IC_50_ for gt1b (with increasing compound freeze-thaw) = 175.5 nM, 60.8 nM, 447.1 nM, 610.5 nM).

**Control J4-JFH1 JK3-32 IC_50_**

**IC_50_ = 242 nM**

**The same JK3-32 sample tested against S52-JFH1.**

**IC_50_ = 641.9 nM**

**IC_50_ = 834.4 nM**

**Daclatasvir control –** should show similar sensitivity targeting JFH1 derived NS5A from both chimeric viruses.

**IC_50_ = 0.8 pM**

**IC_50_ = 2.5 pM**

Additional molecules within SAR of JK3-32 (with significantly lower activity, which cannot always fit exact IC_50_) also show reduced activity against gt3a vs gt1b.

However, despite possible differences in sensitivity between gt1b and gt3a, JK3-32 clearly shows reasonable nM activity against both genotypes.
