# Supplementary material for: Rationally derived inhibitors of hepatitis C virus (HCV) p7 channel activity reveal prospect for bimodal antiviral therapy
Source: eLife. 2020 Nov 10;9:e52555. doi: 10.7554/eLife.52555 (PMC7714397; doi:10.7554/eLife.52555)
Supplement: Figure 1—figure supplement 1—source data 1. [file elife-52555-fig1-figsupp1-data1.zip › SD-figureS1/Exp002-assay/Figure/Figure.pptx]

## Slide 1
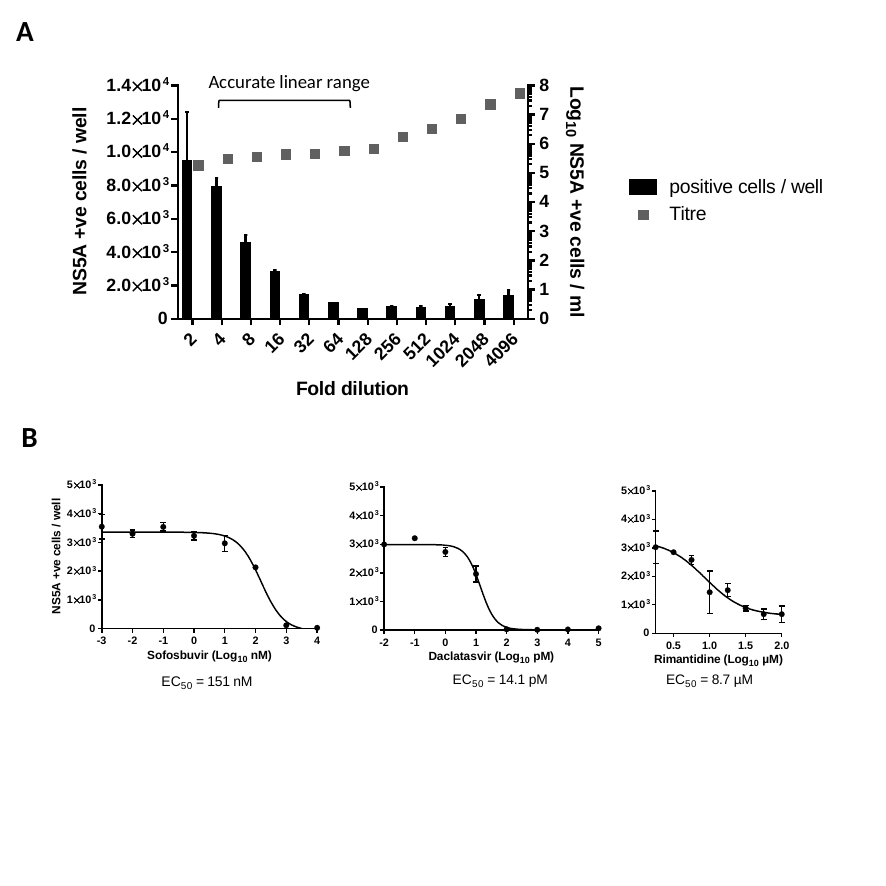

A
Accurate linear range
B

## Slide 2
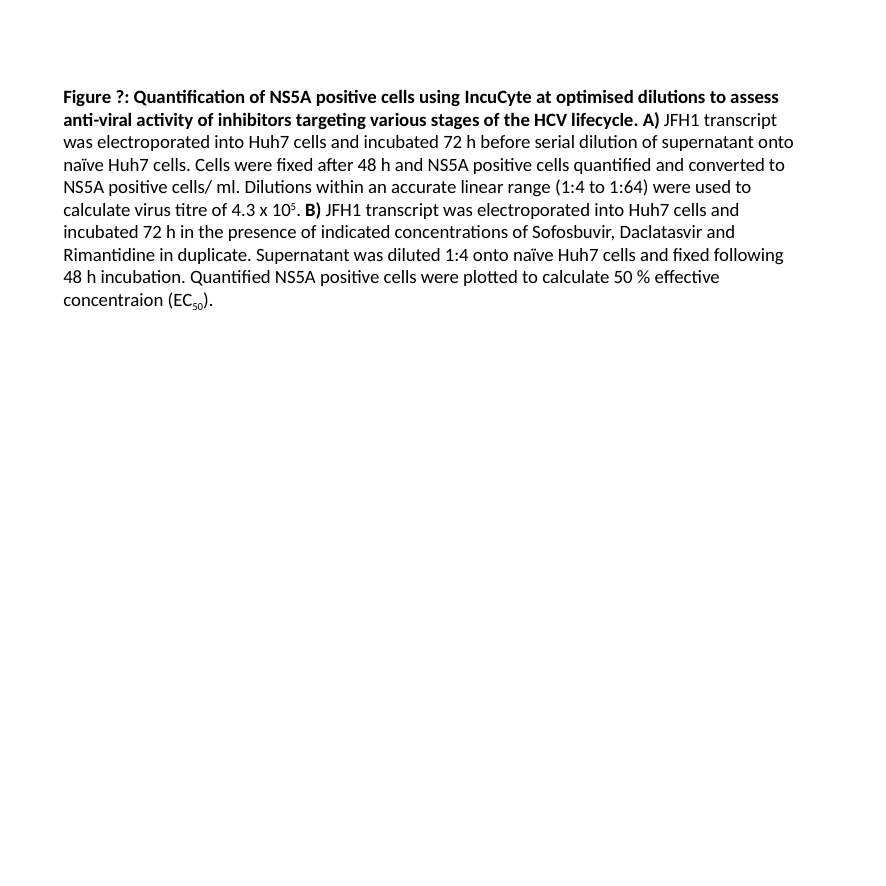

# Figure ?: Quantification of NS5A positive cells using IncuCyte at optimised dilutions to assess anti-viral activity of inhibitors targeting various stages of the HCV lifecycle. A) JFH1 transcript was electroporated into Huh7 cells and incubated 72 h before serial dilution of supernatant onto naïve Huh7 cells. Cells were fixed after 48 h and NS5A positive cells quantified and converted to NS5A positive cells/ ml. Dilutions within an accurate linear range (1:4 to 1:64) were used to calculate virus titre of 4.3 x 10^5^. B) JFH1 transcript was electroporated into Huh7 cells and incubated 72 h in the presence of indicated concentrations of Sofosbuvir, Daclatasvir and Rimantidine in duplicate. Supernatant was diluted 1:4 onto naïve Huh7 cells and fixed following 48 h incubation. Quantified NS5A positive cells were plotted to calculate 50 % effective concentraion (EC50).
