# Supplementary material for: Rationally derived inhibitors of hepatitis C virus (HCV) p7 channel activity reveal prospect for bimodal antiviral therapy
Source: eLife. 2020 Nov 10;9:e52555. doi: 10.7554/eLife.52555 (PMC7714397; doi:10.7554/eLife.52555)
Supplement: Figure 1—figure supplement 1—source data 1. [file elife-52555-fig1-figsupp1-data1.zip › SD-figureS1/Exp002-assay/Figure/Figure_old.pptx]

## Slide 1
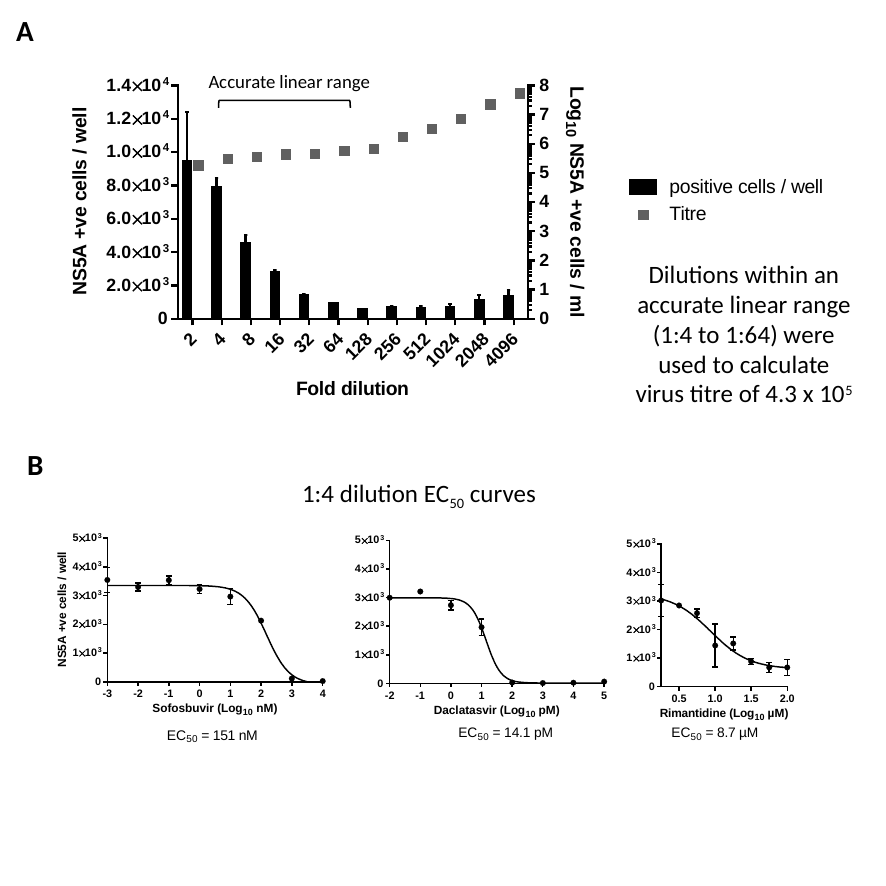

A
Accurate linear range
Dilutions within an accurate linear range (1:4 to 1:64) were used to calculate virus titre of 4.3 x 10^5^
B
1:4 dilution EC50 curves
