# Supplementary figures and images for: Rationally derived inhibitors of hepatitis C virus (HCV) p7 channel activity reveal prospect for bimodal antiviral therapy
Source: eLife. 2020 Nov 10;9:e52555. doi: 10.7554/eLife.52555 (PMC7714397; doi:10.7554/eLife.52555)

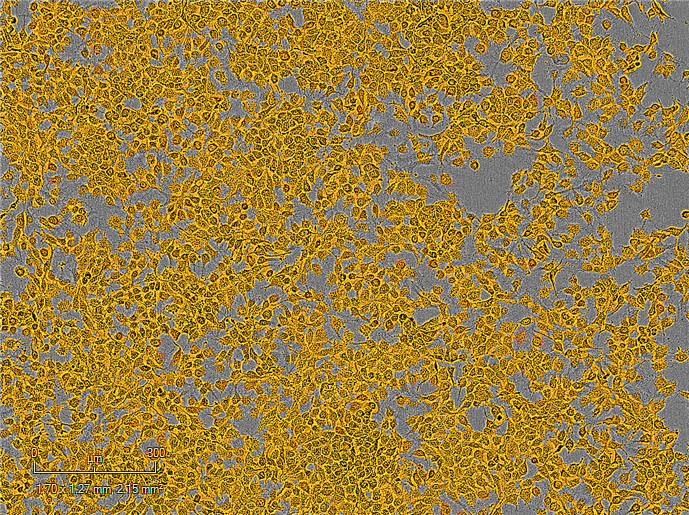

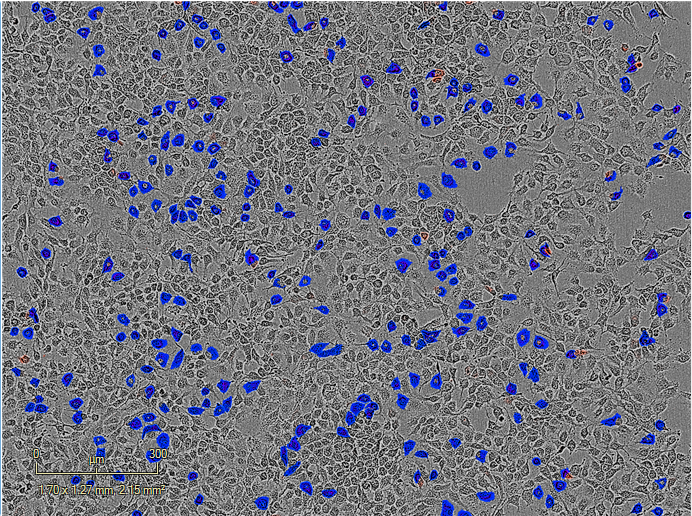

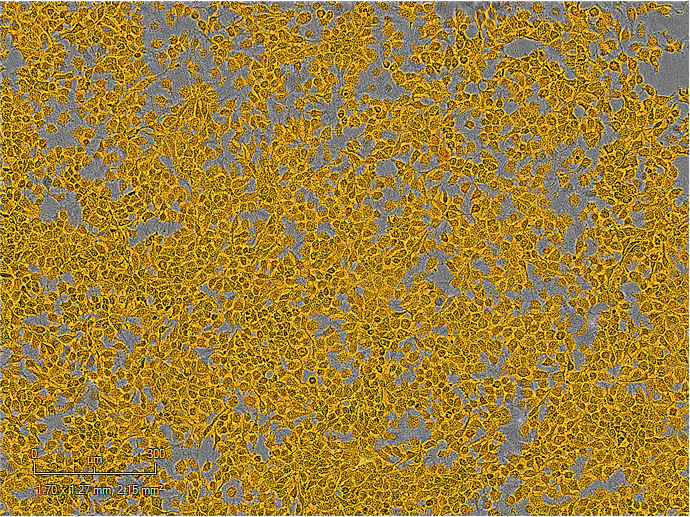

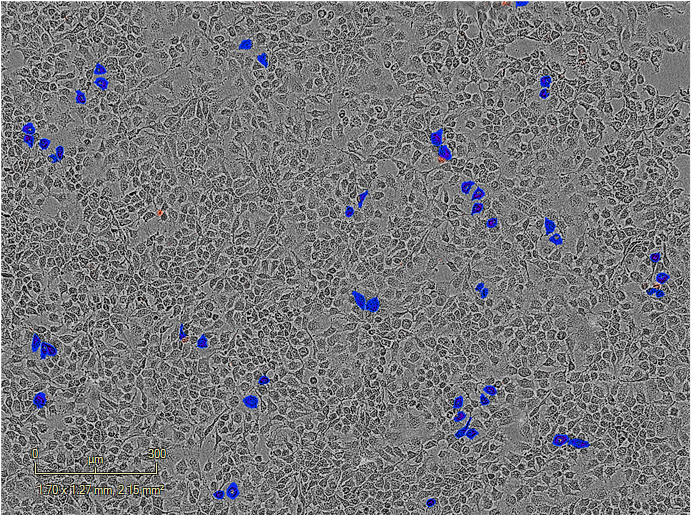

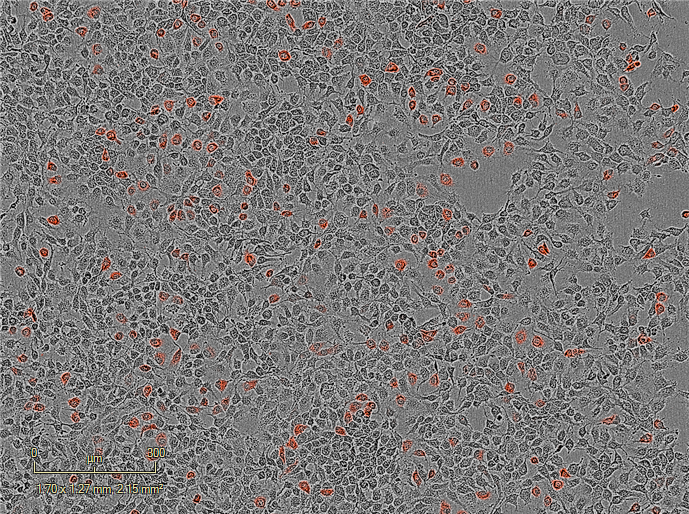

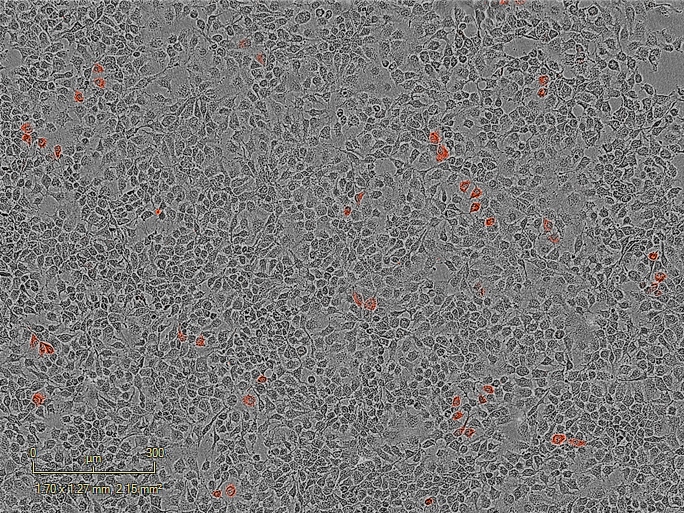


Red cells/ well

**31.6 µM RIM**

**DMSO**

[RIM] µM

100

56.2

31.6

17.8

10

5.6

3.16

0

Supplement: Figure 1—figure supplement 1—source data 1. [file elife-52555-fig1-figsupp1-data1.zip › SD-figureS1/Exp002-assay/Example Incucyte data.docx]

## Slide 1
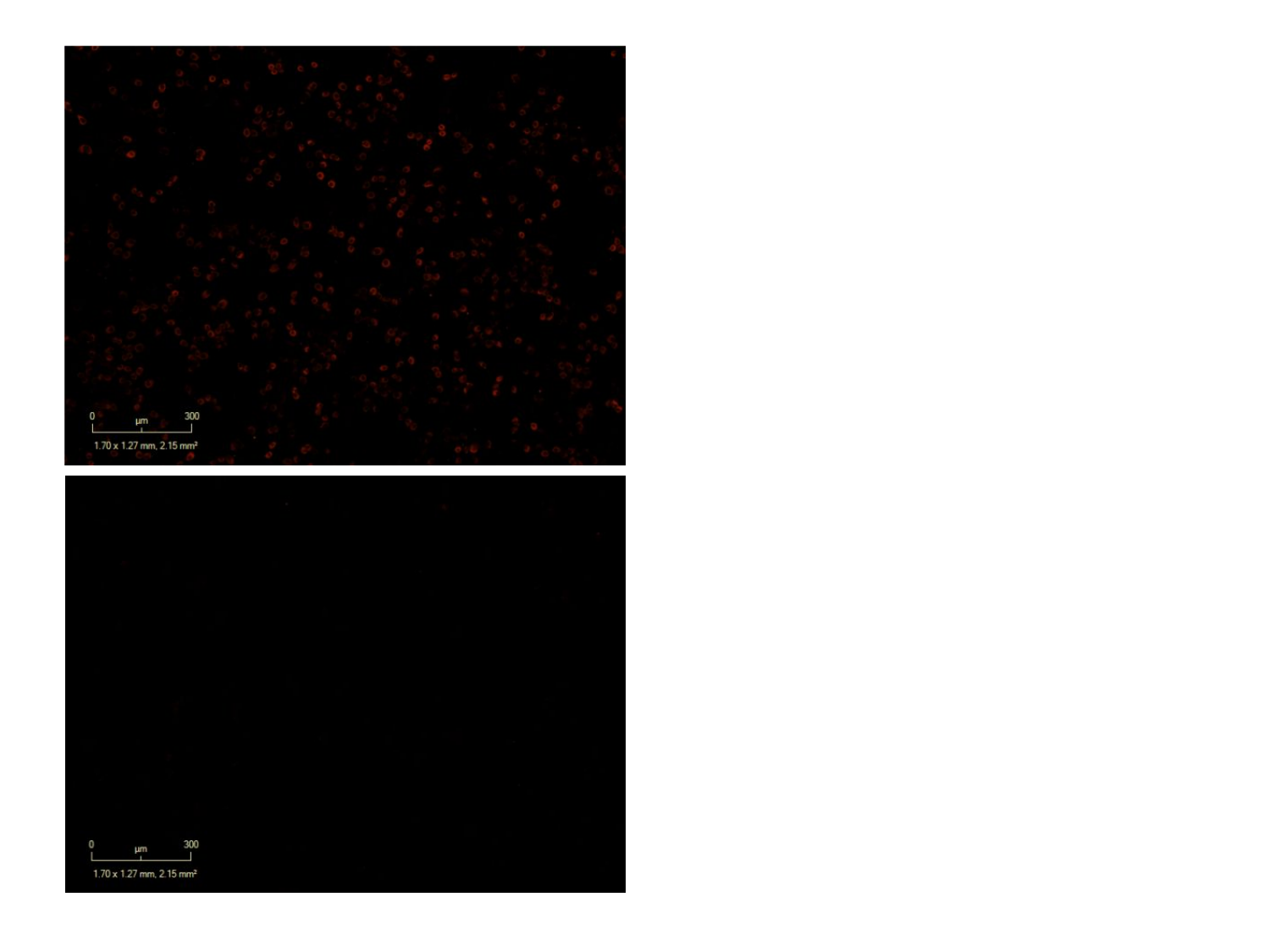

Supplement: Figure 1—figure supplement 1—source data 1. [file elife-52555-fig1-figsupp1-data1.zip › SD-figureS1/Exp005-assay/Huh7 1in4 JFH image.pptx]

## Slide 1
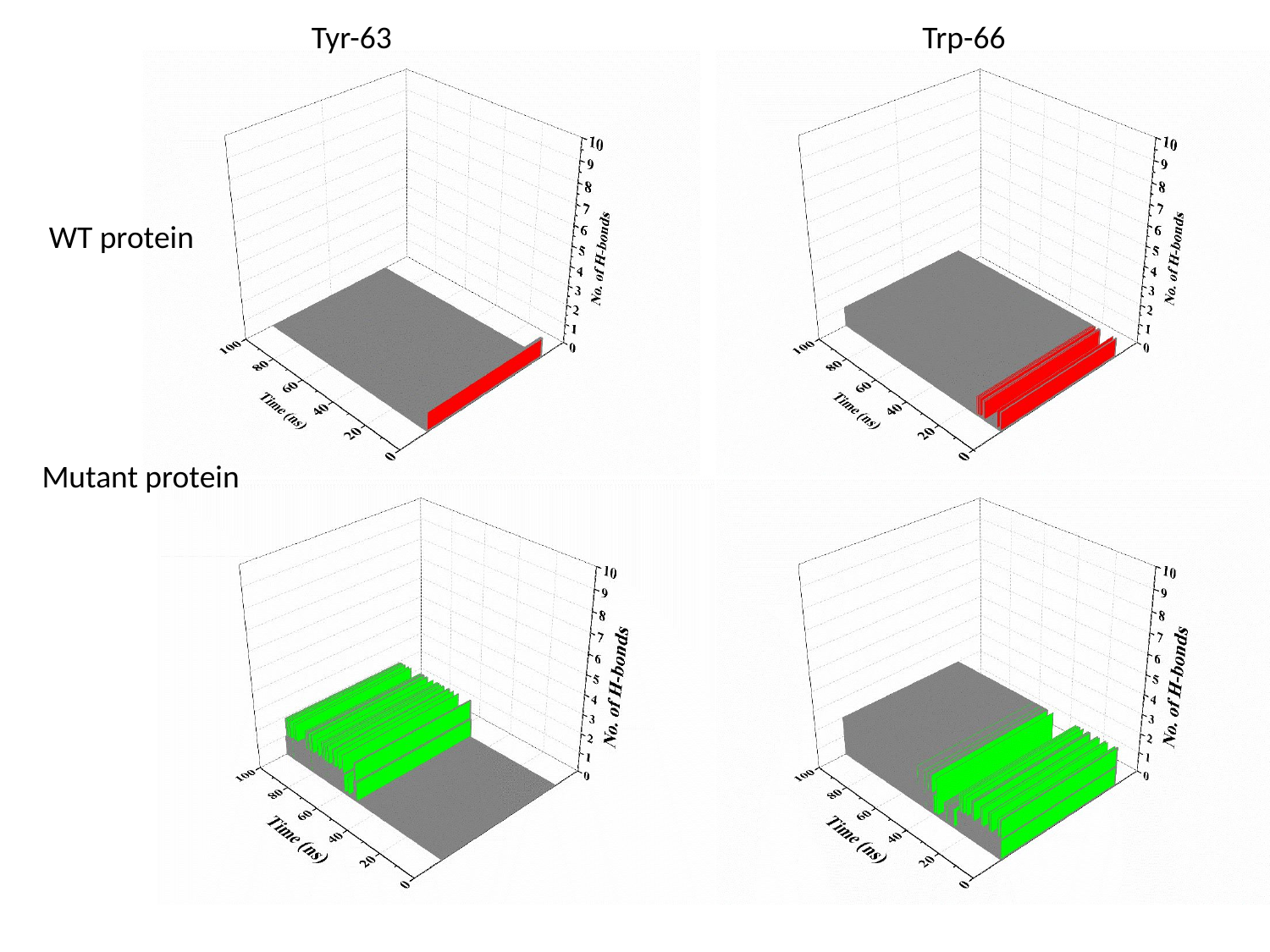

Tyr-63
Trp-66
WT protein
Mutant protein

Supplement: Figure 3—source data 1. [file elife-52555-fig3-data1.zip › SD-figure3/new run 2 files/H-Bonding.pptx]

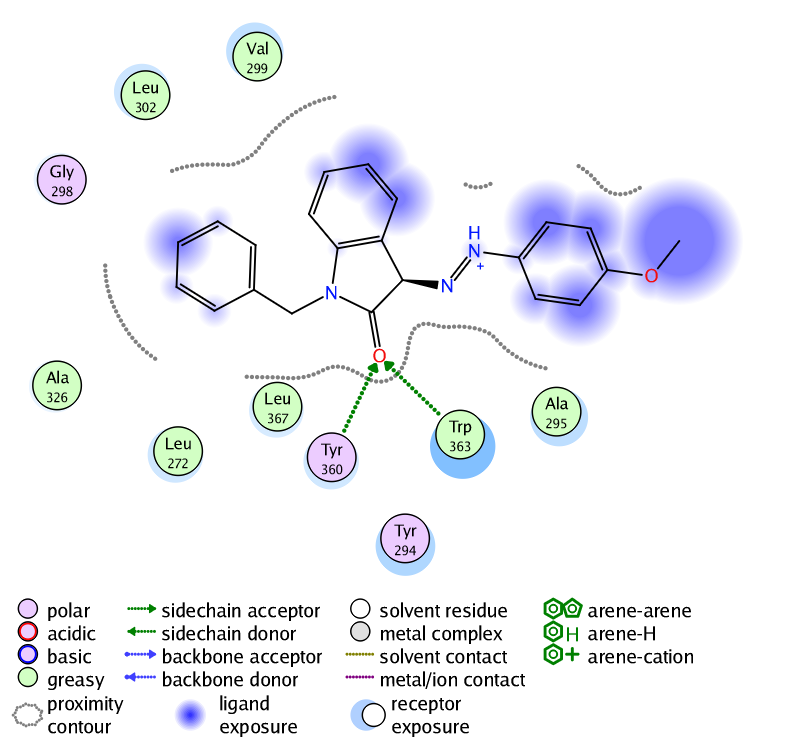

Supplement: Figure 3—source data 1. [file elife-52555-fig3-data1.zip › SD-figure3/new run 2 files/p7_0ns_contact-WT.png]

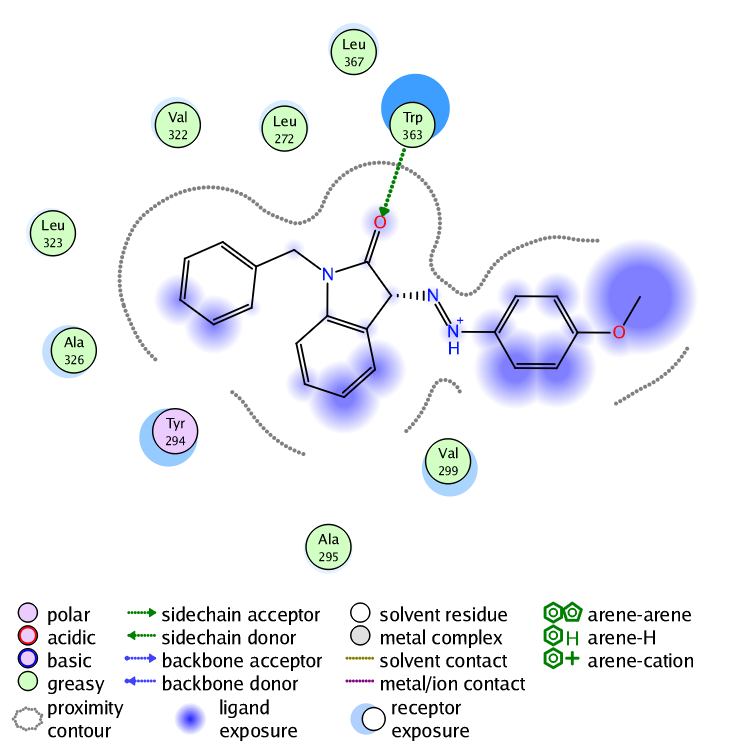

Supplement: Figure 3—source data 1. [file elife-52555-fig3-data1.zip › SD-figure3/new run 2 files/p7_100ns_contact-WT.png]

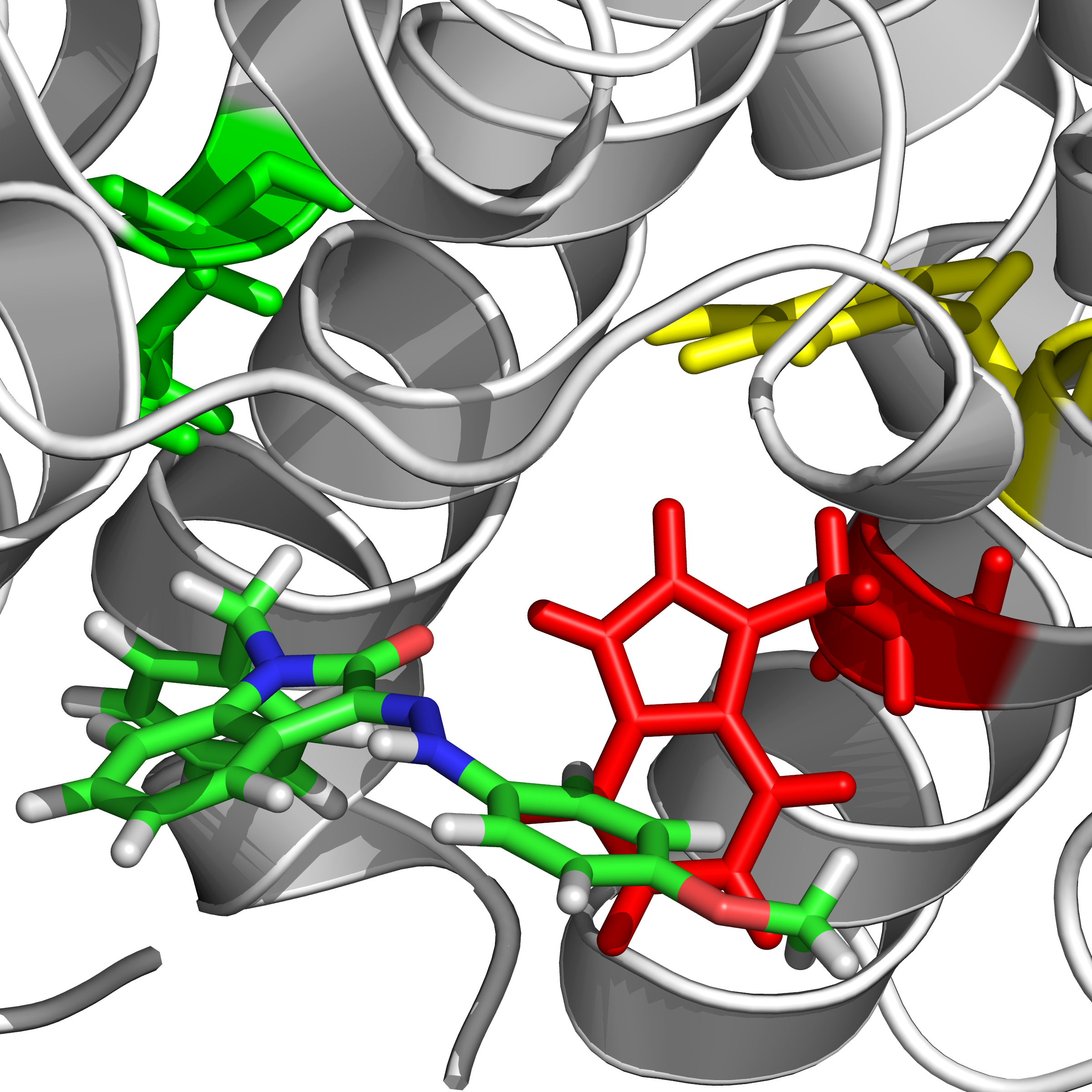

Supplement: Figure 3—source data 1. [file elife-52555-fig3-data1.zip › SD-figure3/new run 2 files/p7_937_wt2.png]

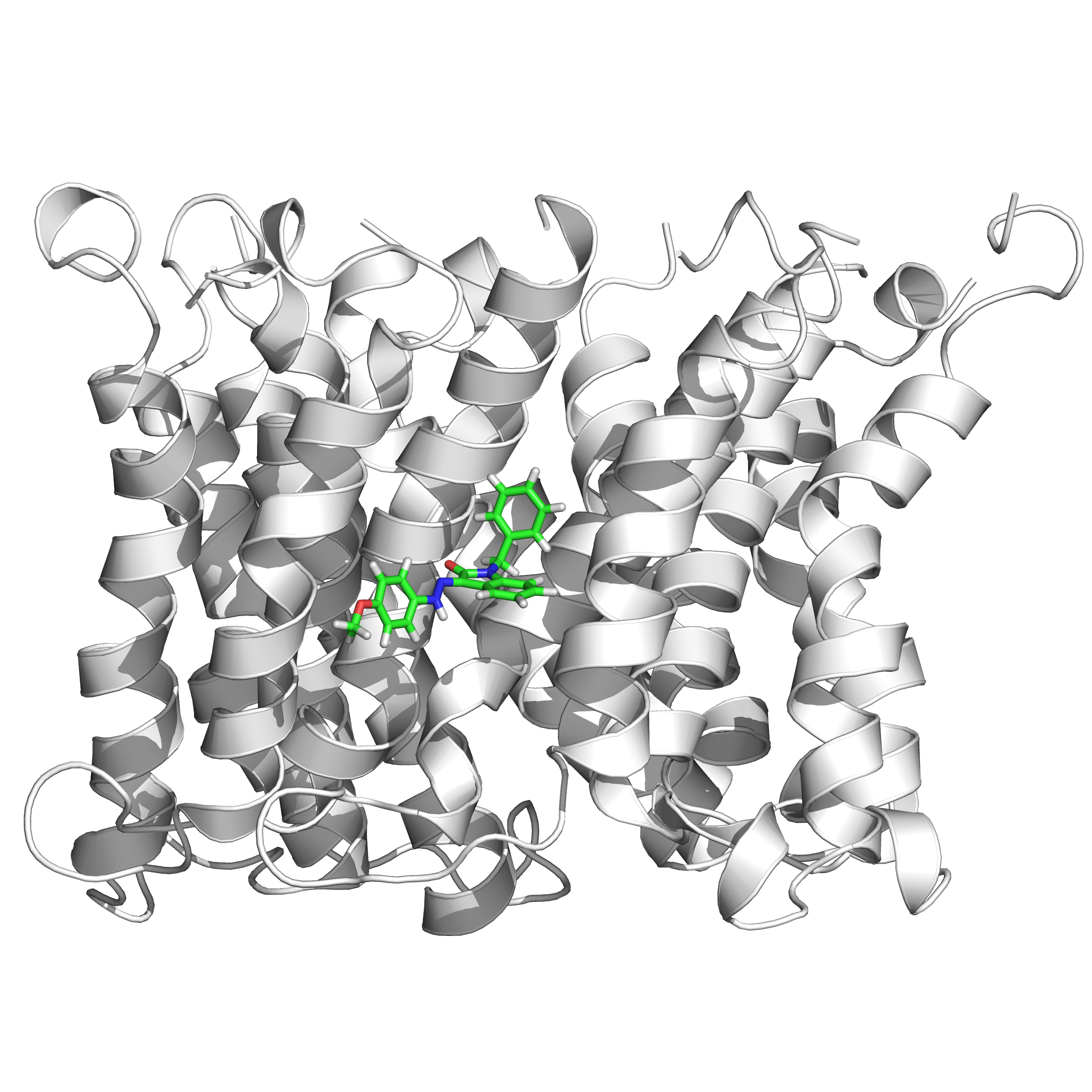

Supplement: Figure 3—source data 1. [file elife-52555-fig3-data1.zip › SD-figure3/new run files/0ns_side.png]

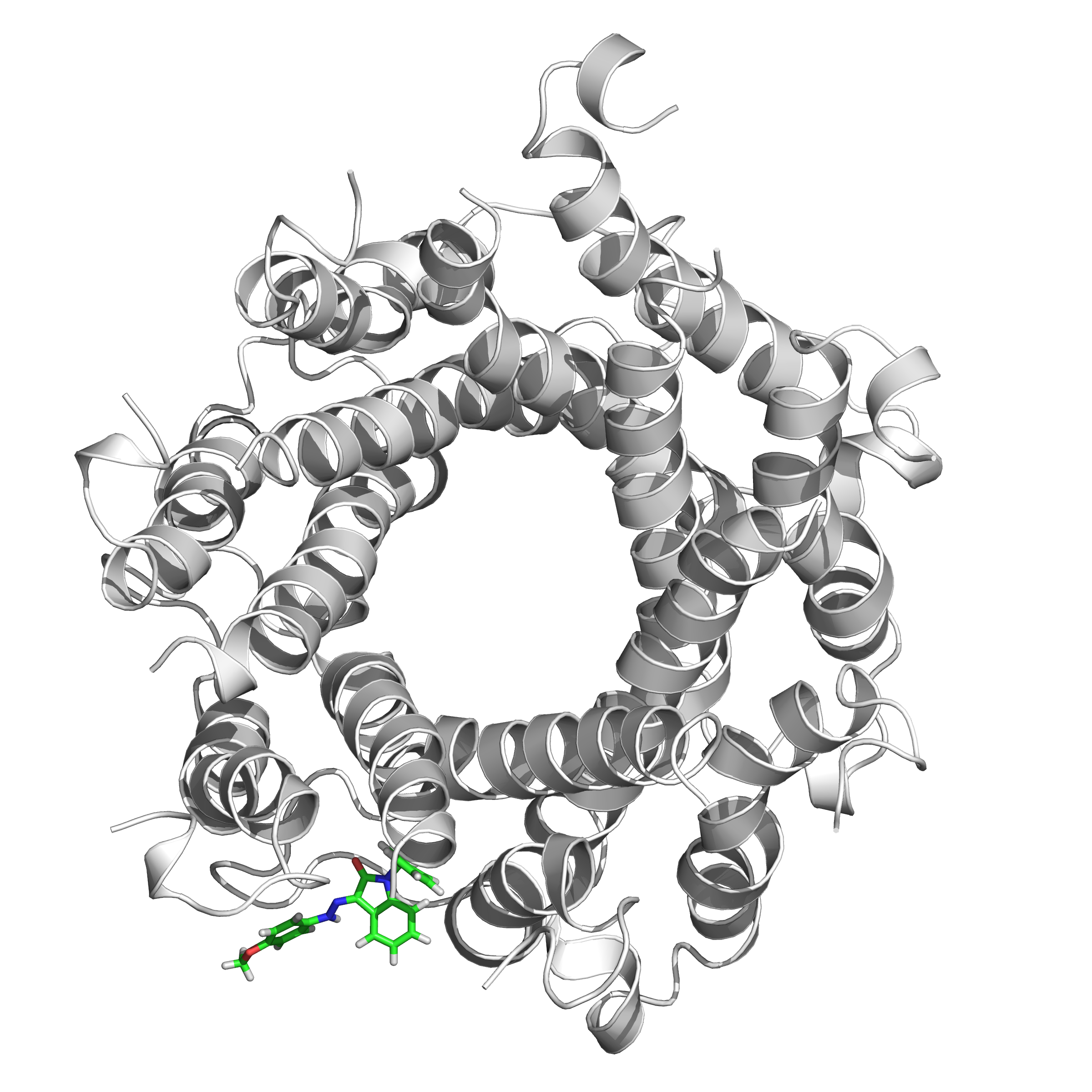

Supplement: Figure 3—source data 1. [file elife-52555-fig3-data1.zip › SD-figure3/new run files/0ns_top.png]

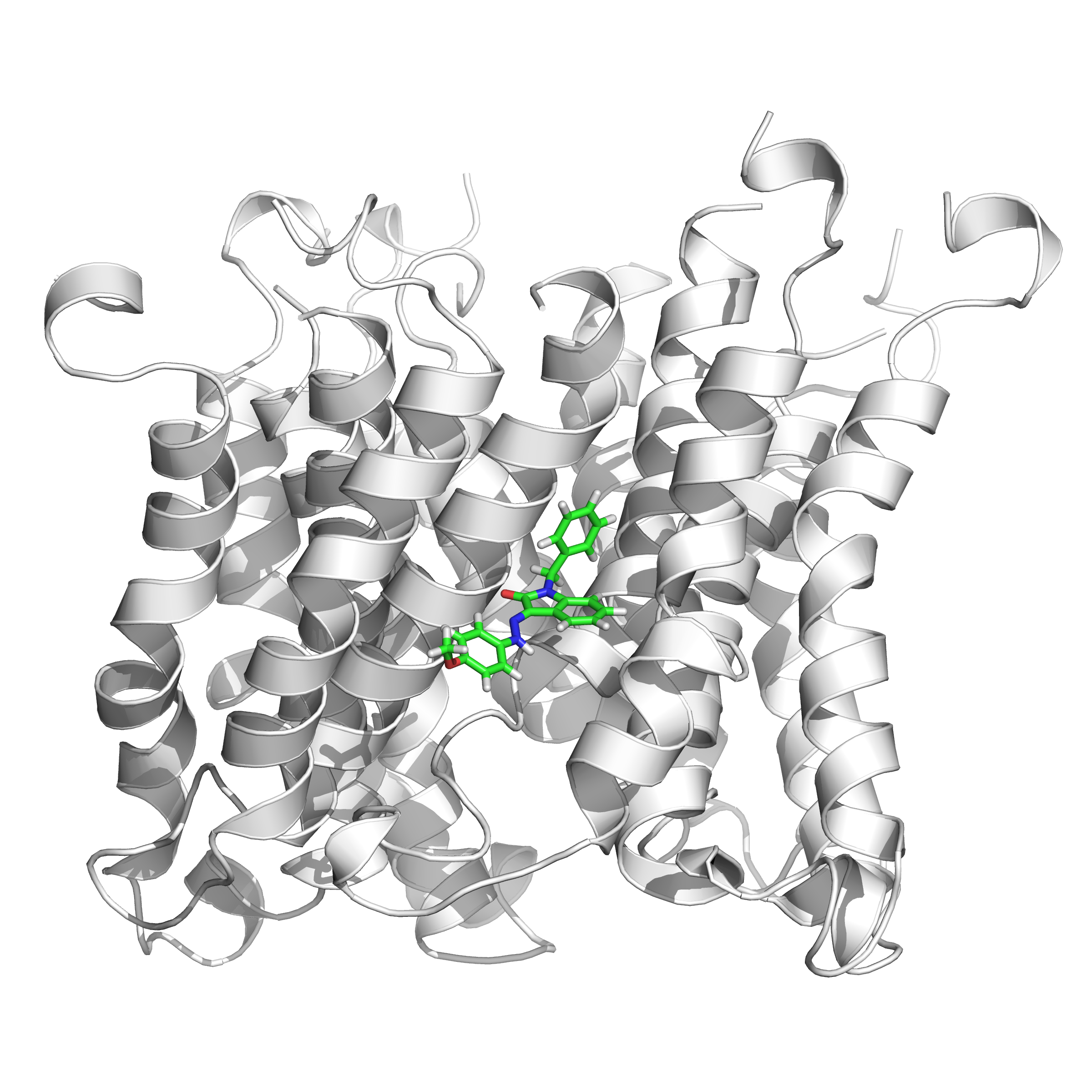

Supplement: Figure 3—source data 1. [file elife-52555-fig3-data1.zip › SD-figure3/new run files/100ns_side.png]

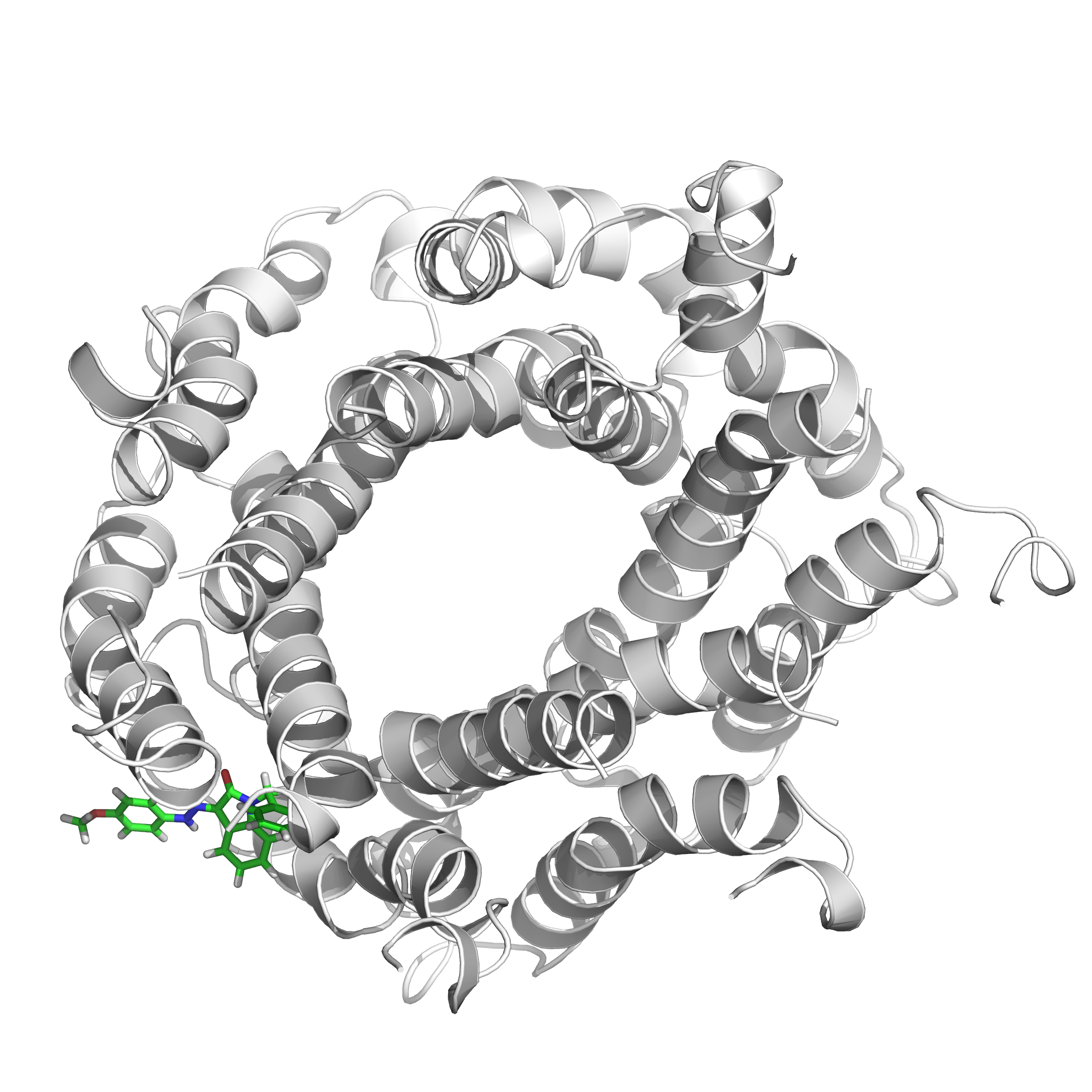

Supplement: Figure 3—source data 1. [file elife-52555-fig3-data1.zip › SD-figure3/new run files/100ns_top.png]

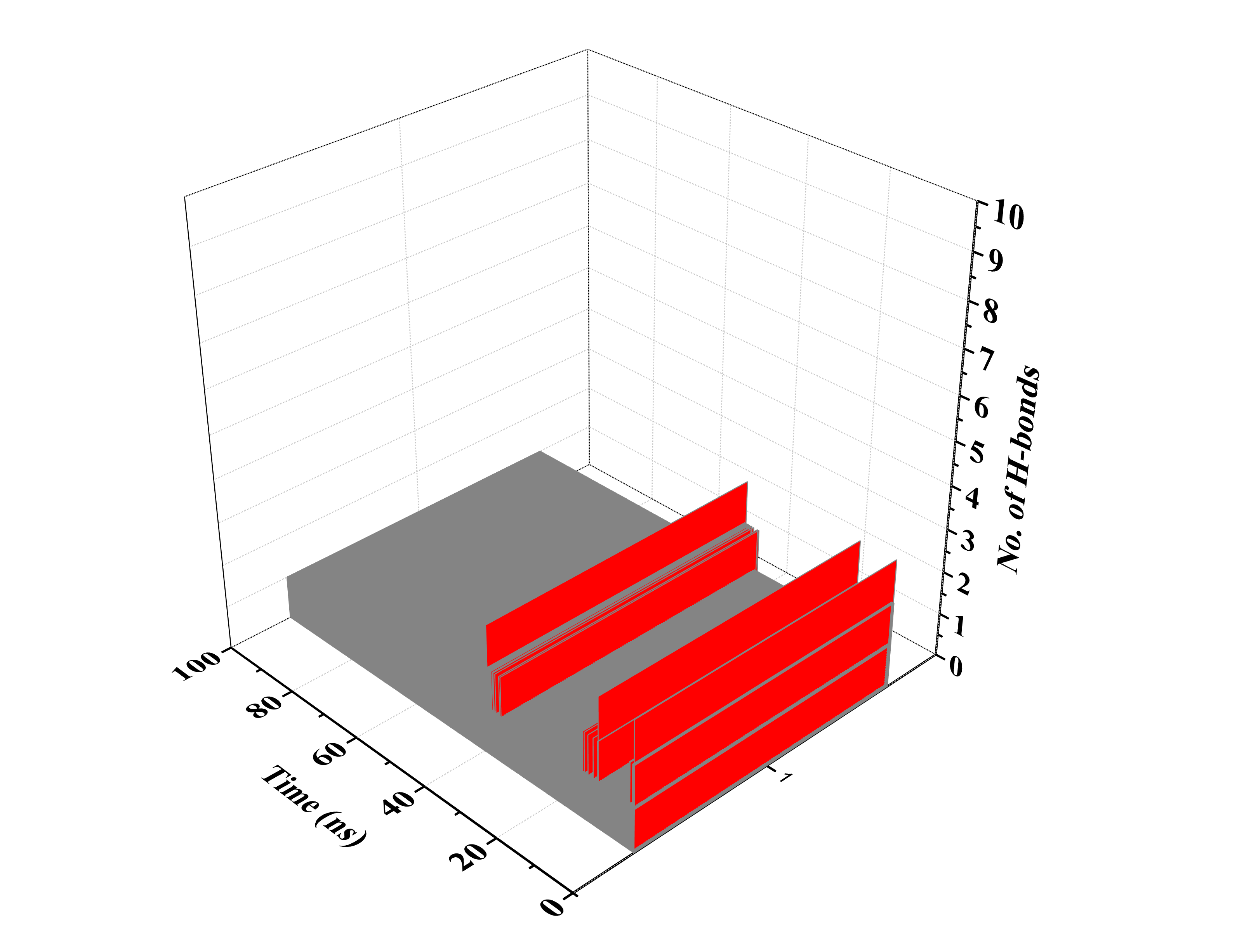

Supplement: Figure 3—source data 1. [file elife-52555-fig3-data1.zip › SD-figure3/new run files/H-bonds.png]

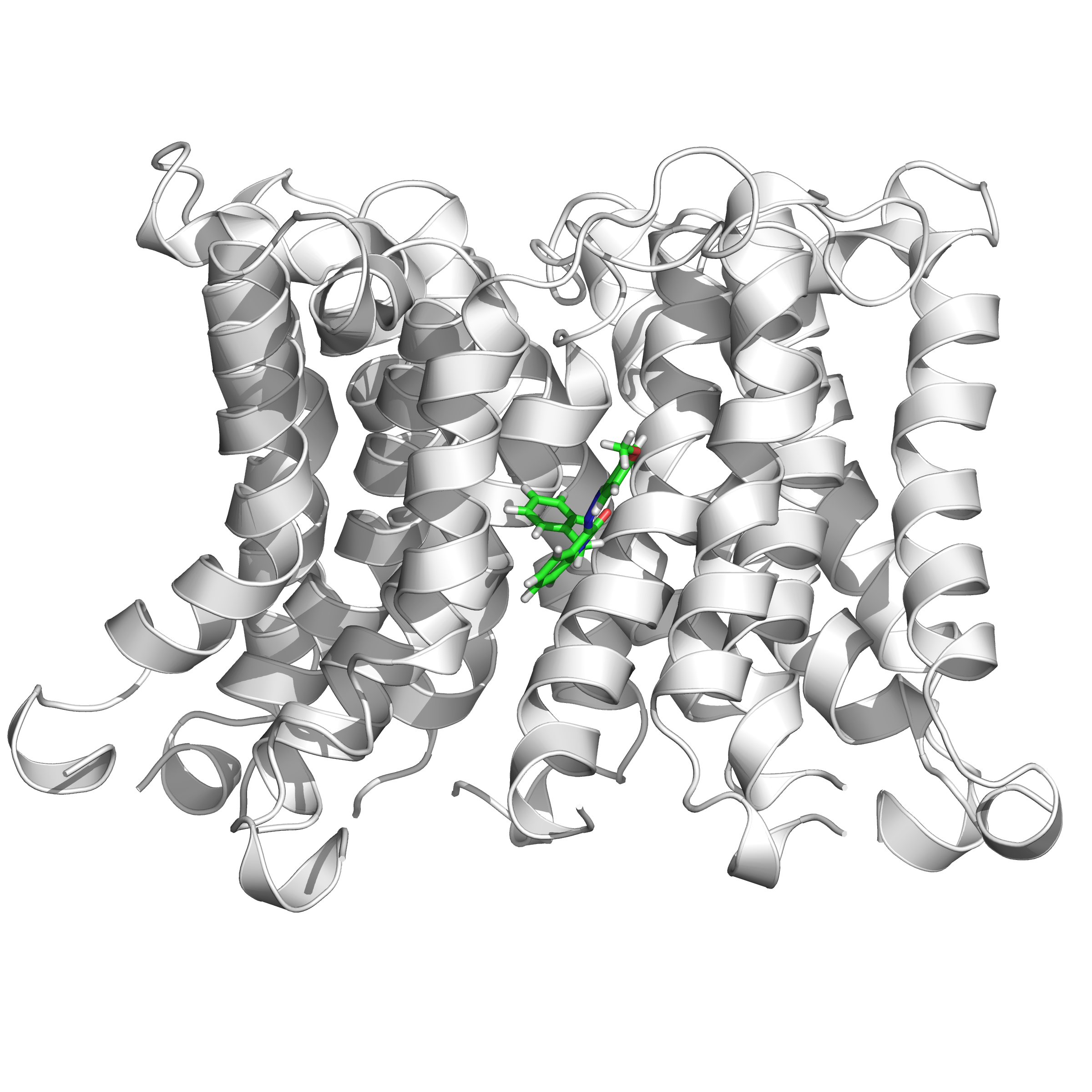

Supplement: Figure 3—source data 1. [file elife-52555-fig3-data1.zip › SD-figure3/new run files/Mutant/0ns_sideview.png]

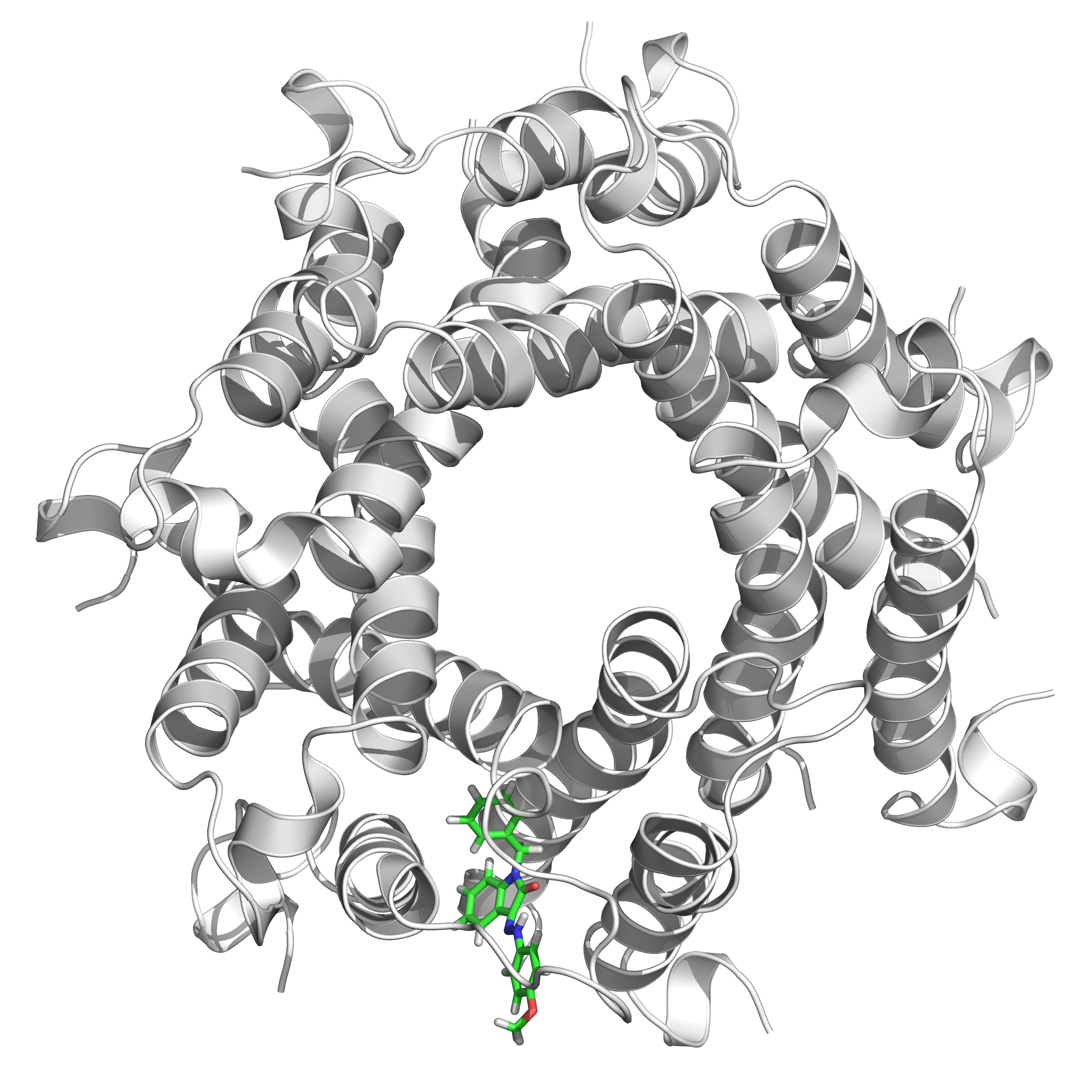

Supplement: Figure 3—source data 1. [file elife-52555-fig3-data1.zip › SD-figure3/new run files/Mutant/0ns_topview.png]

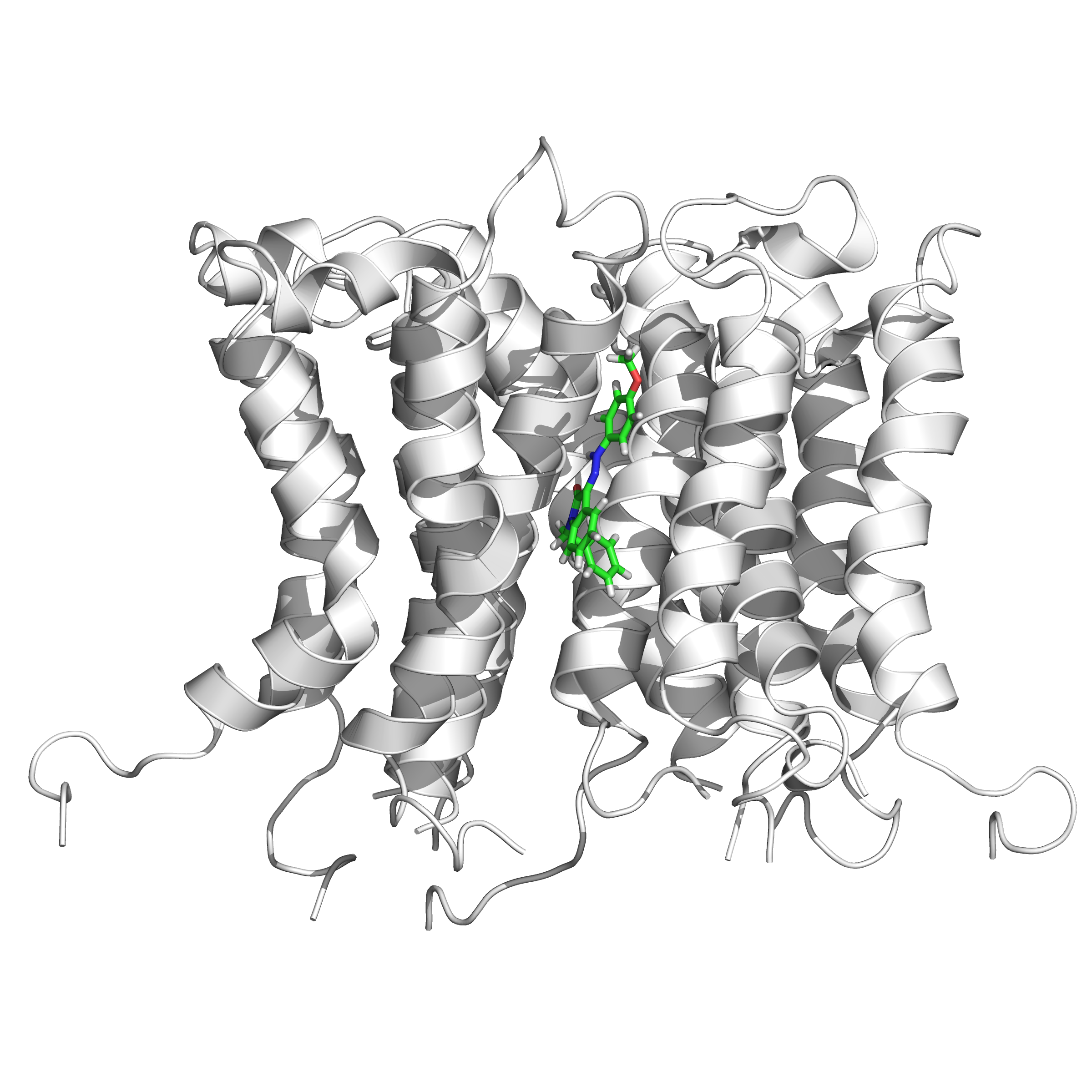

Supplement: Figure 3—source data 1. [file elife-52555-fig3-data1.zip › SD-figure3/new run files/Mutant/100ns_sideview.png]

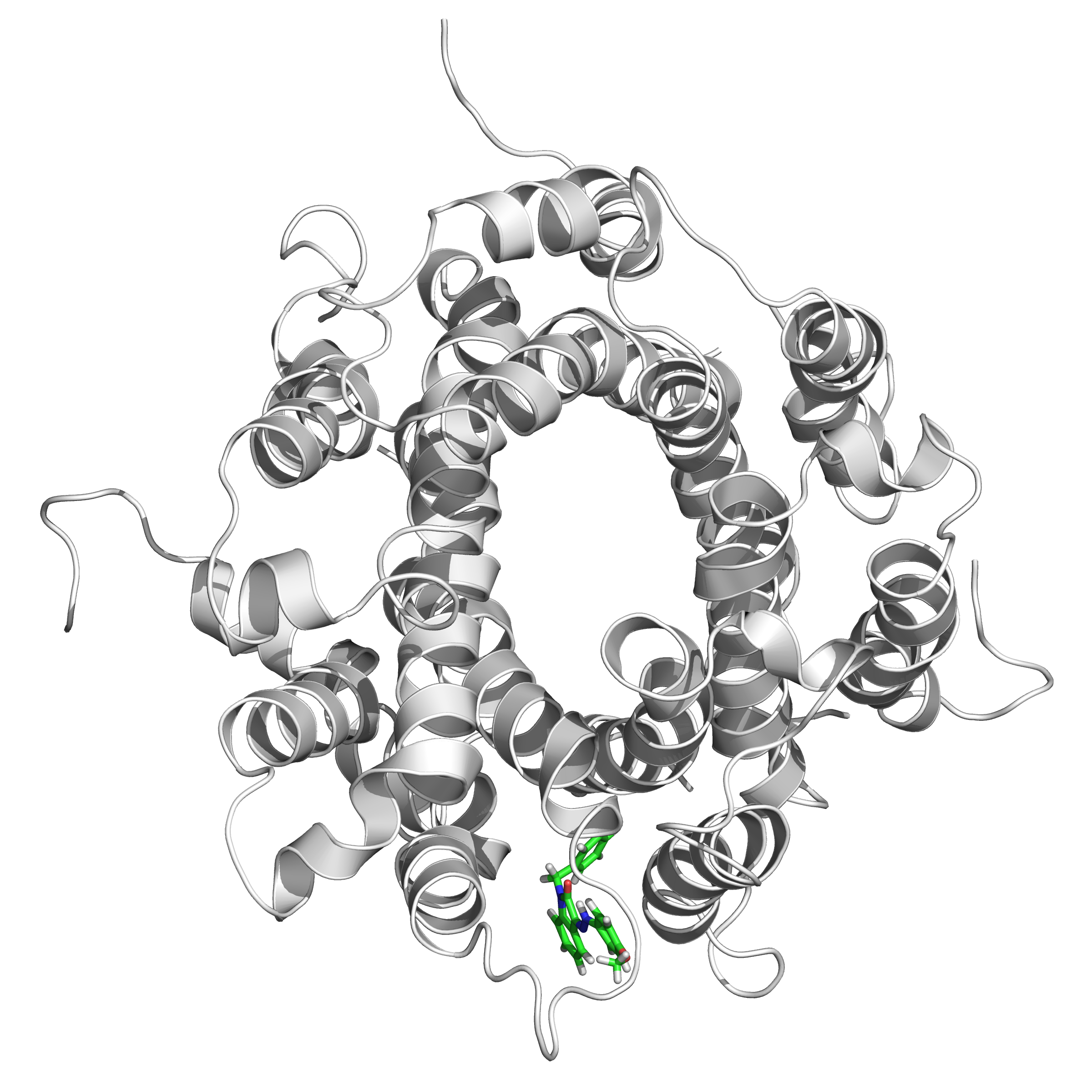

Supplement: Figure 3—source data 1. [file elife-52555-fig3-data1.zip › SD-figure3/new run files/Mutant/100ns_topview.png]

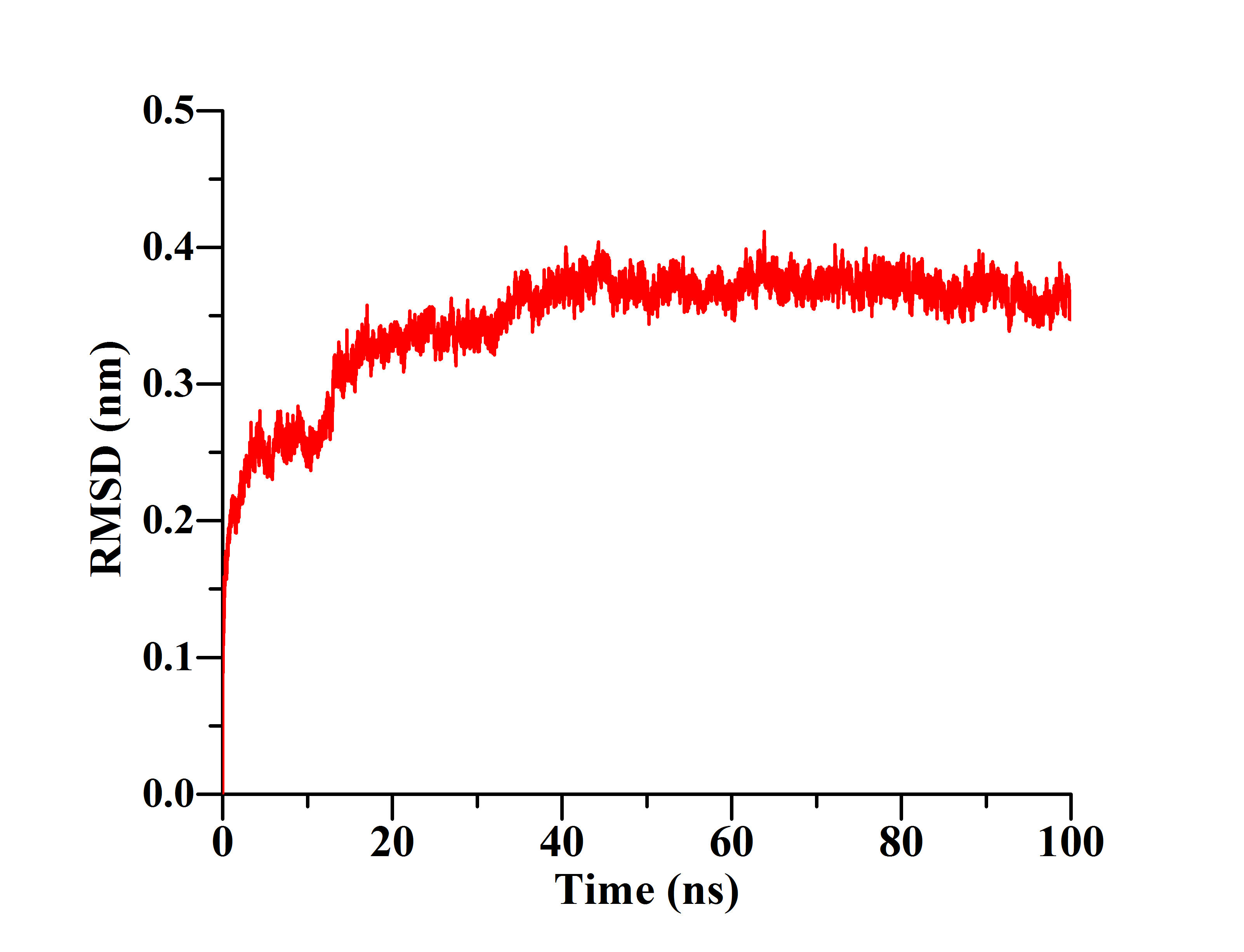

Supplement: Figure 3—source data 1. [file elife-52555-fig3-data1.zip › SD-figure3/new run files/P7_RMSD.png]

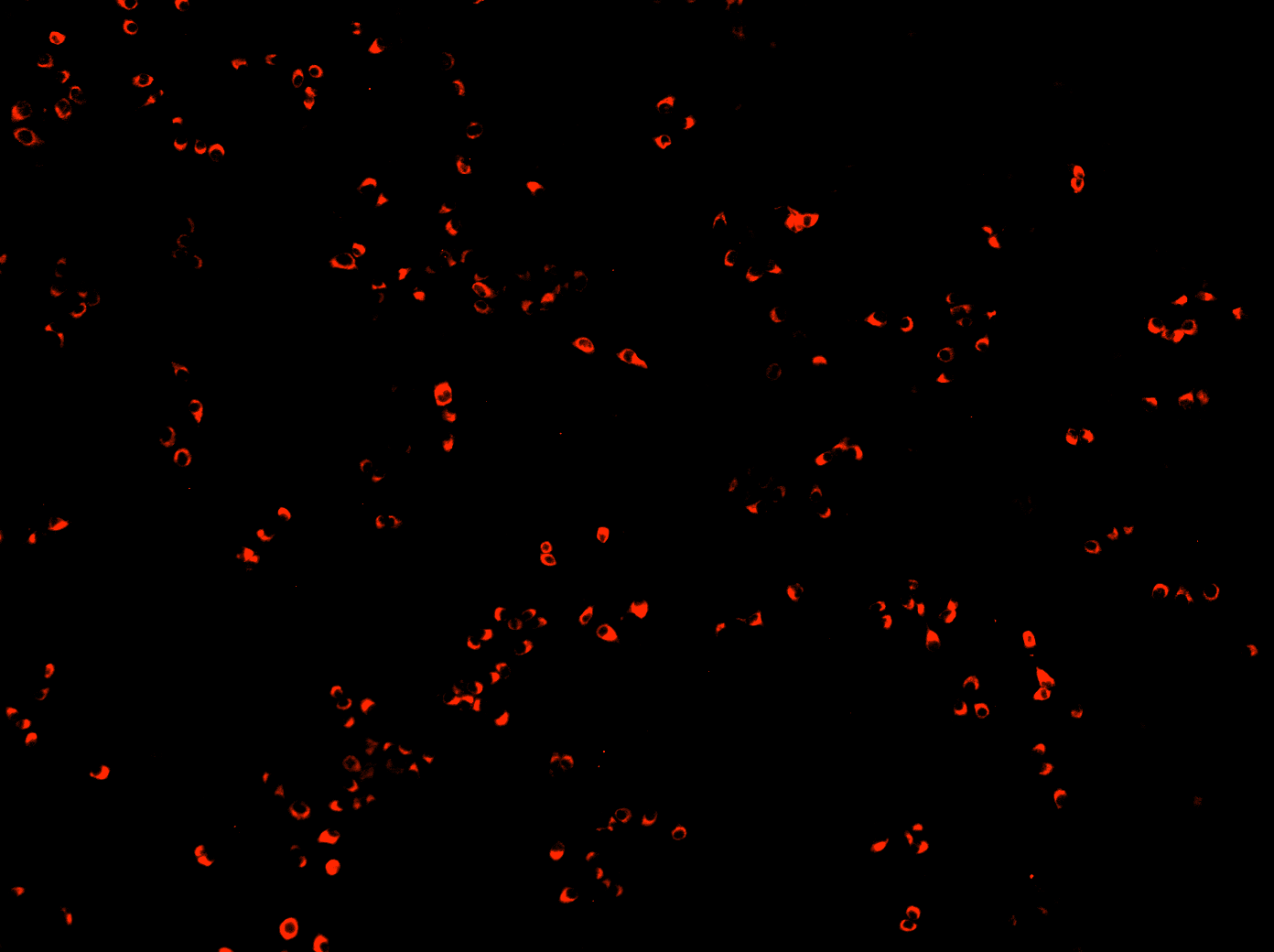

Supplement: Figure 4—source data 1. [file elife-52555-fig4-data1.zip › SD-figure4/B/DMSO control 18h RED ONLY.tif]

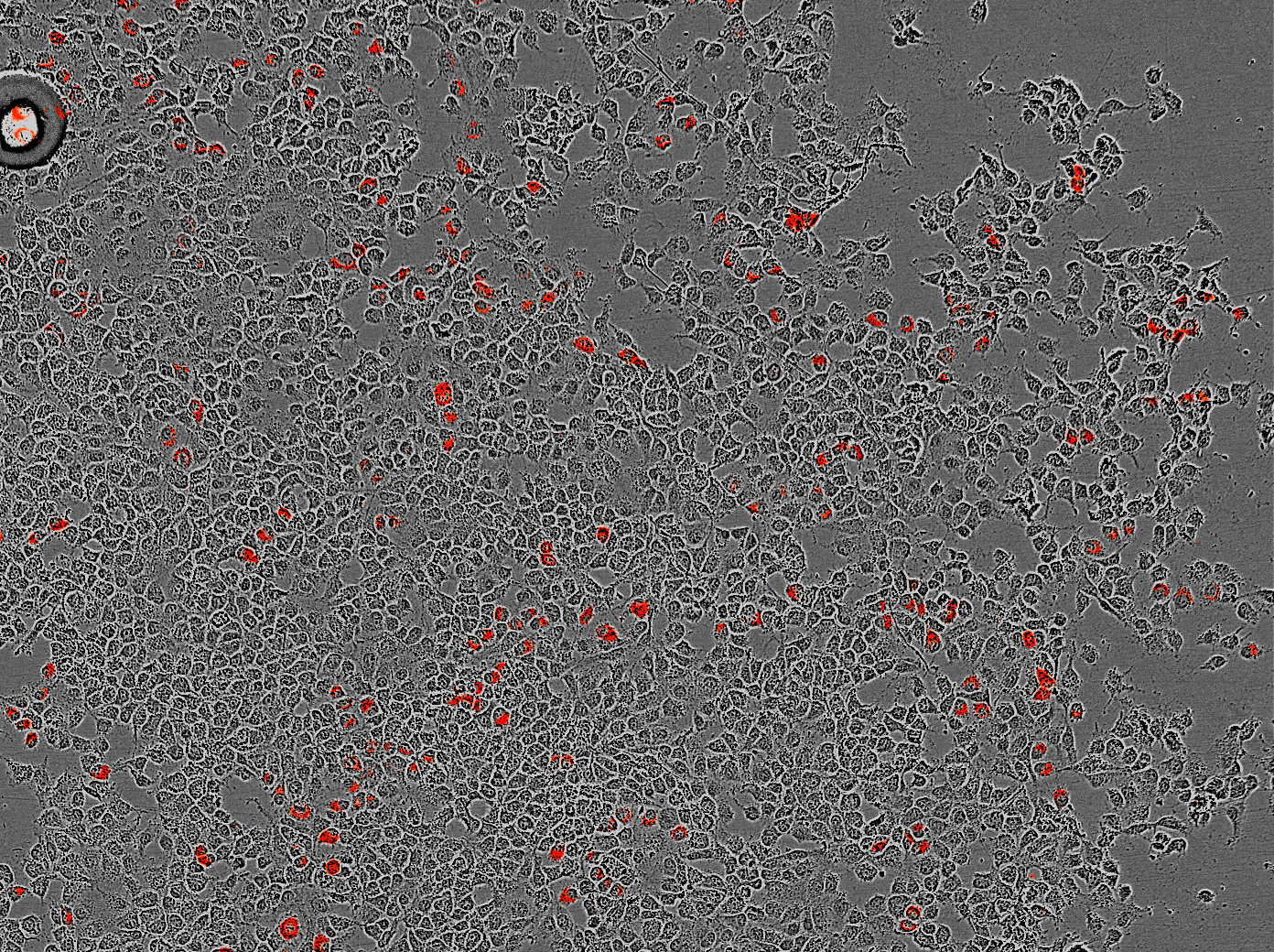

Supplement: Figure 4—source data 1. [file elife-52555-fig4-data1.zip › SD-figure4/B/DMSO control 18h.tif]

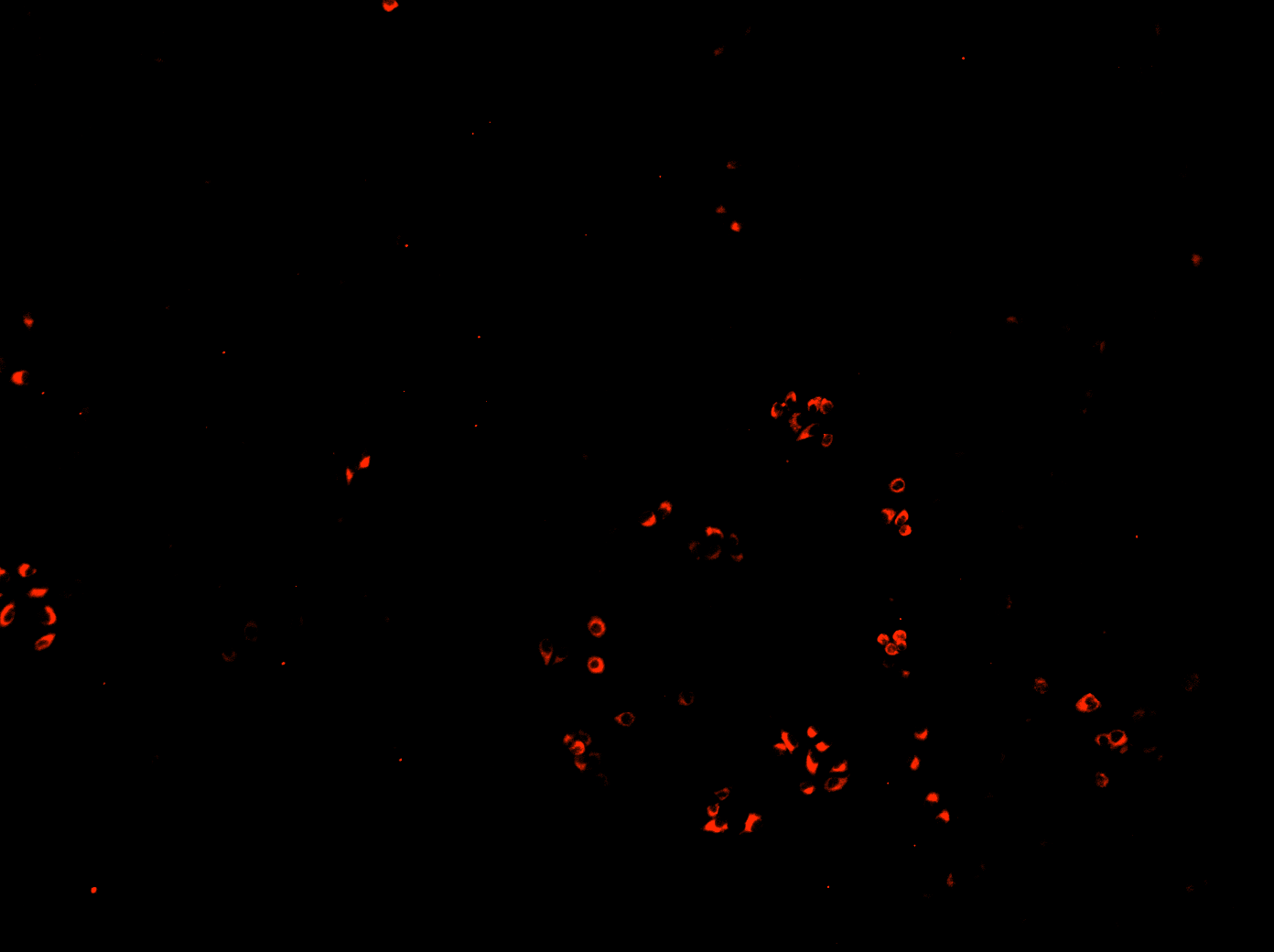

Supplement: Figure 4—source data 1. [file elife-52555-fig4-data1.zip › SD-figure4/B/JK3-32 4uM 18h RED ONLY.tif]

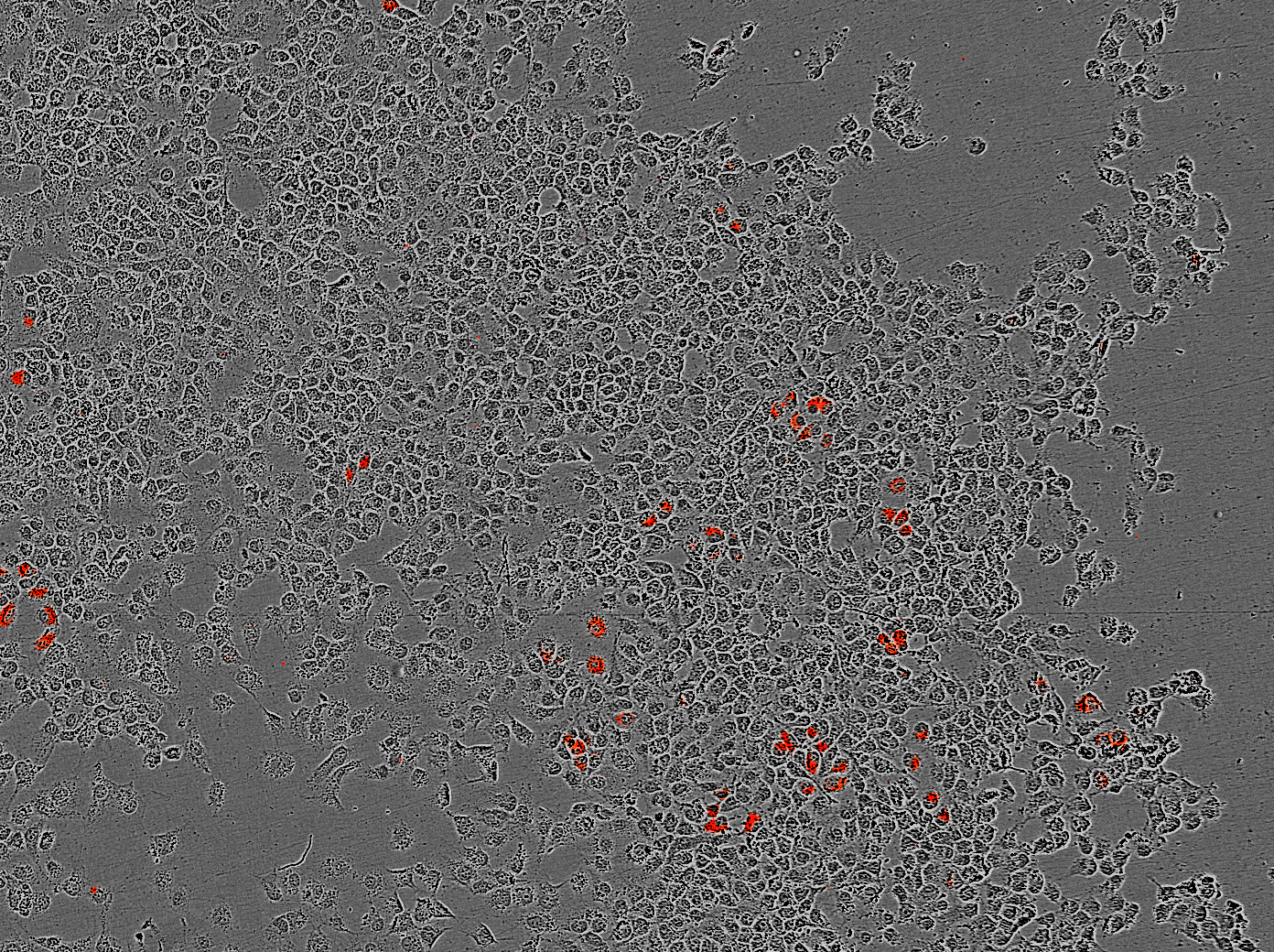

Supplement: Figure 4—source data 1. [file elife-52555-fig4-data1.zip › SD-figure4/B/JK3-32 4uM 18h.tif]

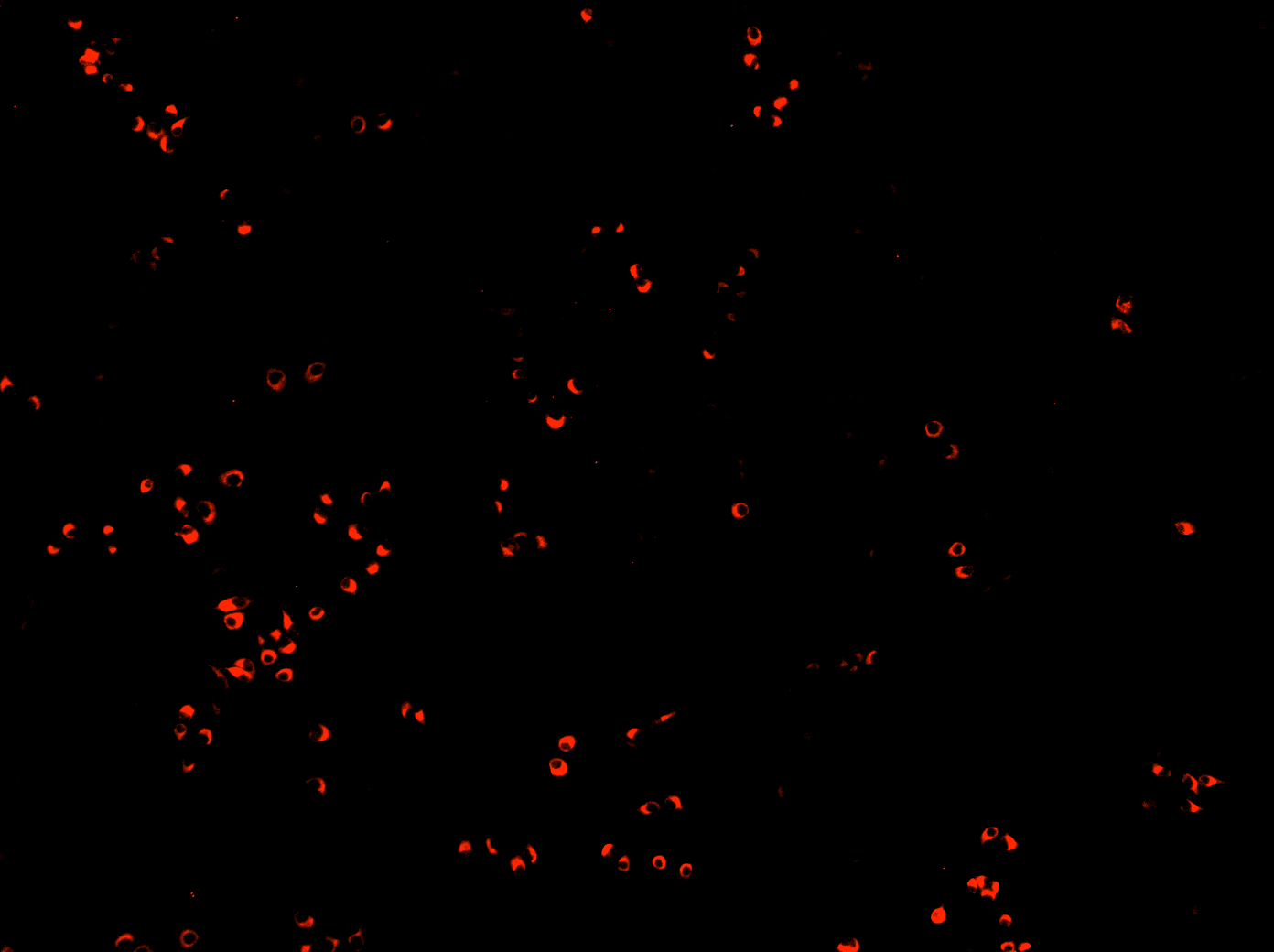

Supplement: Figure 4—source data 1. [file elife-52555-fig4-data1.zip › SD-figure4/B/R21 DMSO control 18h RED ONLY.tif]

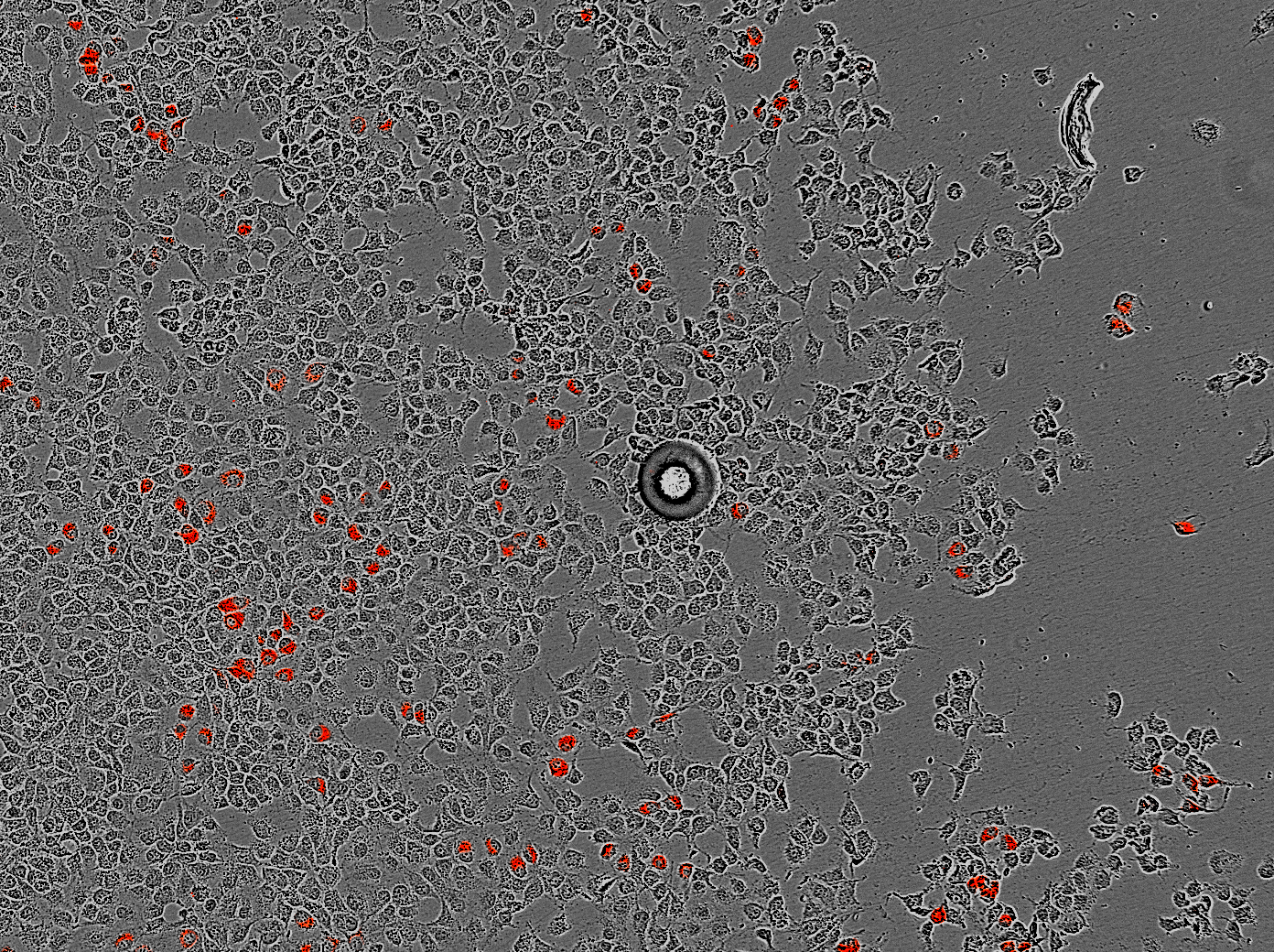

Supplement: Figure 4—source data 1. [file elife-52555-fig4-data1.zip › SD-figure4/B/R21 DMSO control 18h.tif]

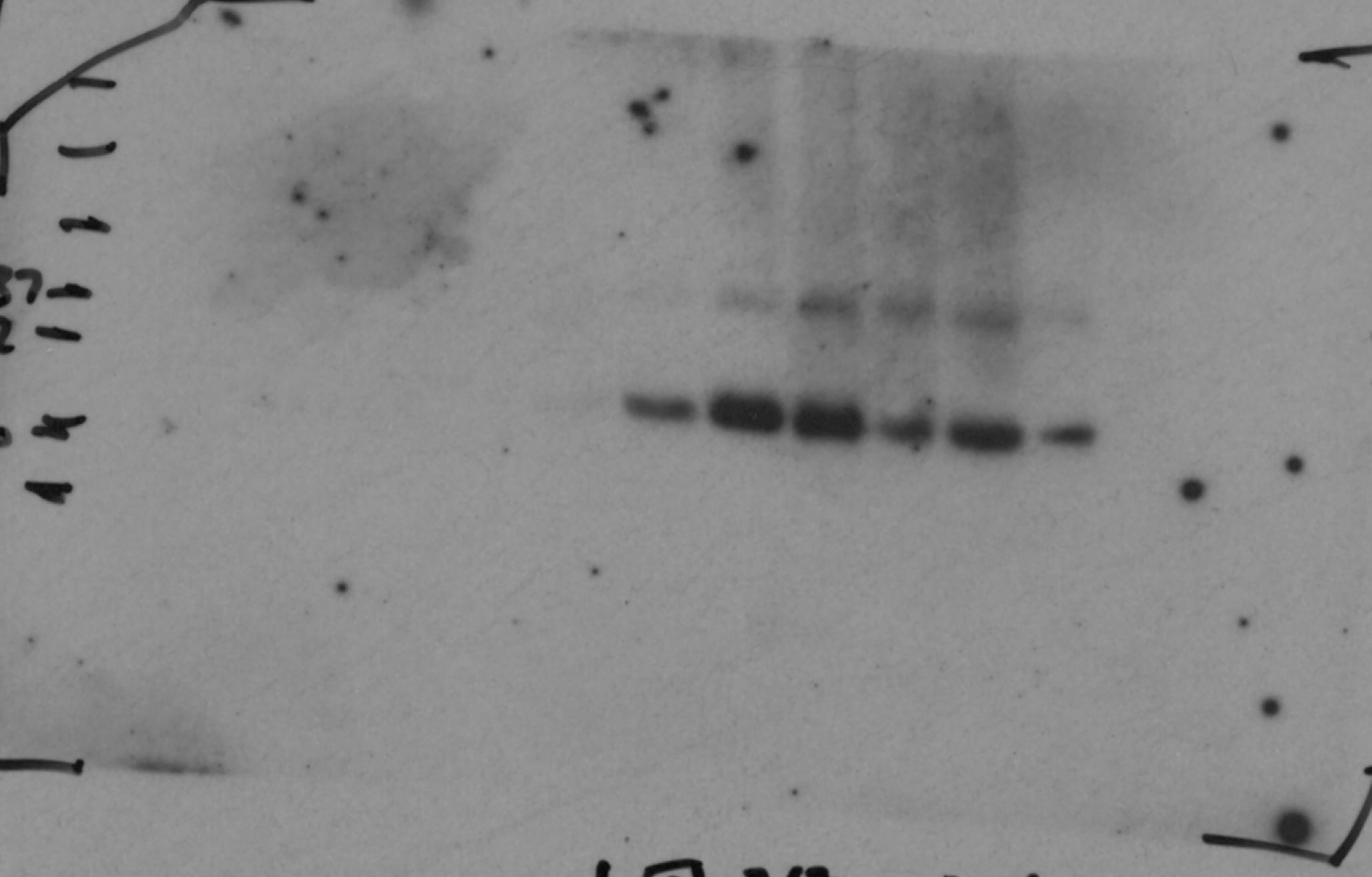

Supplement: Figure 5—source data 1. [file elife-52555-fig5-data1.zip › SD-figure5/JK332-488expt2.tif]

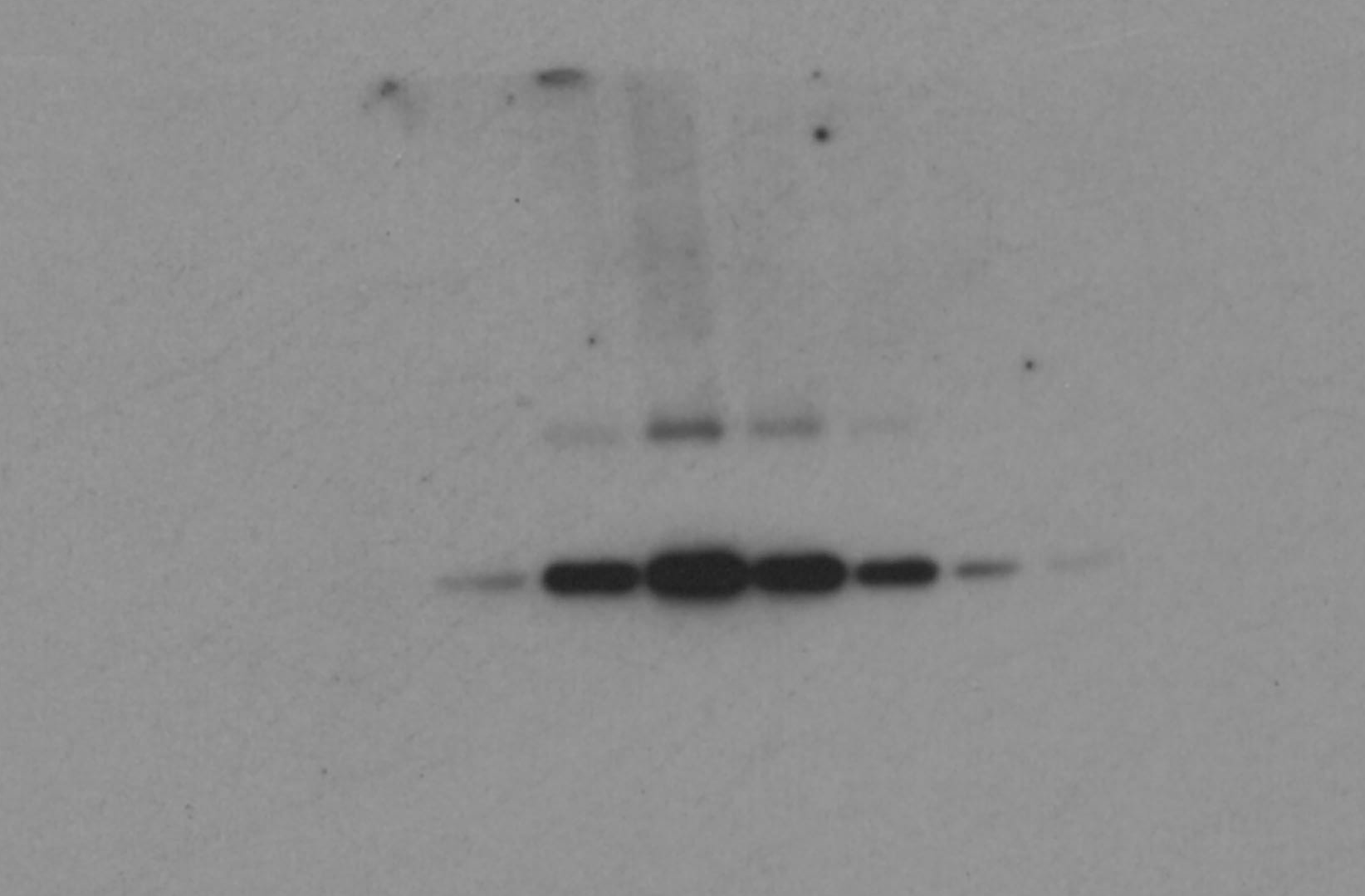

Supplement: Figure 5—source data 1. [file elife-52555-fig5-data1.zip › SD-figure5/JK332expt2v2.tif]

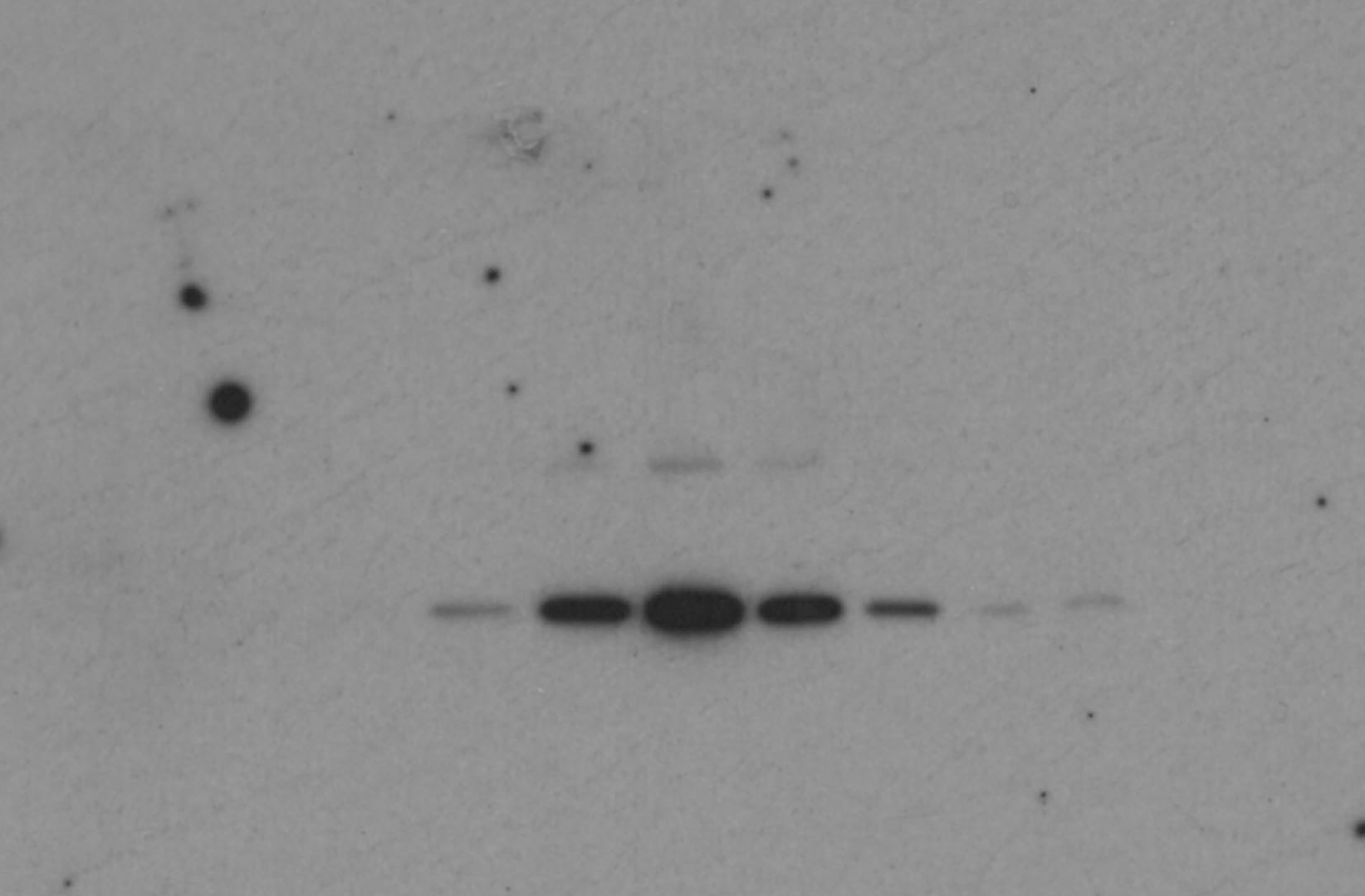

Supplement: Figure 5—source data 1. [file elife-52555-fig5-data1.zip › SD-figure5/R21expt2v2.tif]

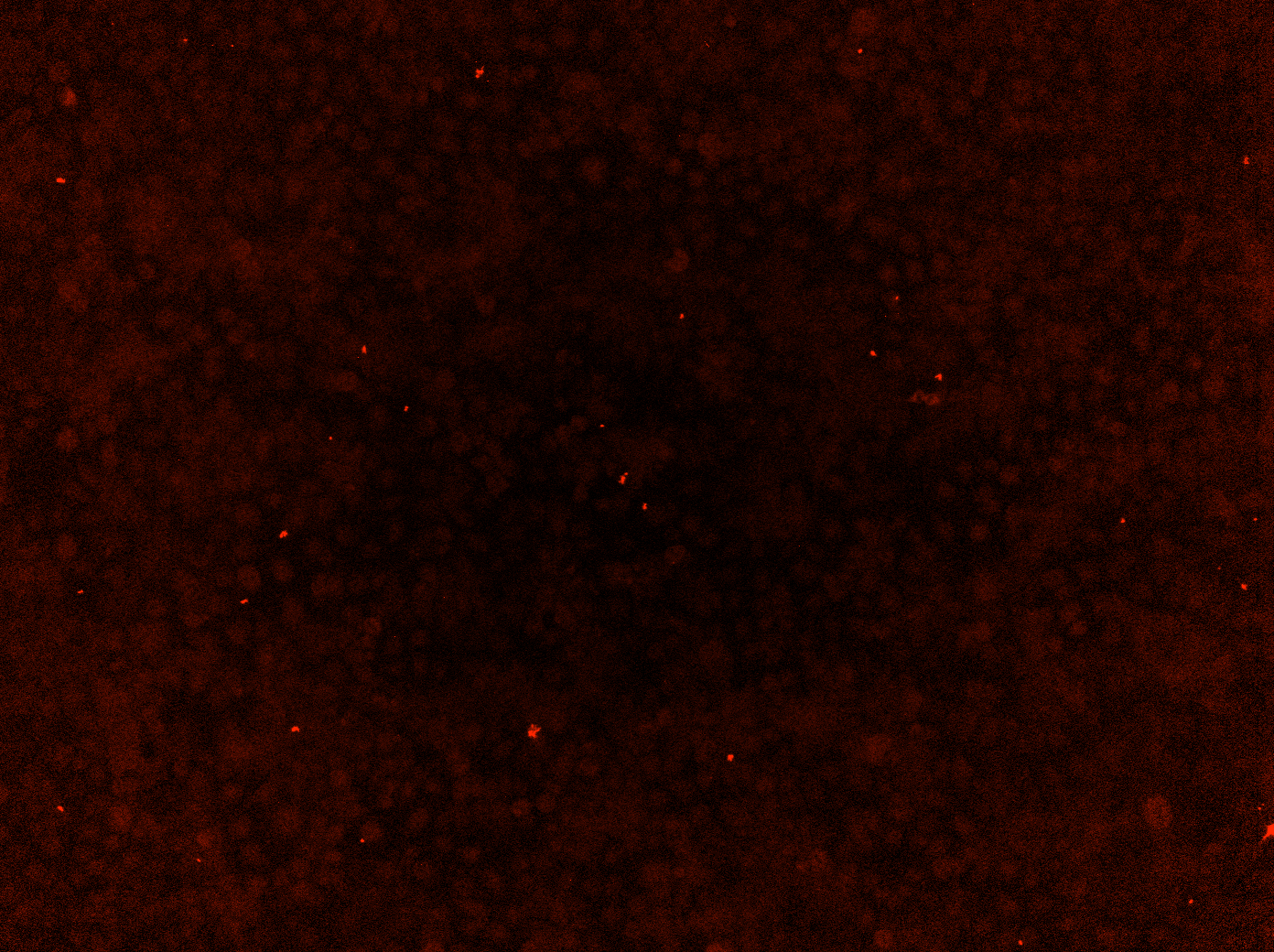

Supplement: Figure 7—figure supplement 1—source data 1. [file elife-52555-fig7-figsupp1-data1.zip › SD-figureS9/Steve G images/JK332-488/SteveG_F10_1.tif]

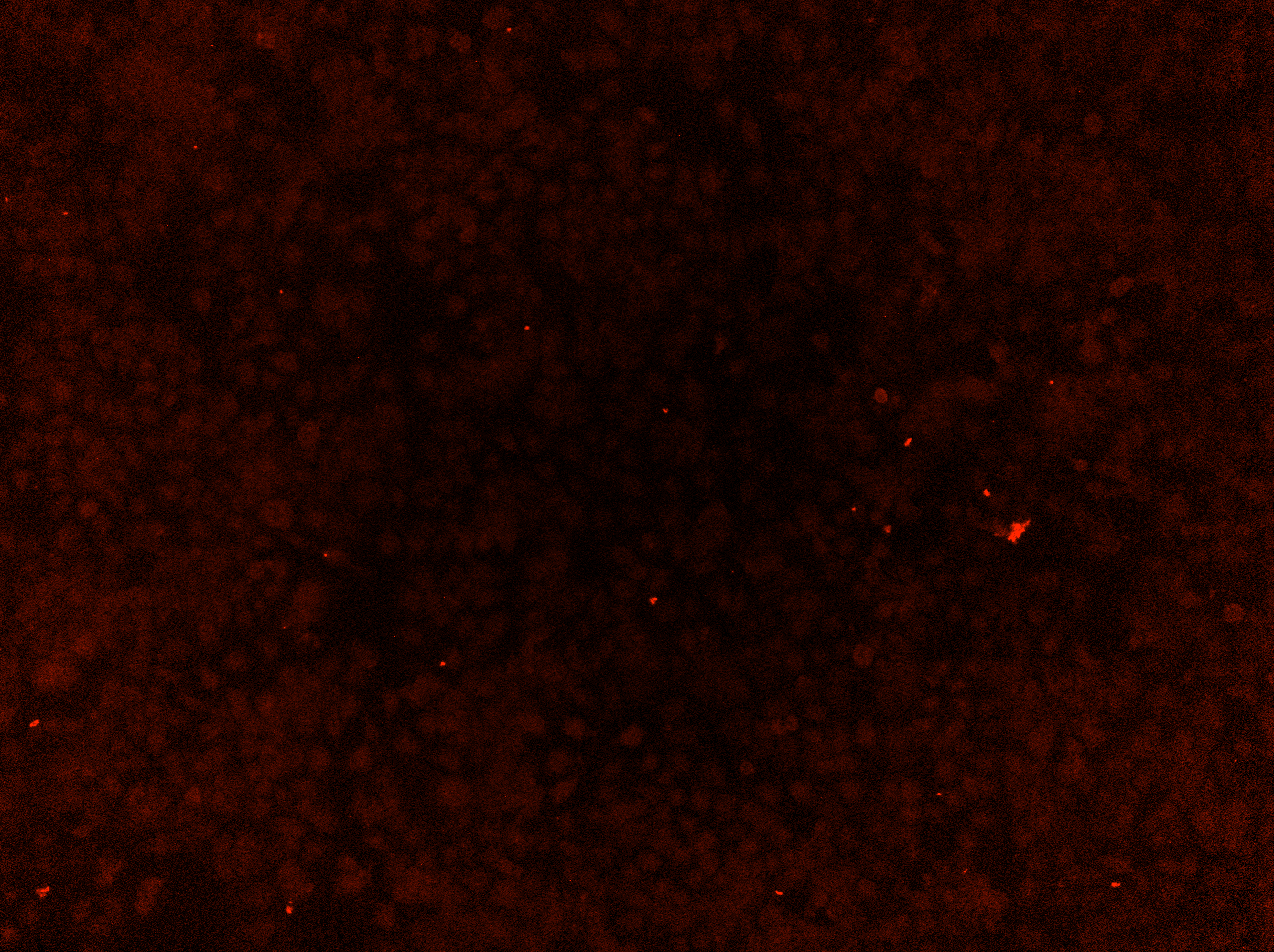

Supplement: Figure 7—figure supplement 1—source data 1. [file elife-52555-fig7-figsupp1-data1.zip › SD-figureS9/Steve G images/JK332-488/SteveG_F11_1.tif]

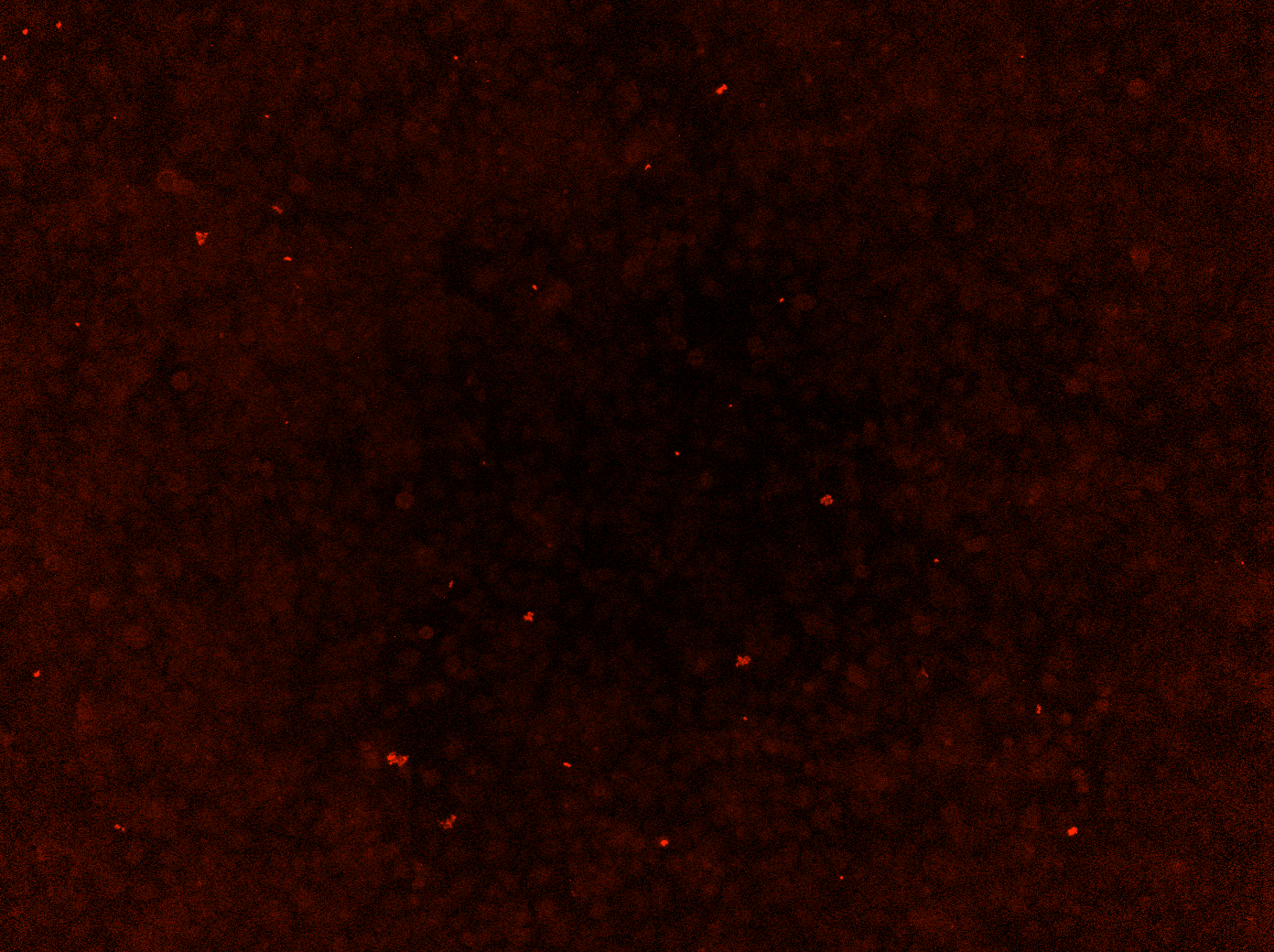

Supplement: Figure 7—figure supplement 1—source data 1. [file elife-52555-fig7-figsupp1-data1.zip › SD-figureS9/Steve G images/JK332-488/SteveG_F12_1.tif]

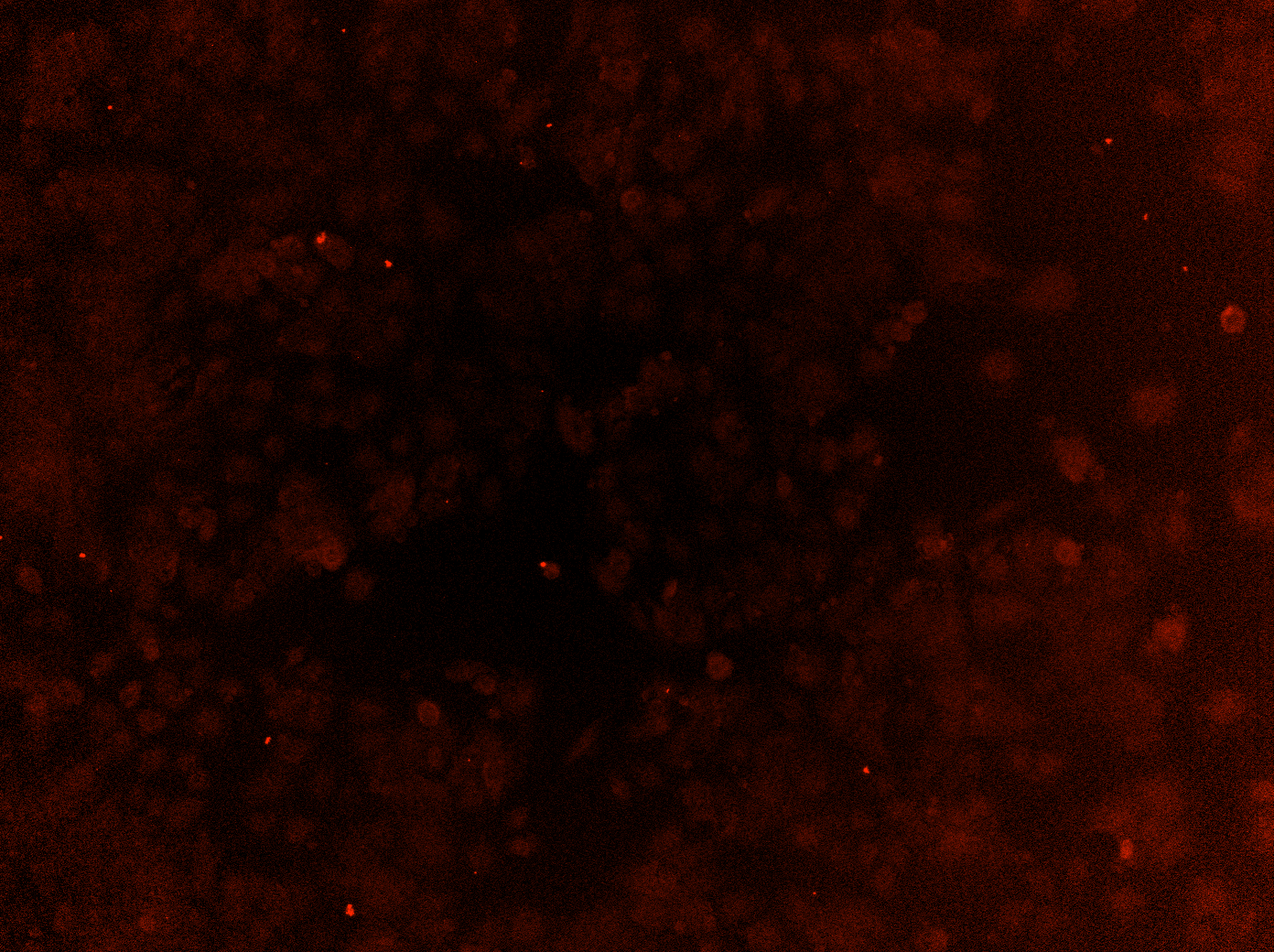

Supplement: Figure 7—figure supplement 1—source data 1. [file elife-52555-fig7-figsupp1-data1.zip › SD-figureS9/Steve G images/JK332-488/SteveG_F1_1.tif]

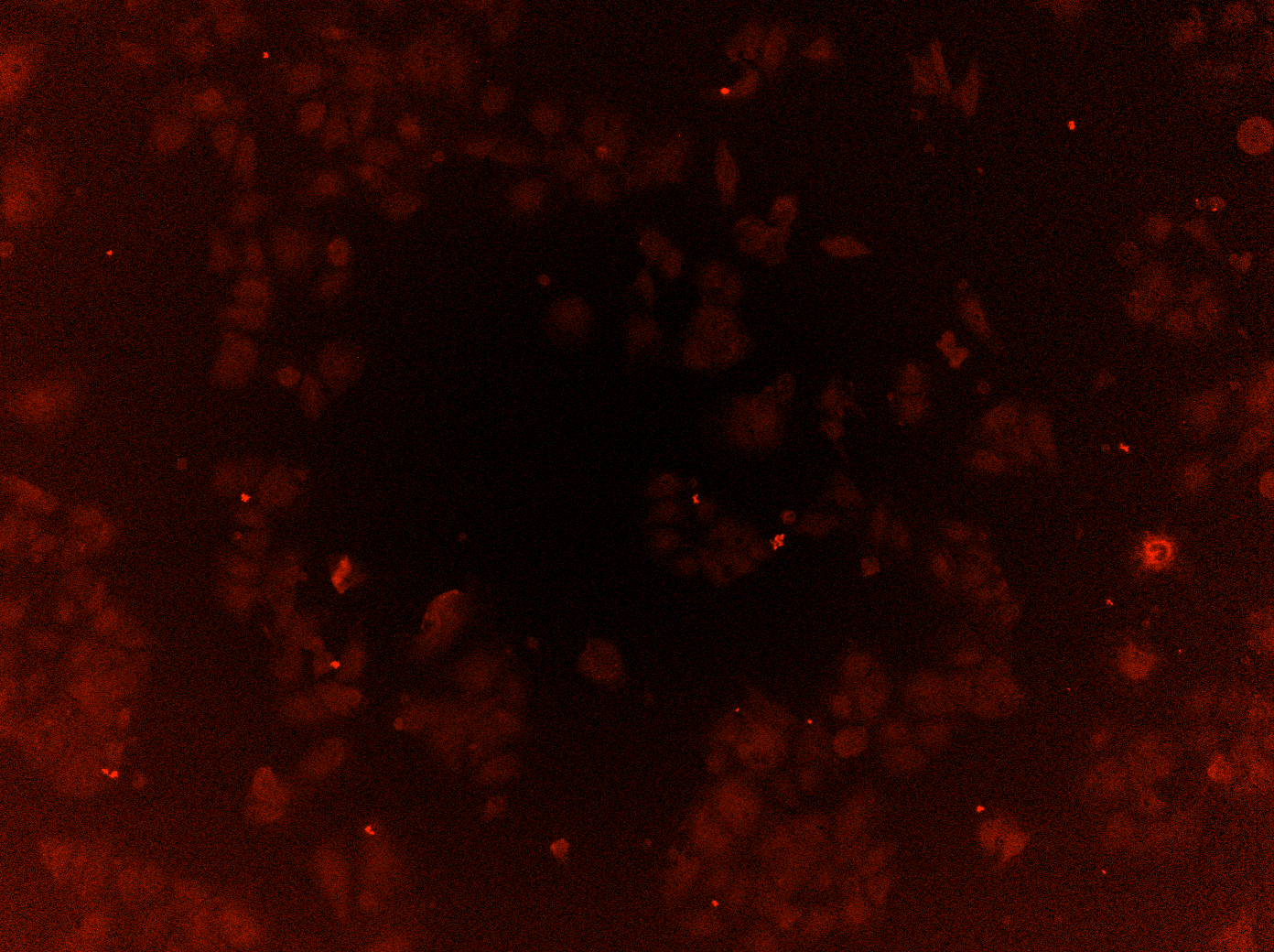

Supplement: Figure 7—figure supplement 1—source data 1. [file elife-52555-fig7-figsupp1-data1.zip › SD-figureS9/Steve G images/JK332-488/SteveG_F2_1.tif]

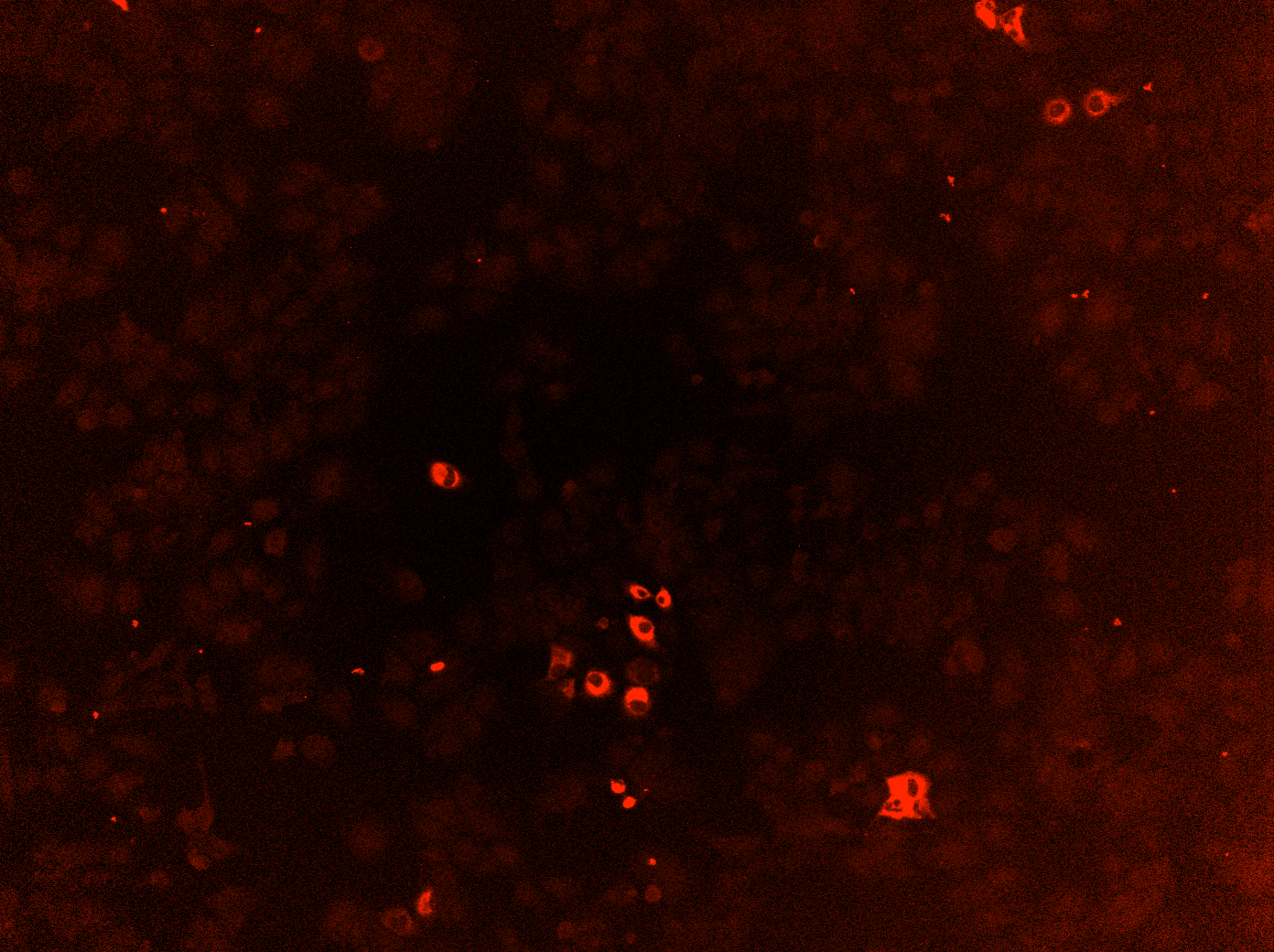

Supplement: Figure 7—figure supplement 1—source data 1. [file elife-52555-fig7-figsupp1-data1.zip › SD-figureS9/Steve G images/JK332-488/SteveG_F3_1.tif]

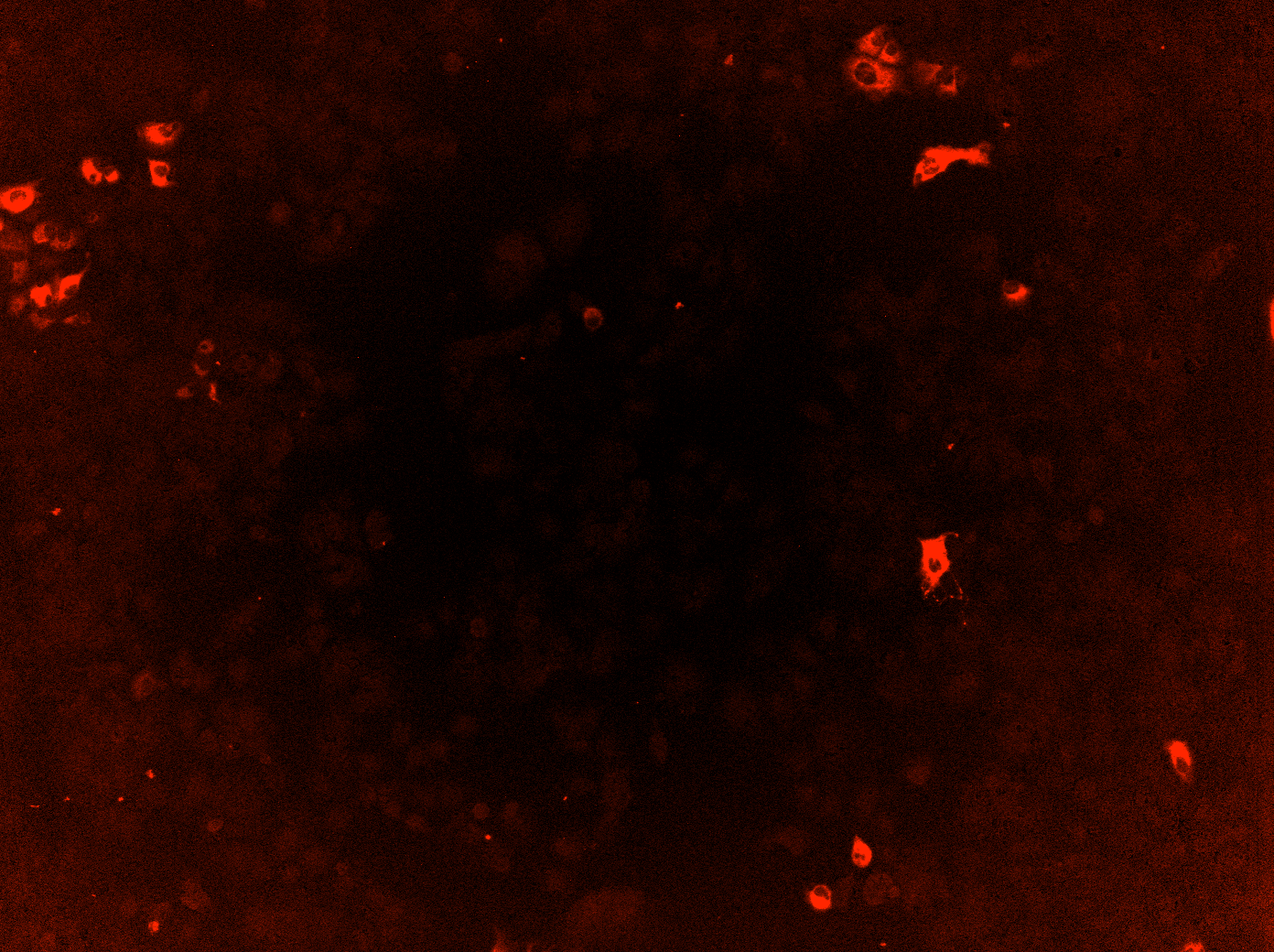

Supplement: Figure 7—figure supplement 1—source data 1. [file elife-52555-fig7-figsupp1-data1.zip › SD-figureS9/Steve G images/JK332-488/SteveG_F4_1.tif]

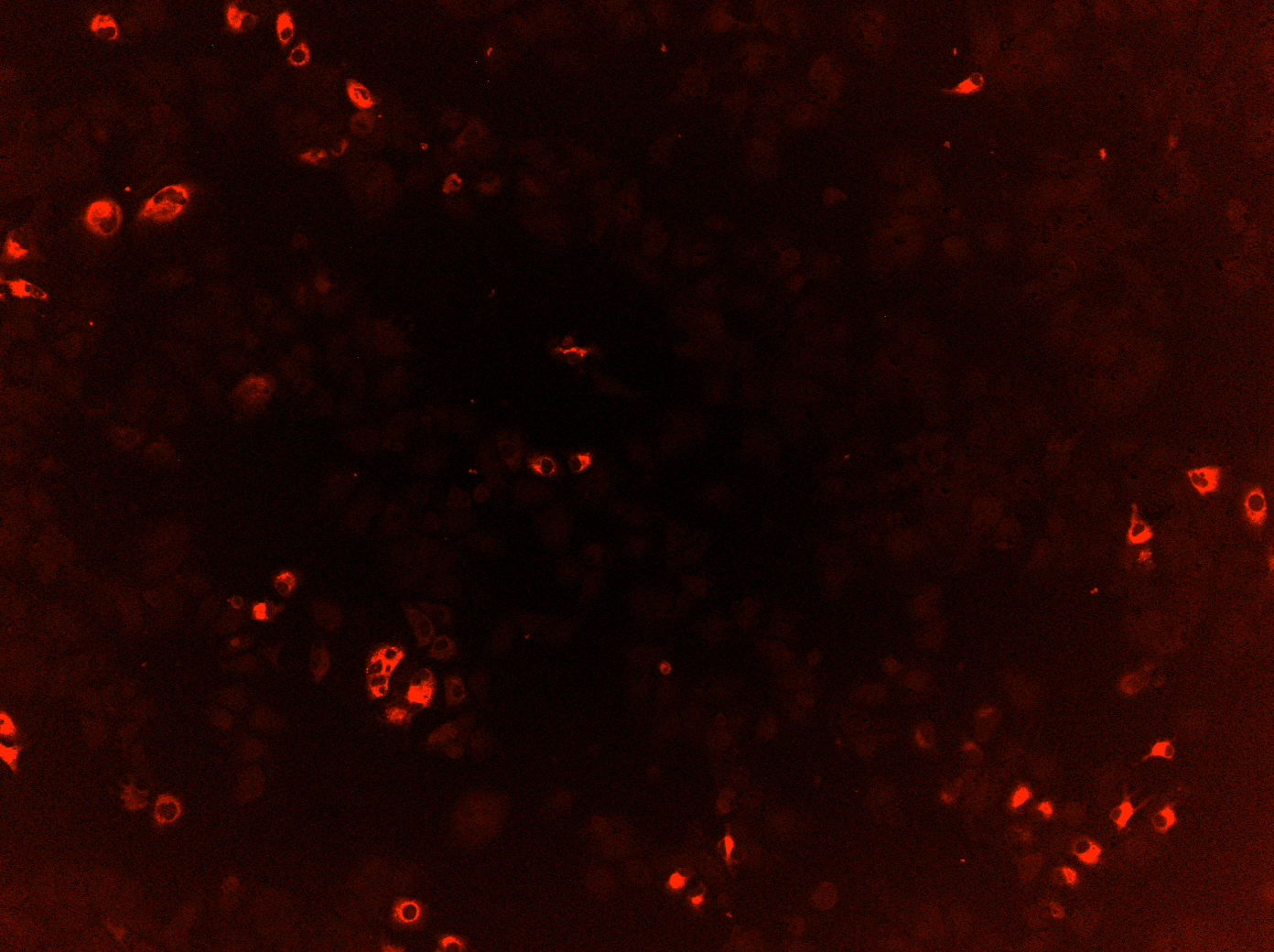

Supplement: Figure 7—figure supplement 1—source data 1. [file elife-52555-fig7-figsupp1-data1.zip › SD-figureS9/Steve G images/JK332-488/SteveG_F5_1.tif]

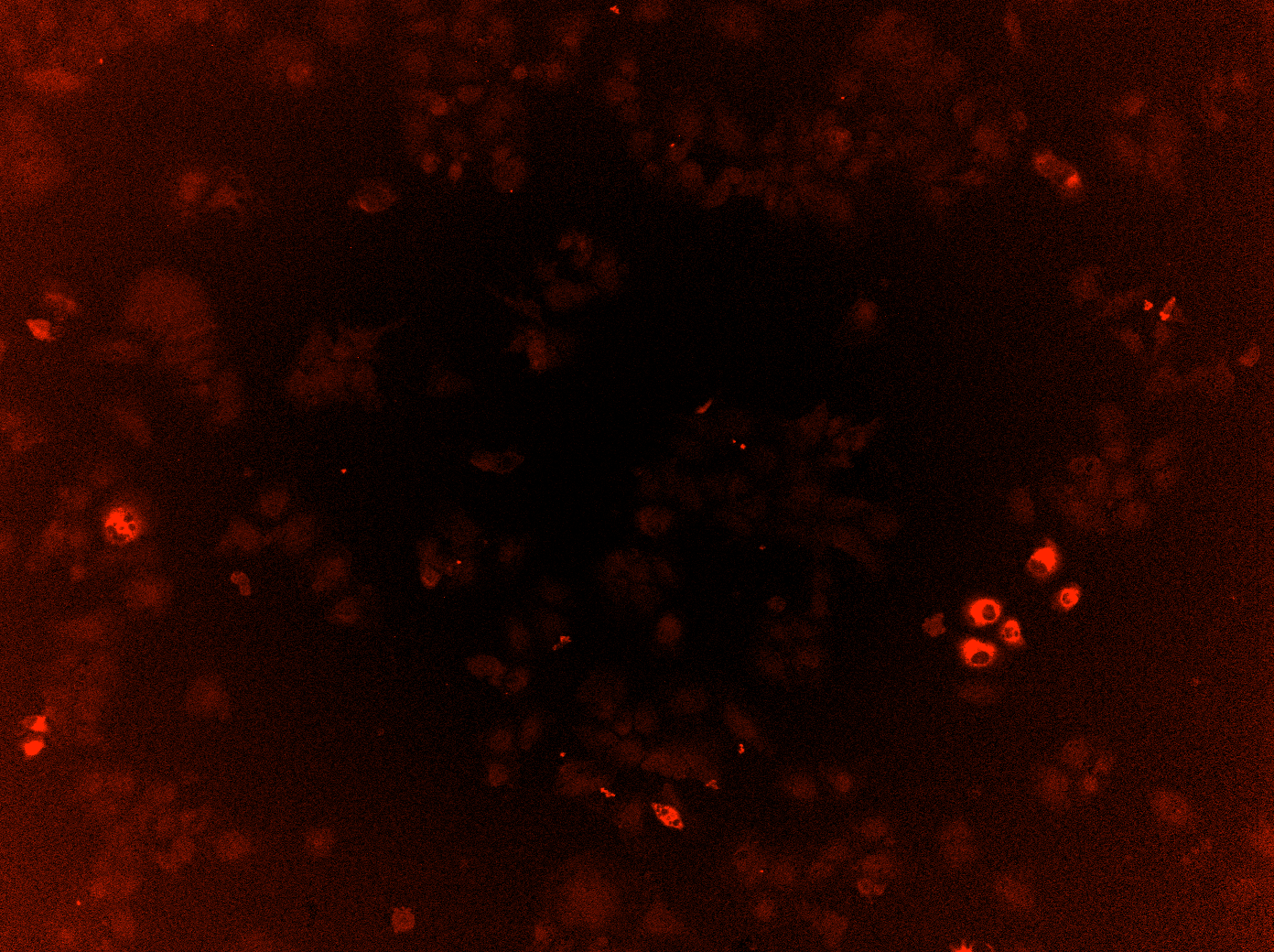

Supplement: Figure 7—figure supplement 1—source data 1. [file elife-52555-fig7-figsupp1-data1.zip › SD-figureS9/Steve G images/JK332-488/SteveG_F6_1.tif]

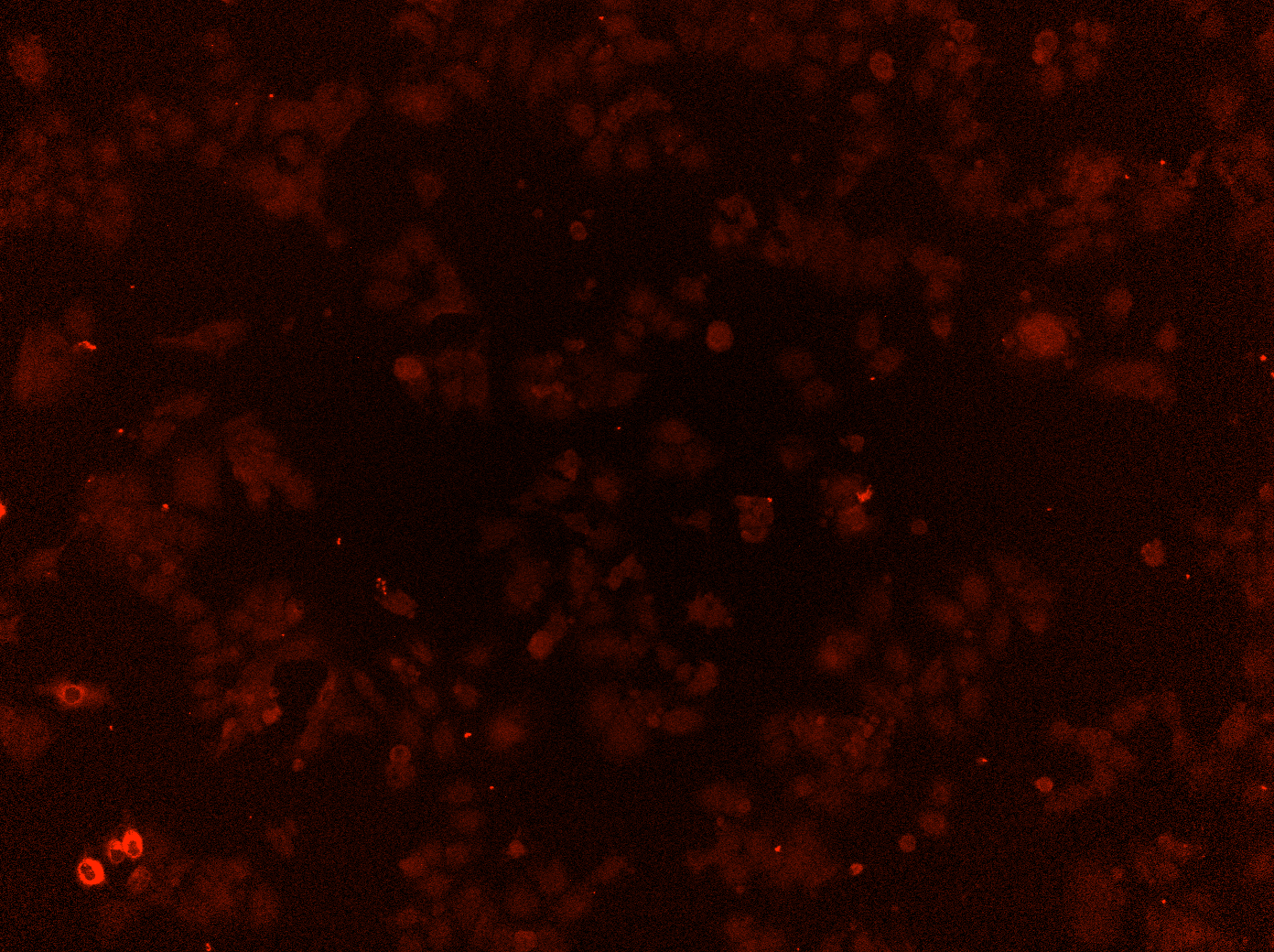

Supplement: Figure 7—figure supplement 1—source data 1. [file elife-52555-fig7-figsupp1-data1.zip › SD-figureS9/Steve G images/JK332-488/SteveG_F7_1.tif]

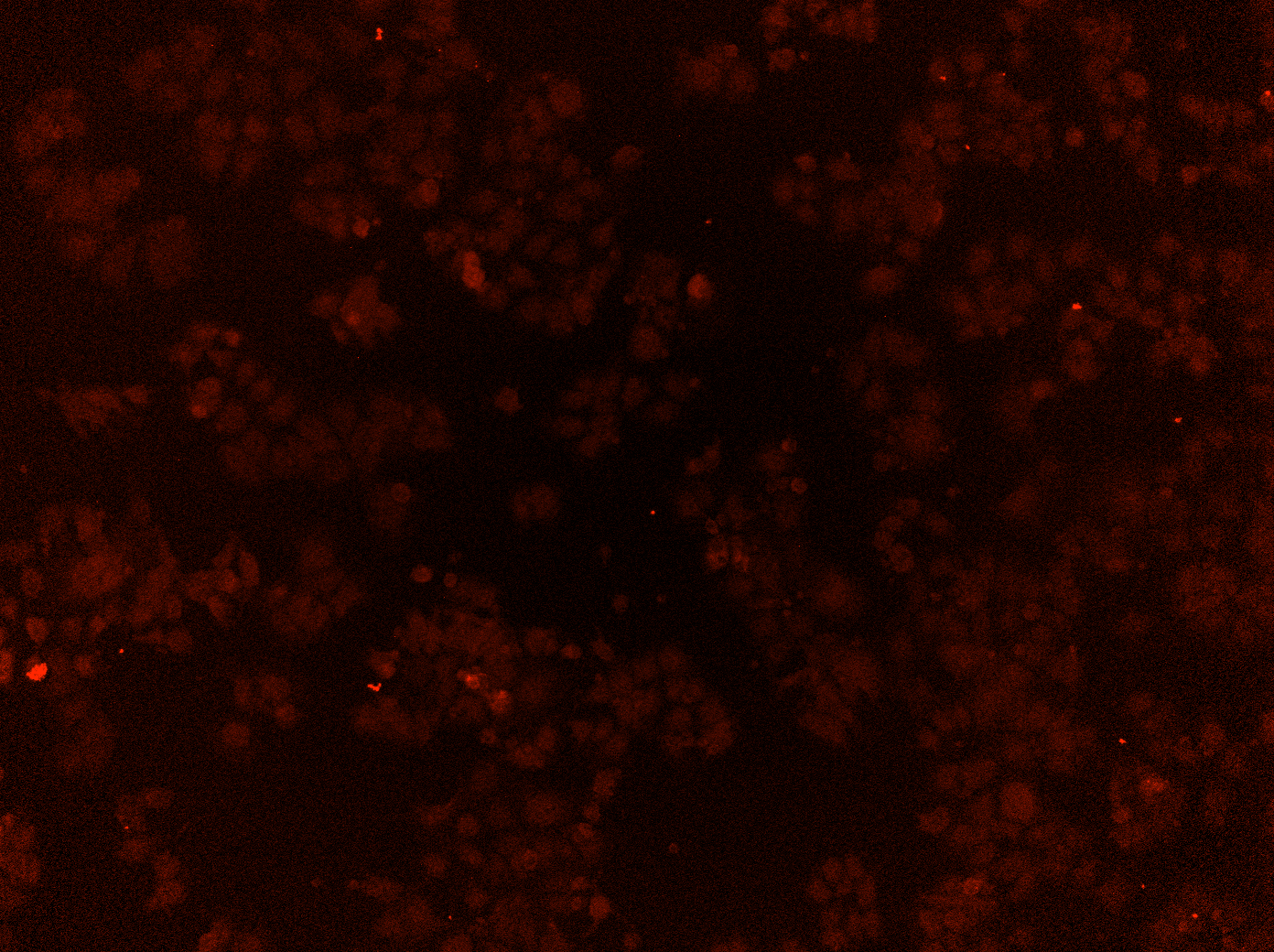

Supplement: Figure 7—figure supplement 1—source data 1. [file elife-52555-fig7-figsupp1-data1.zip › SD-figureS9/Steve G images/JK332-488/SteveG_F8_1.tif]

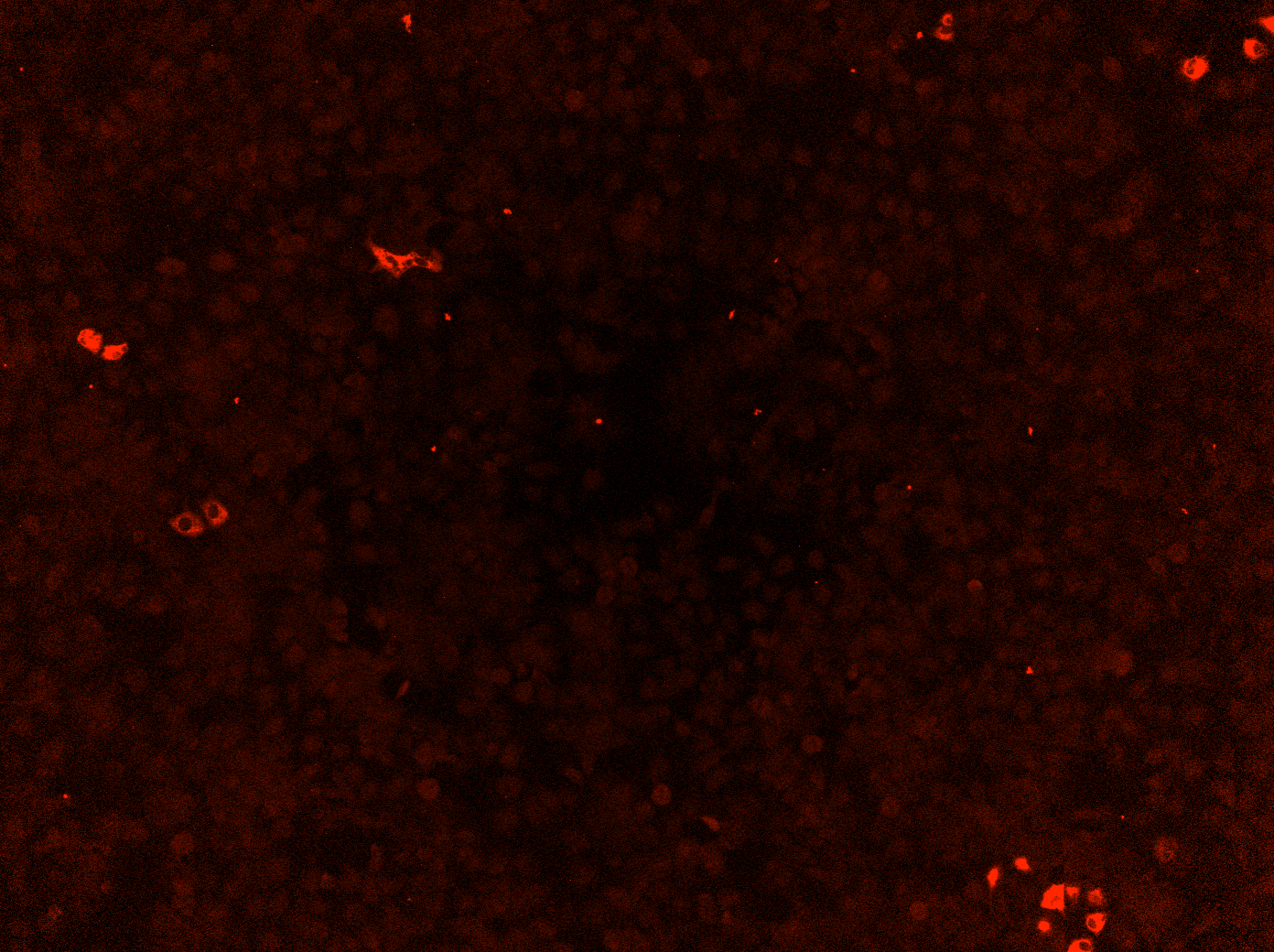

Supplement: Figure 7—figure supplement 1—source data 1. [file elife-52555-fig7-figsupp1-data1.zip › SD-figureS9/Steve G images/JK332-488/SteveG_F9_1.tif]

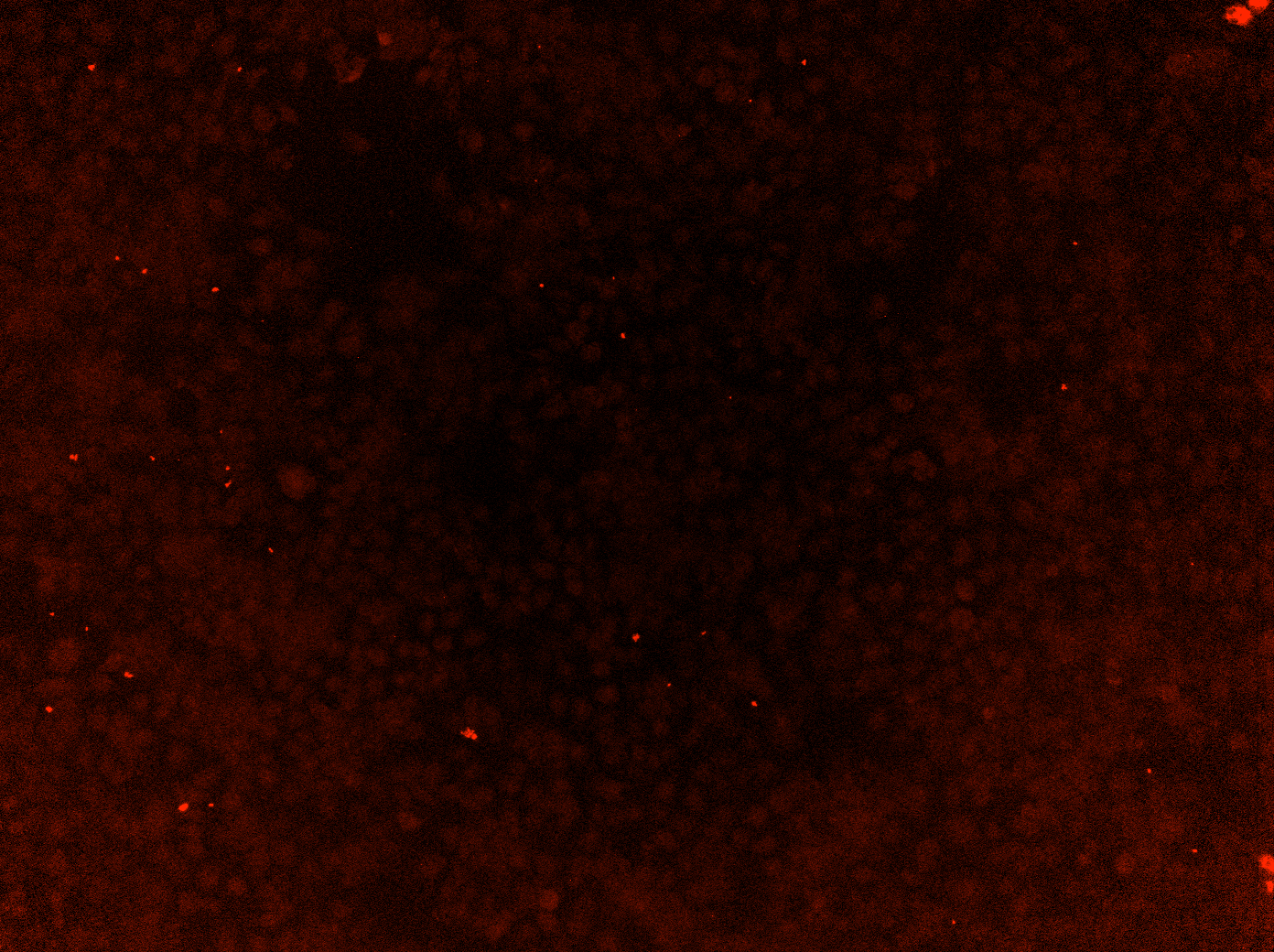

Supplement: Figure 7—figure supplement 1—source data 1. [file elife-52555-fig7-figsupp1-data1.zip › SD-figureS9/Steve G images/JK332/SteveG_G10_1.tif]

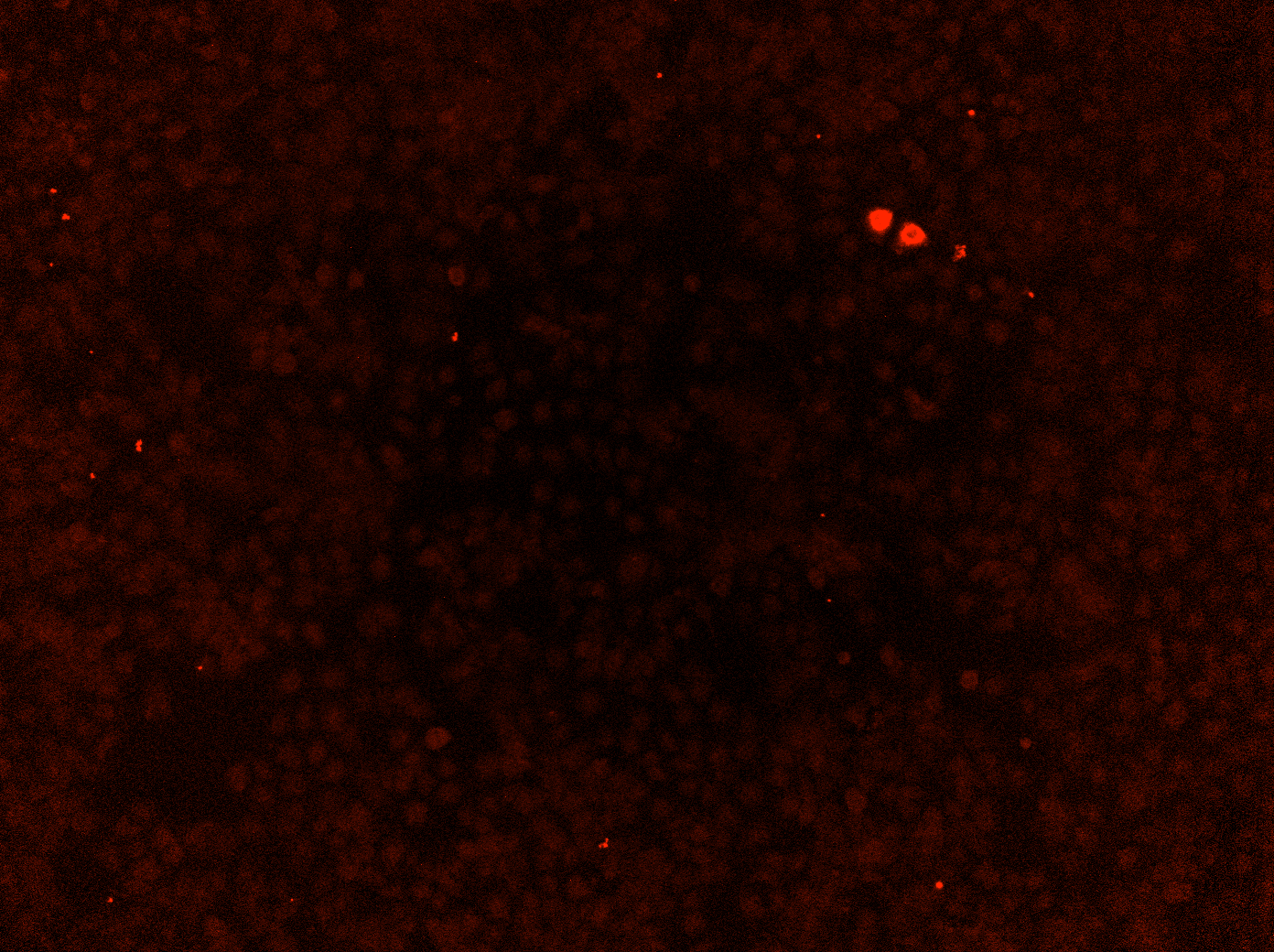

Supplement: Figure 7—figure supplement 1—source data 1. [file elife-52555-fig7-figsupp1-data1.zip › SD-figureS9/Steve G images/JK332/SteveG_G11_1.tif]

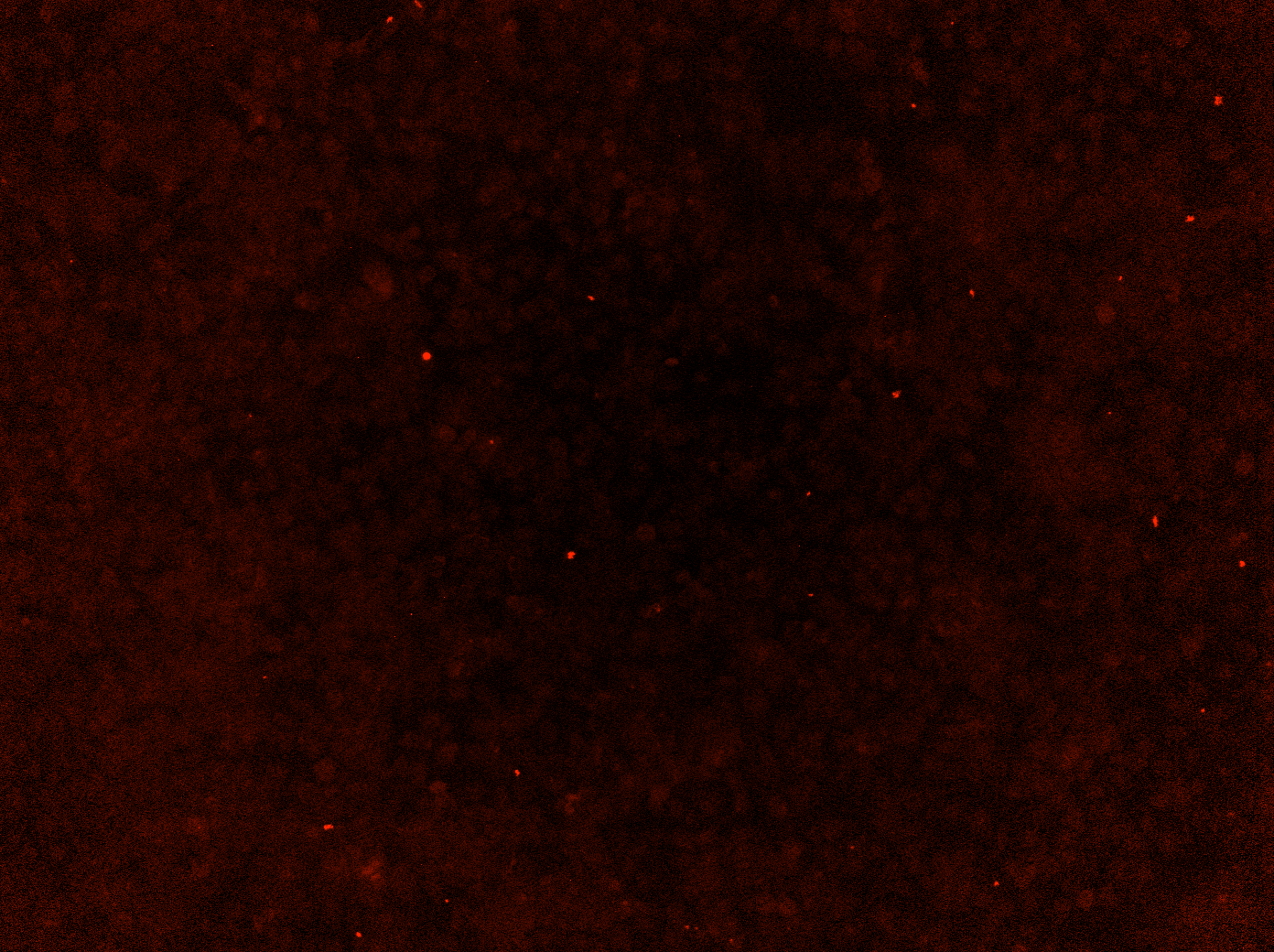

Supplement: Figure 7—figure supplement 1—source data 1. [file elife-52555-fig7-figsupp1-data1.zip › SD-figureS9/Steve G images/JK332/SteveG_G12_1.tif]

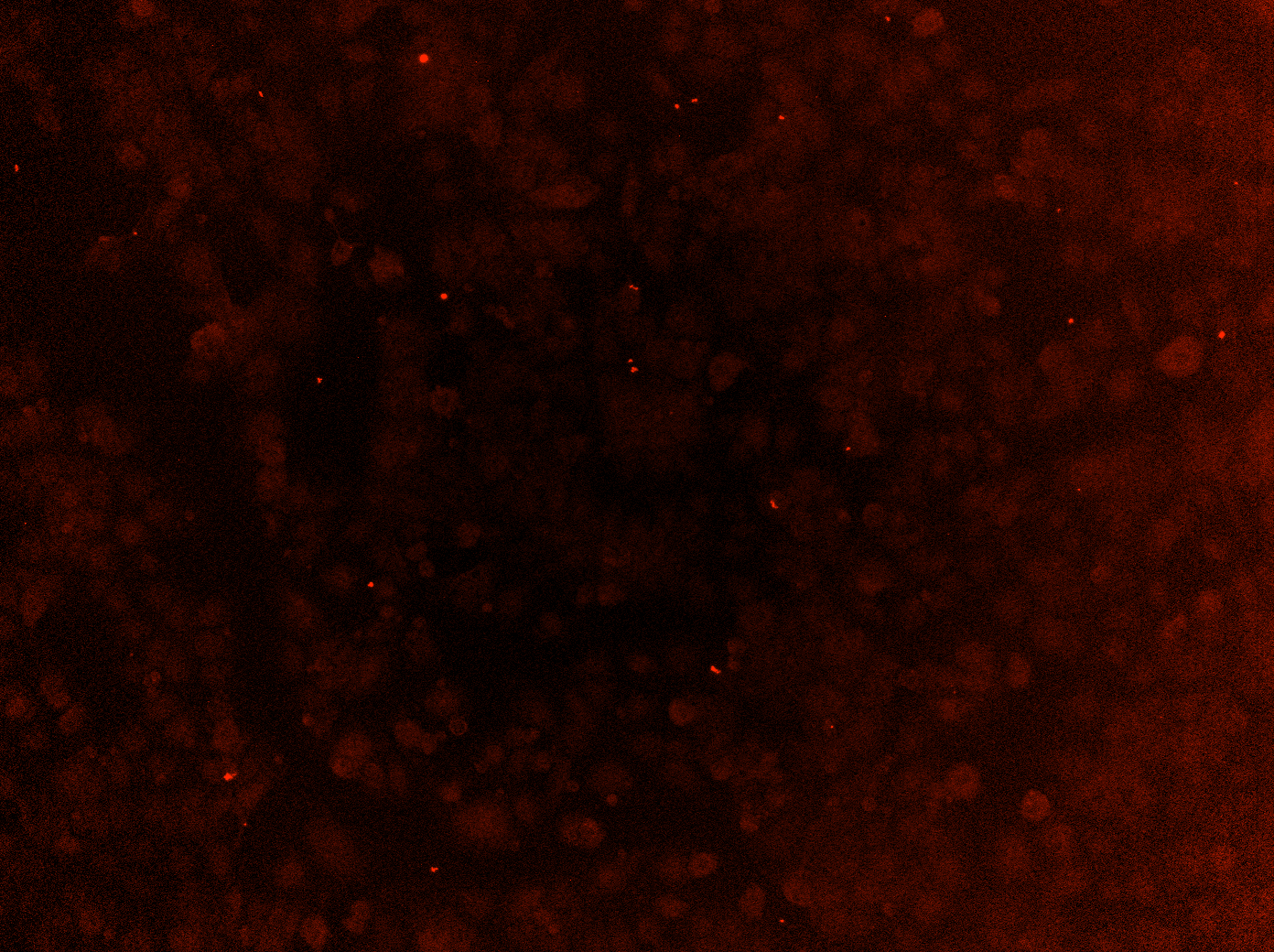

Supplement: Figure 7—figure supplement 1—source data 1. [file elife-52555-fig7-figsupp1-data1.zip › SD-figureS9/Steve G images/JK332/SteveG_G1_1.tif]

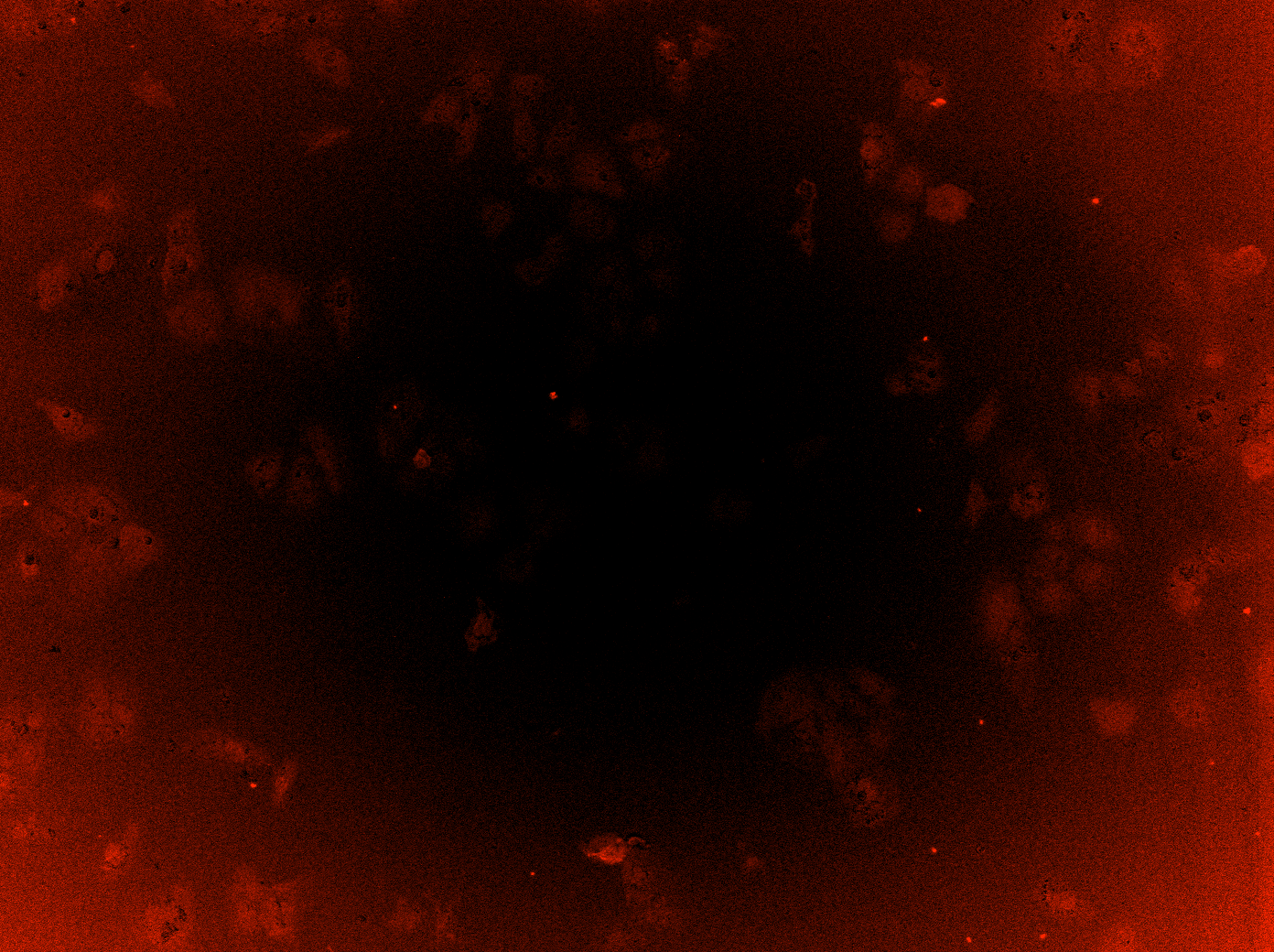

Supplement: Figure 7—figure supplement 1—source data 1. [file elife-52555-fig7-figsupp1-data1.zip › SD-figureS9/Steve G images/JK332/SteveG_G2_1.tif]

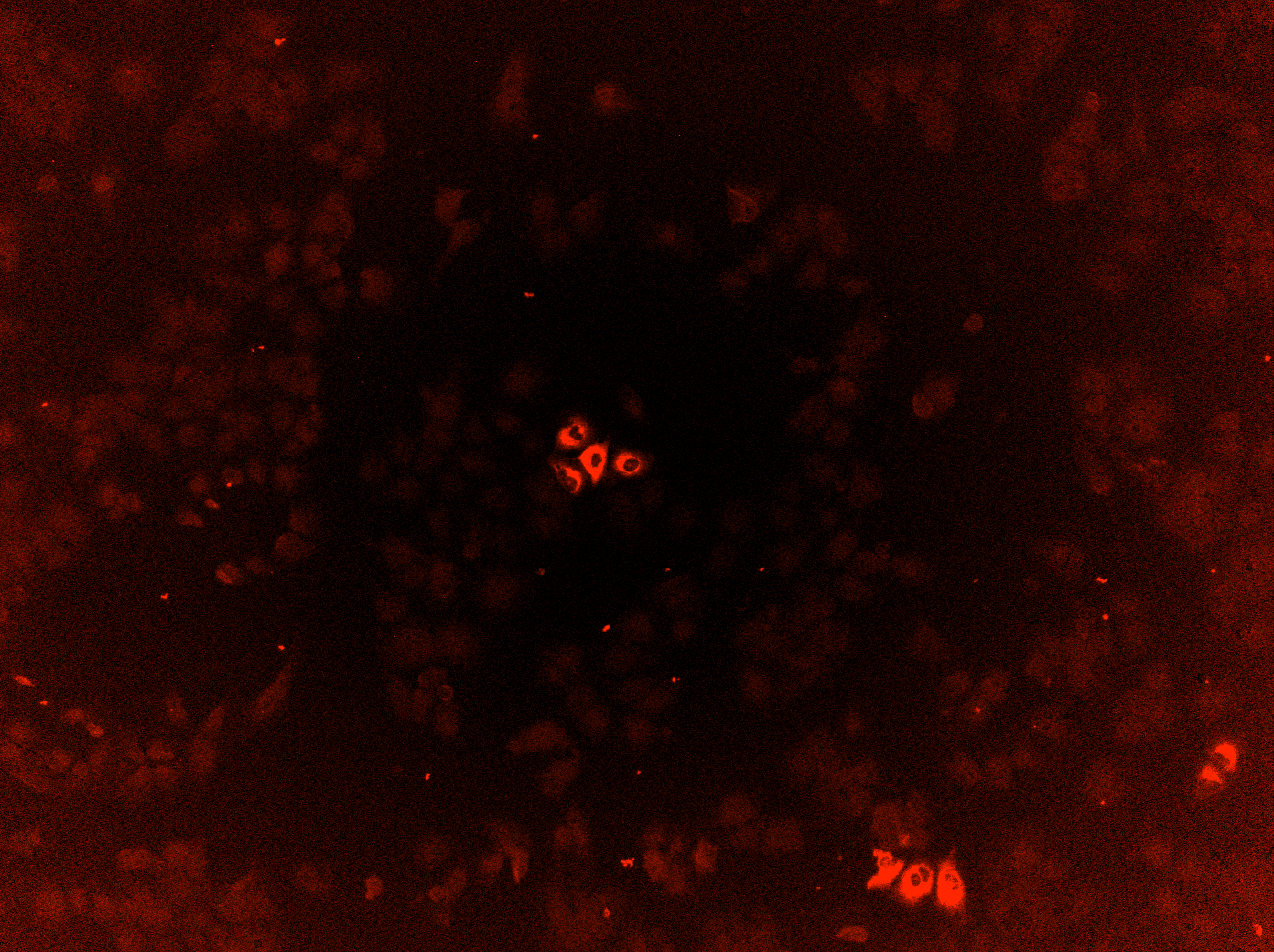

Supplement: Figure 7—figure supplement 1—source data 1. [file elife-52555-fig7-figsupp1-data1.zip › SD-figureS9/Steve G images/JK332/SteveG_G3_1.tif]

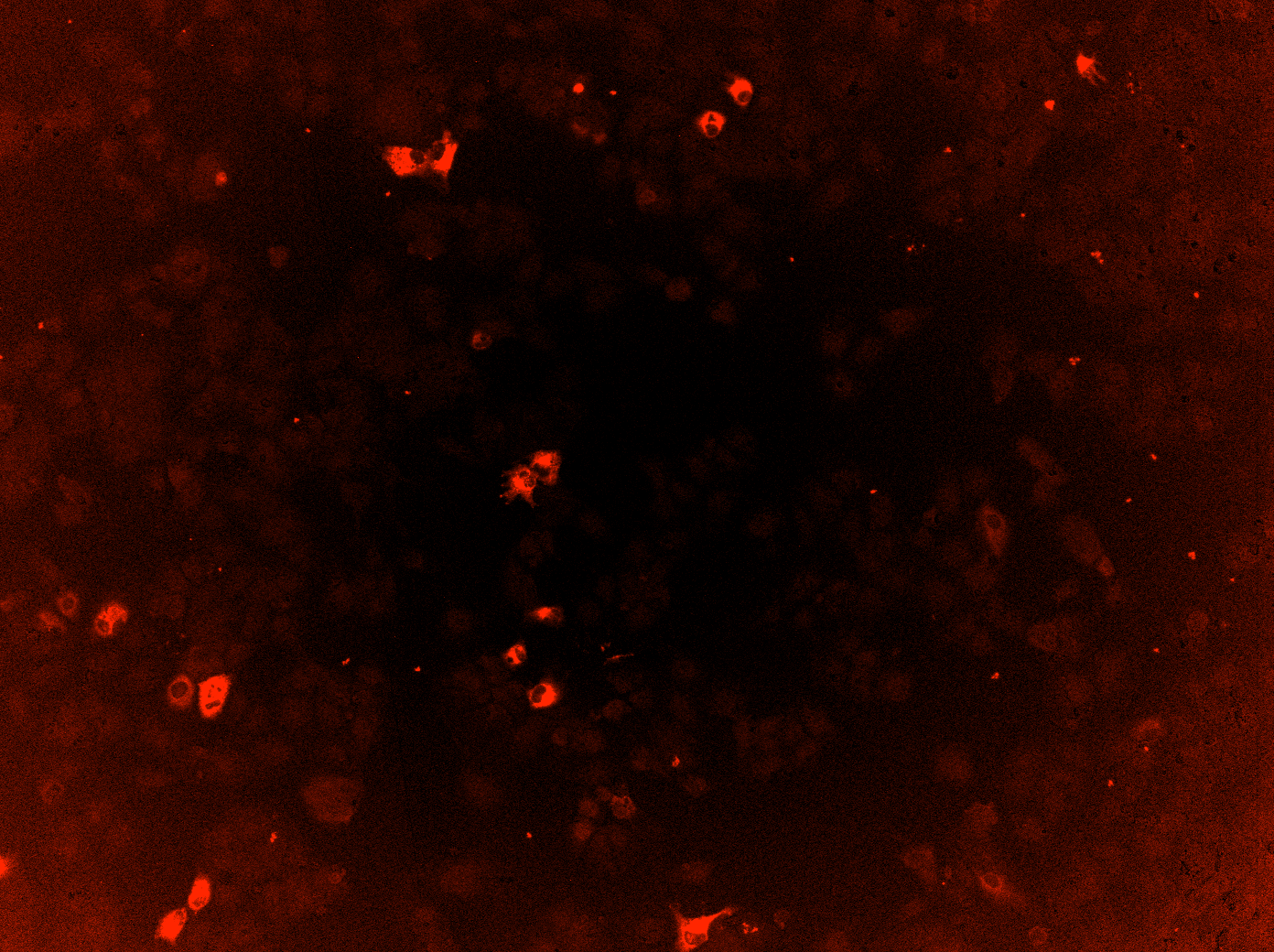

Supplement: Figure 7—figure supplement 1—source data 1. [file elife-52555-fig7-figsupp1-data1.zip › SD-figureS9/Steve G images/JK332/SteveG_G4_1.tif]

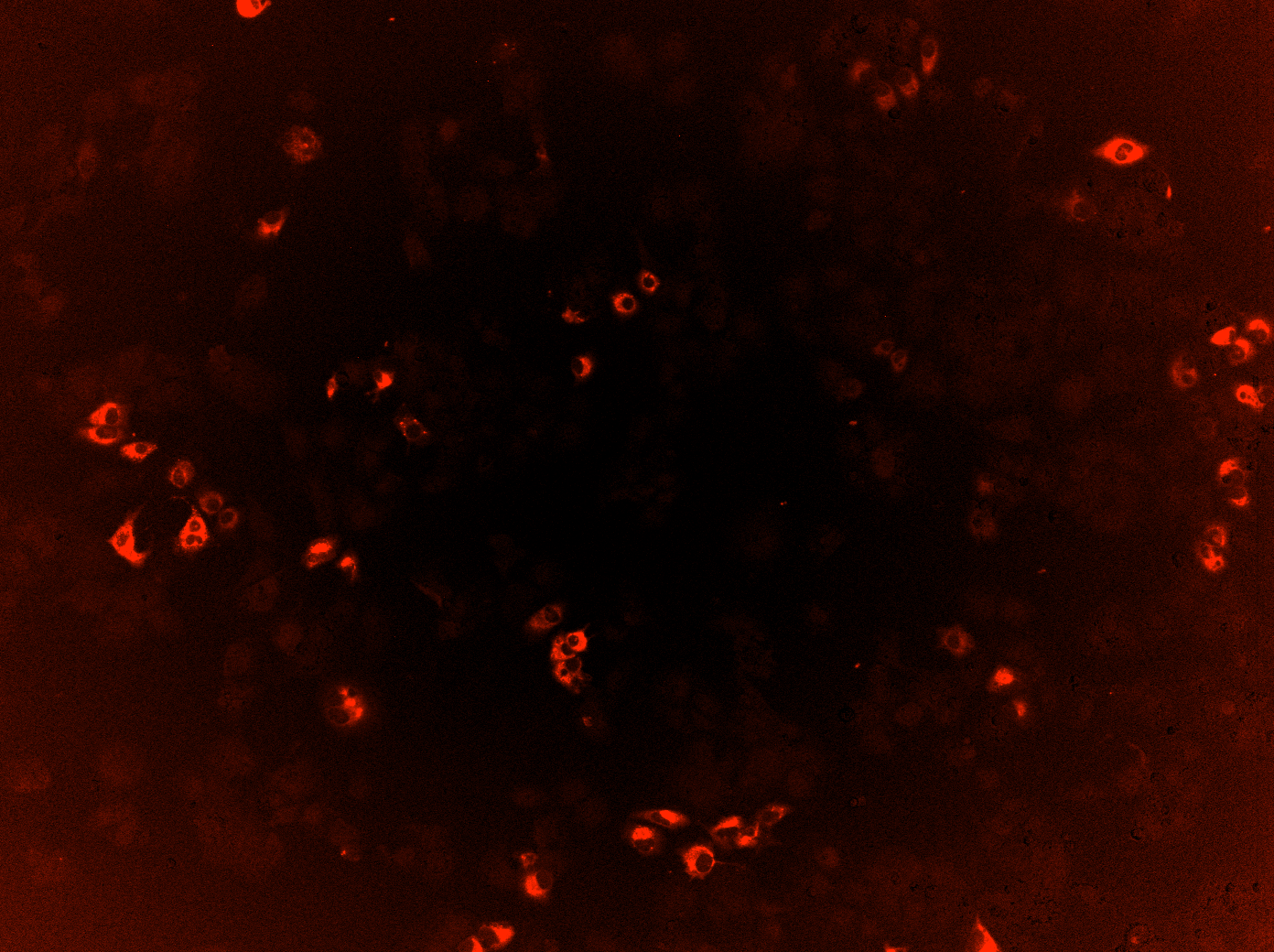

Supplement: Figure 7—figure supplement 1—source data 1. [file elife-52555-fig7-figsupp1-data1.zip › SD-figureS9/Steve G images/JK332/SteveG_G5_1.tif]

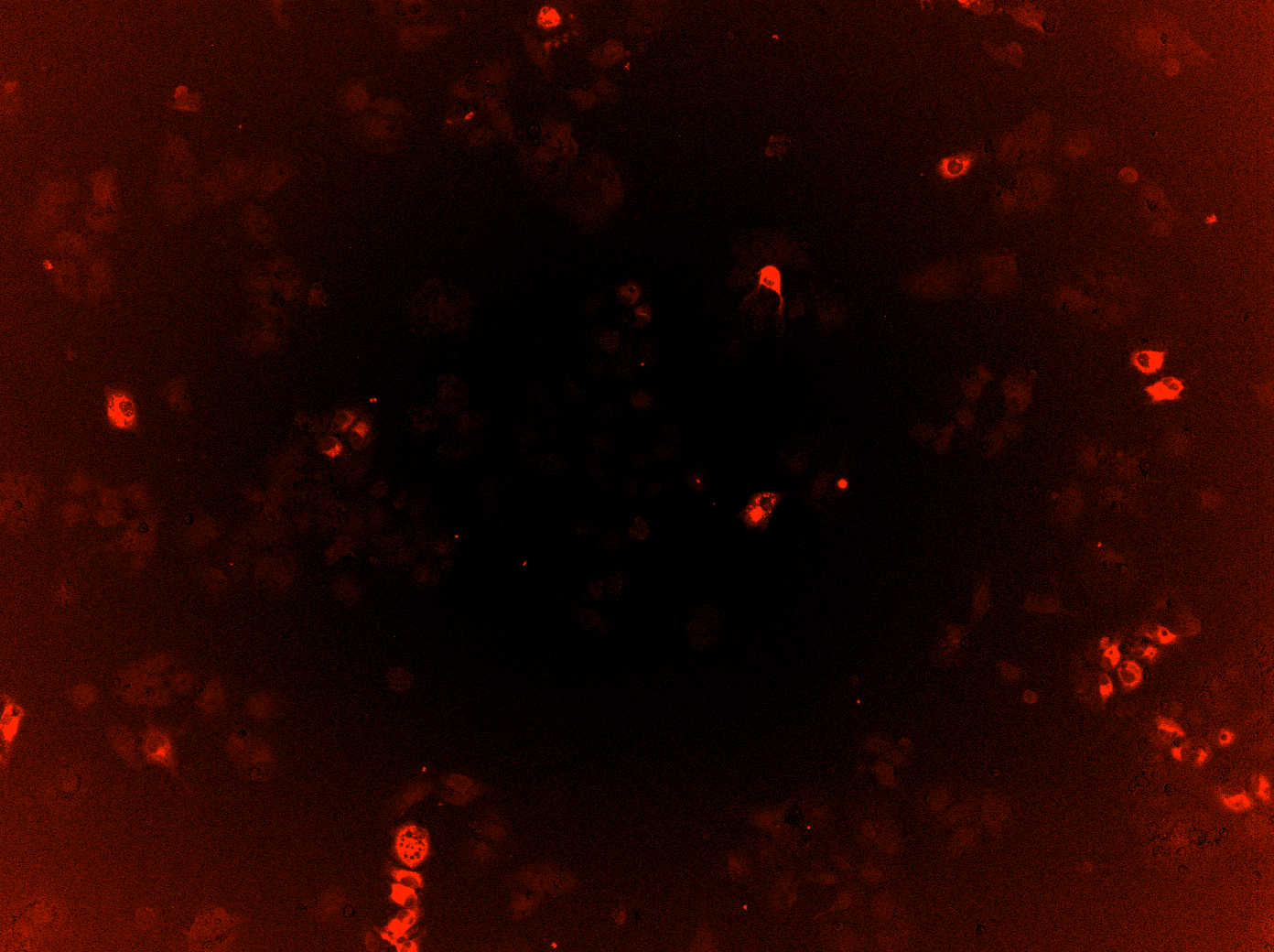

Supplement: Figure 7—figure supplement 1—source data 1. [file elife-52555-fig7-figsupp1-data1.zip › SD-figureS9/Steve G images/JK332/SteveG_G6_1.tif]

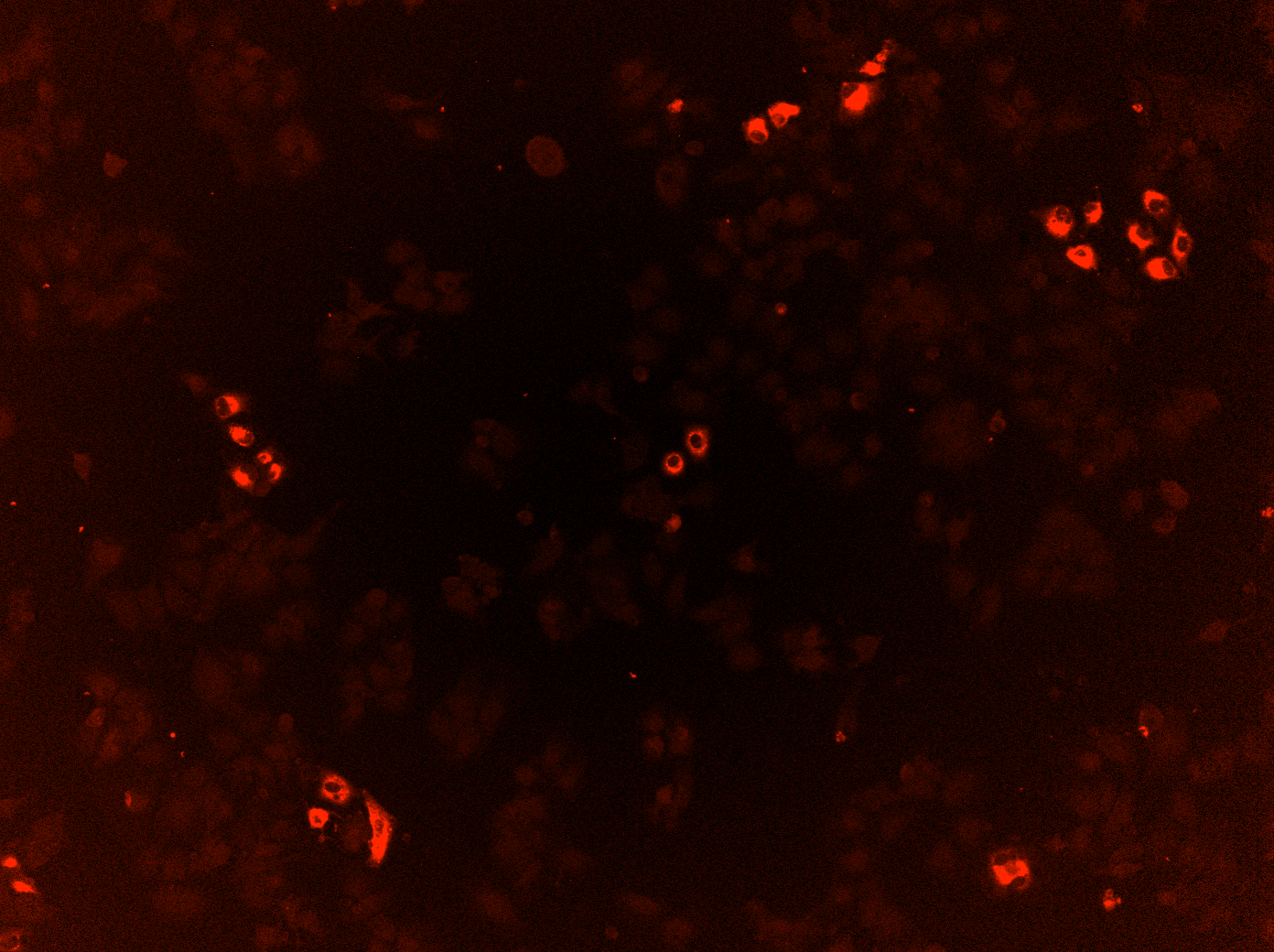

Supplement: Figure 7—figure supplement 1—source data 1. [file elife-52555-fig7-figsupp1-data1.zip › SD-figureS9/Steve G images/JK332/SteveG_G7_1.tif]

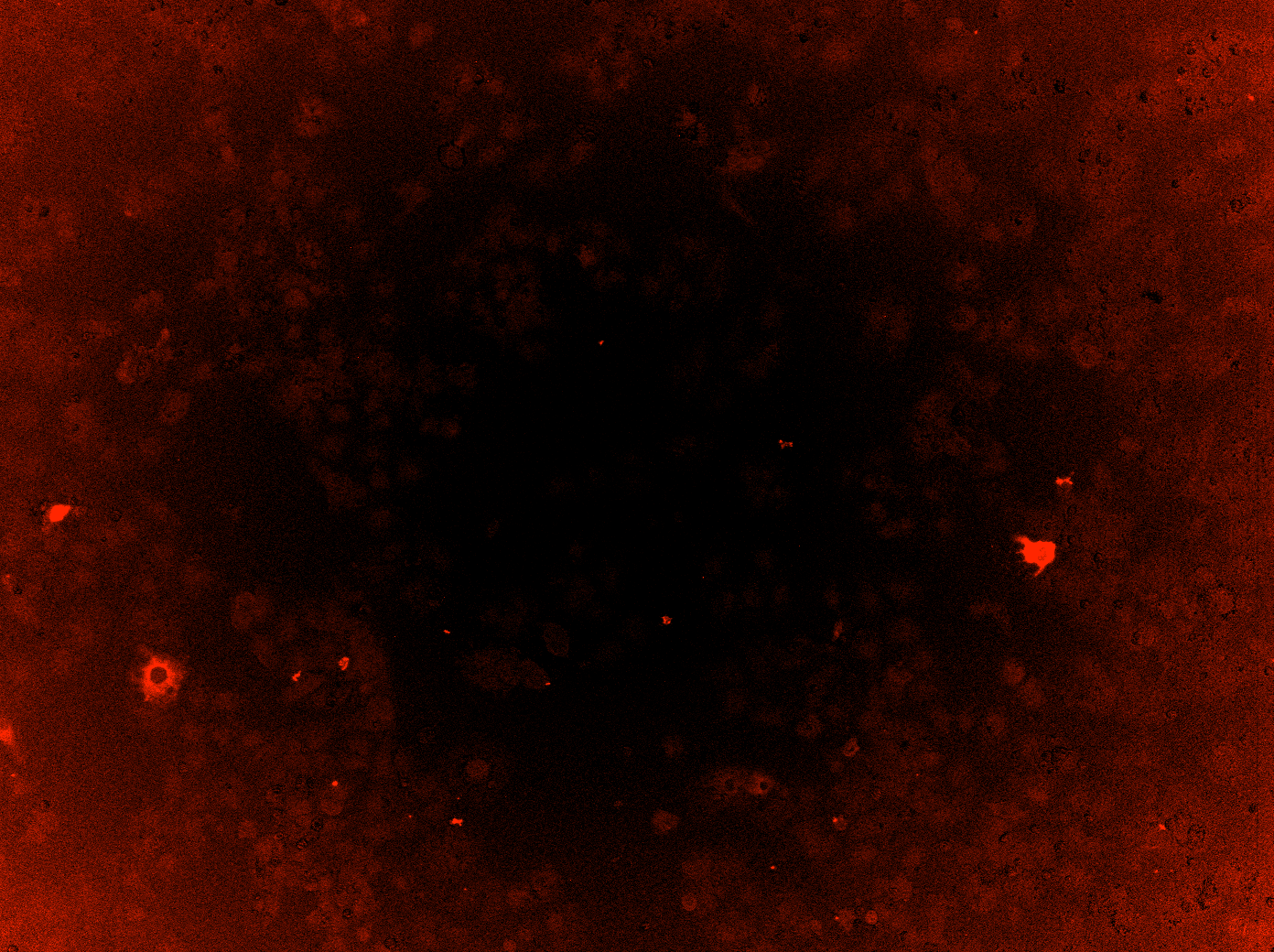

Supplement: Figure 7—figure supplement 1—source data 1. [file elife-52555-fig7-figsupp1-data1.zip › SD-figureS9/Steve G images/JK332/SteveG_G8_1.tif]

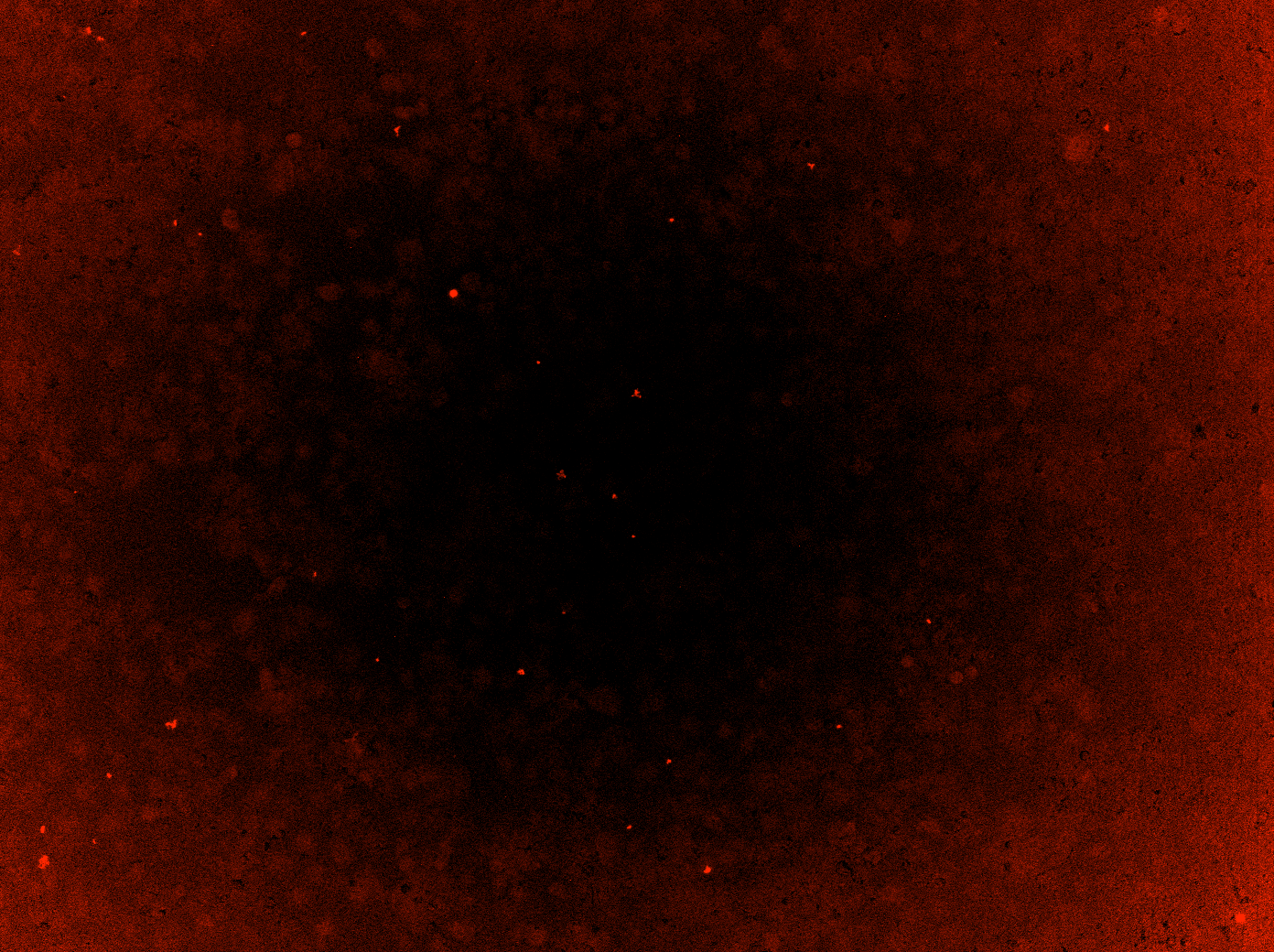

Supplement: Figure 7—figure supplement 1—source data 1. [file elife-52555-fig7-figsupp1-data1.zip › SD-figureS9/Steve G images/JK332/SteveG_G9_1.tif]

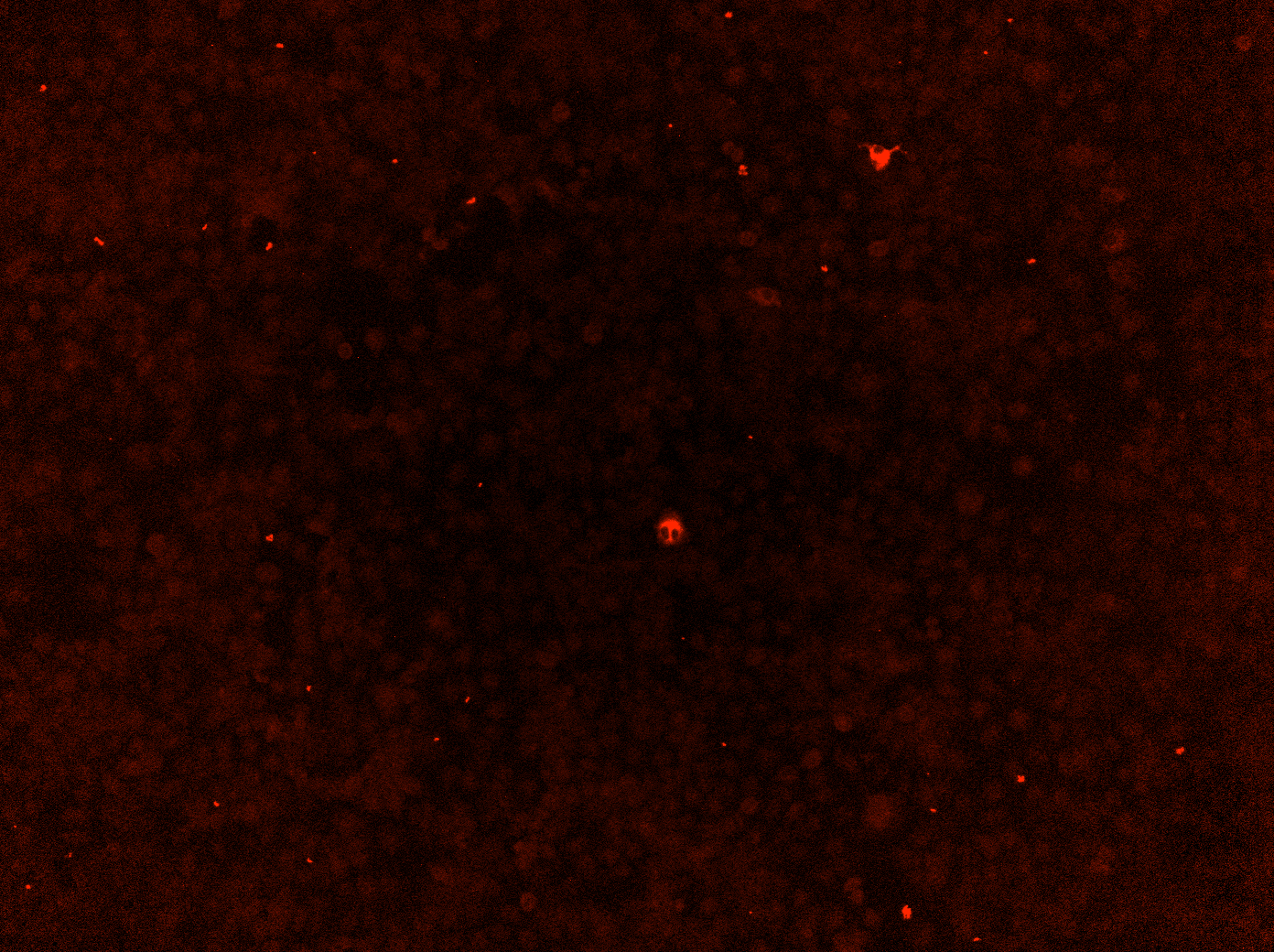

Supplement: Figure 7—figure supplement 1—source data 1. [file elife-52555-fig7-figsupp1-data1.zip › SD-figureS9/Steve G images/R21/SteveG_E10_1.tif]

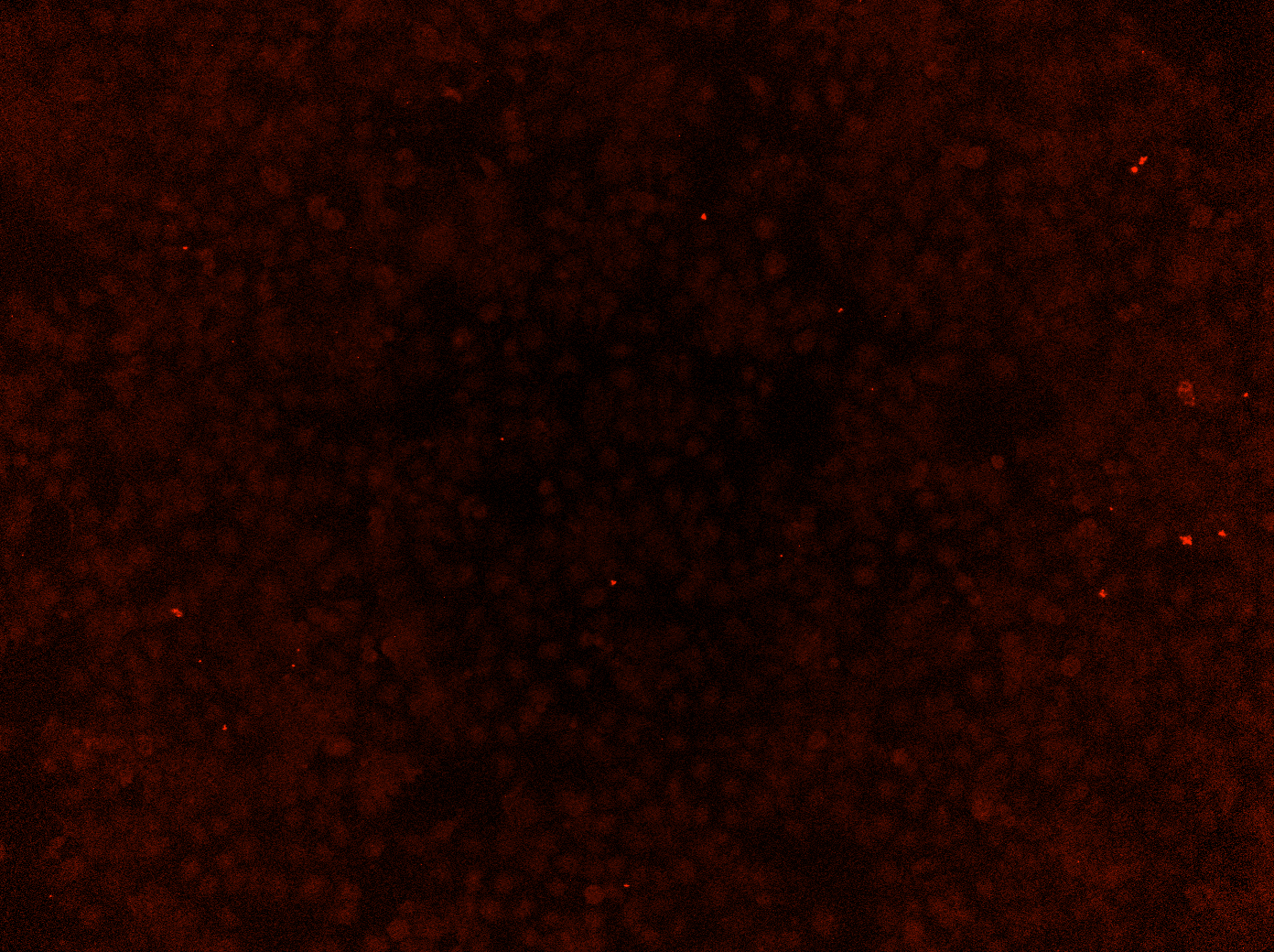

Supplement: Figure 7—figure supplement 1—source data 1. [file elife-52555-fig7-figsupp1-data1.zip › SD-figureS9/Steve G images/R21/SteveG_E11_1.tif]

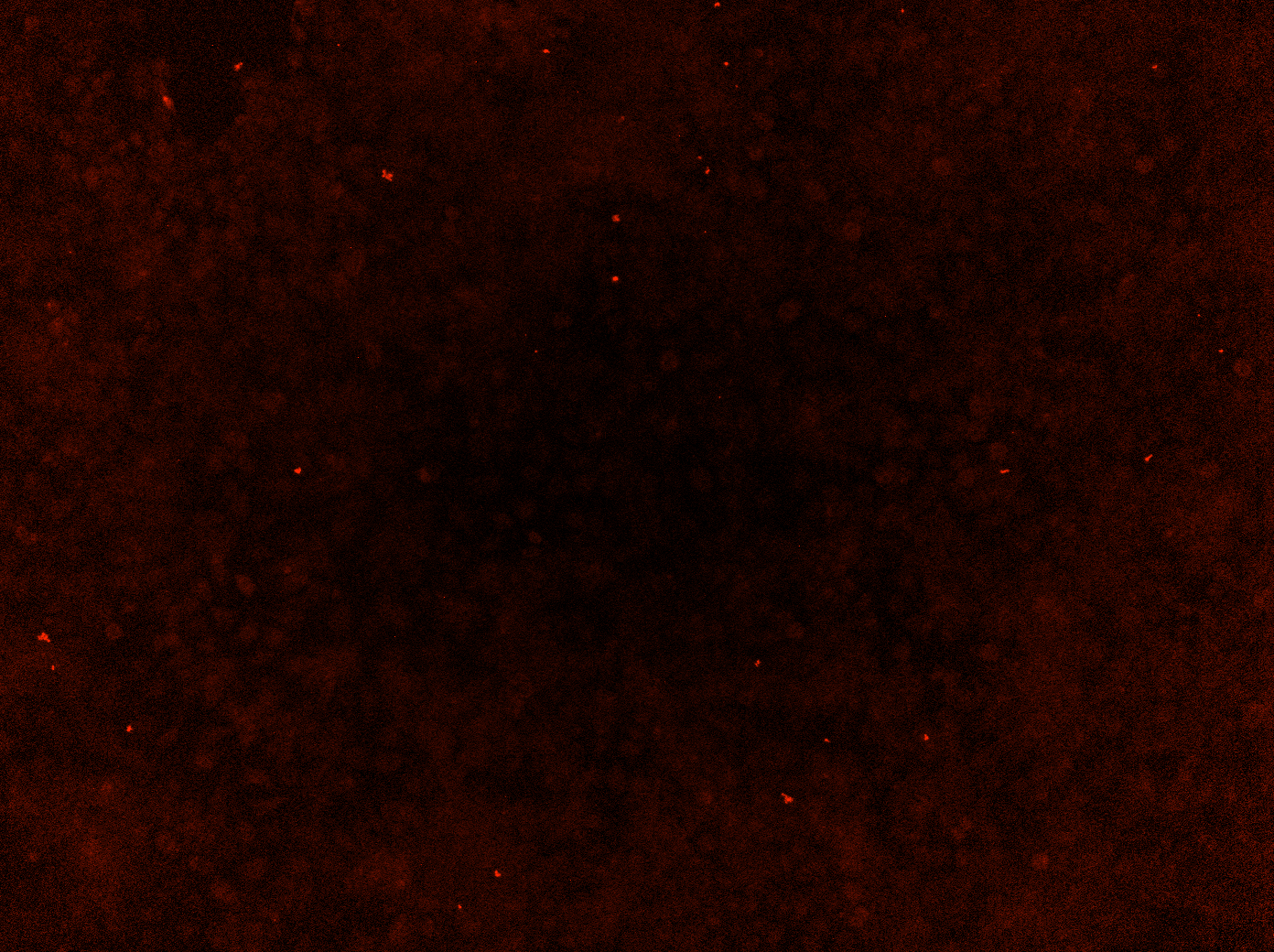

Supplement: Figure 7—figure supplement 1—source data 1. [file elife-52555-fig7-figsupp1-data1.zip › SD-figureS9/Steve G images/R21/SteveG_E12_1.tif]

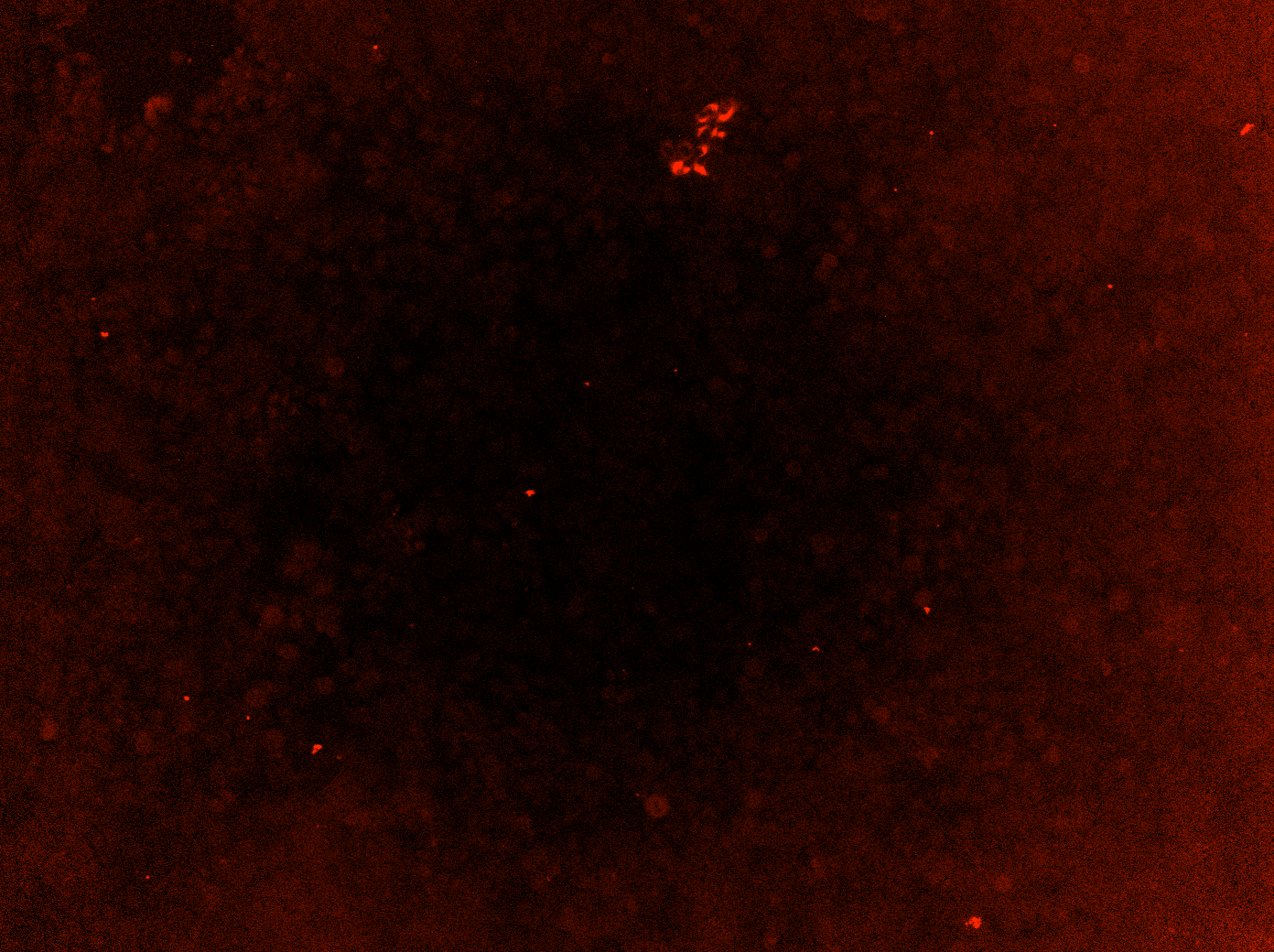

Supplement: Figure 7—figure supplement 1—source data 1. [file elife-52555-fig7-figsupp1-data1.zip › SD-figureS9/Steve G images/R21/SteveG_E1_1.tif]

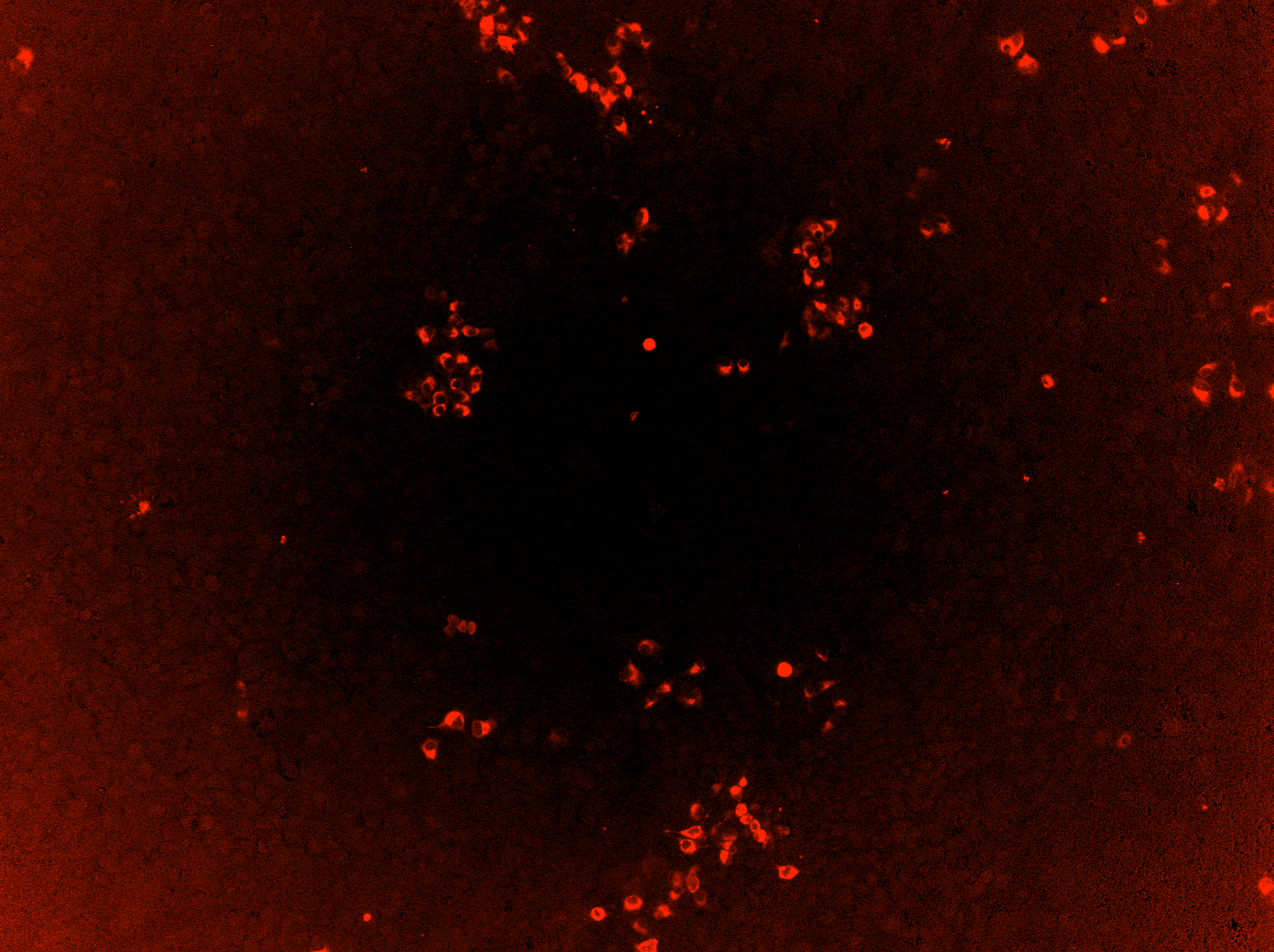

Supplement: Figure 7—figure supplement 1—source data 1. [file elife-52555-fig7-figsupp1-data1.zip › SD-figureS9/Steve G images/R21/SteveG_E2_1.tif]

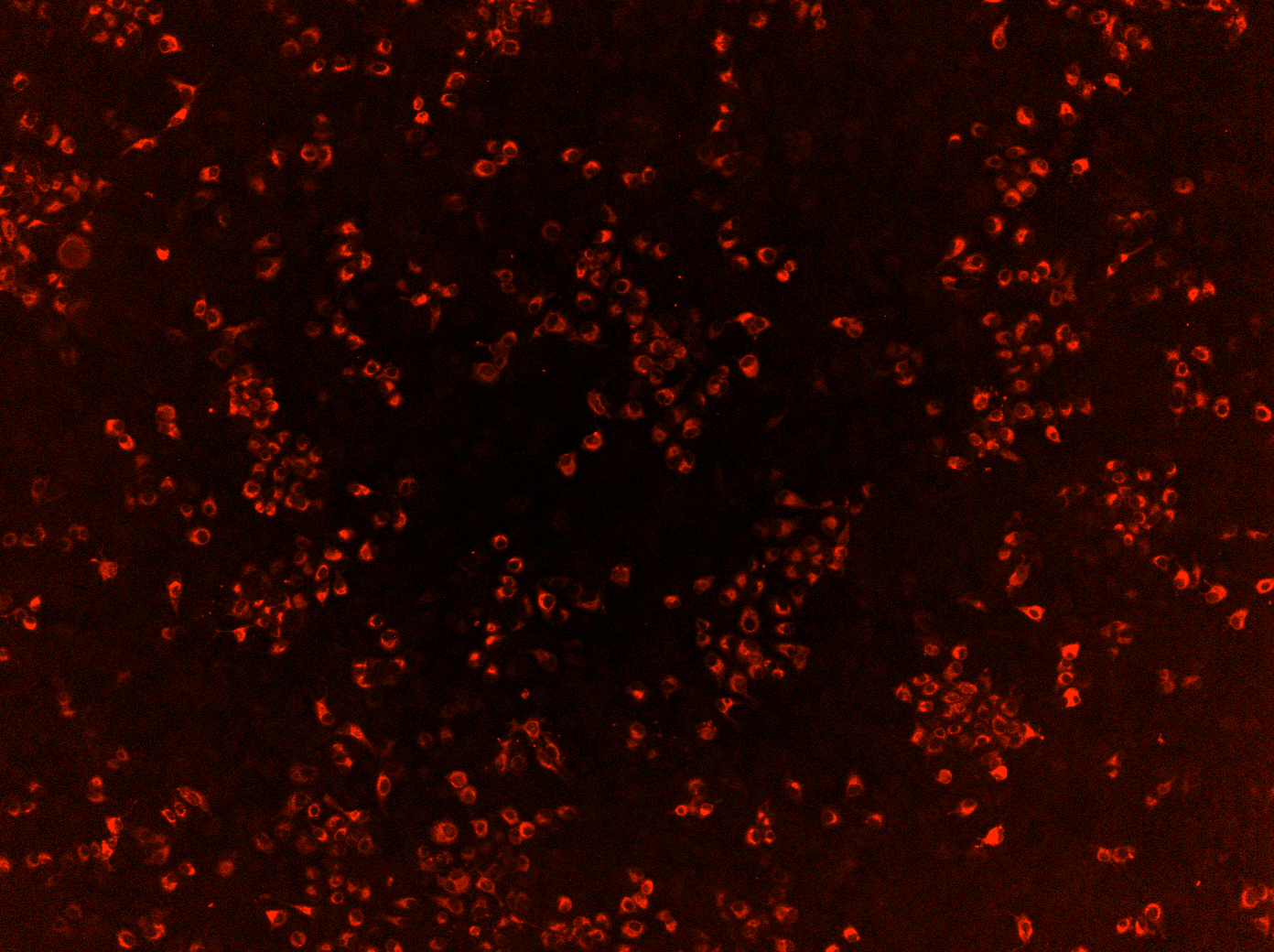

Supplement: Figure 7—figure supplement 1—source data 1. [file elife-52555-fig7-figsupp1-data1.zip › SD-figureS9/Steve G images/R21/SteveG_E3_1.tif]

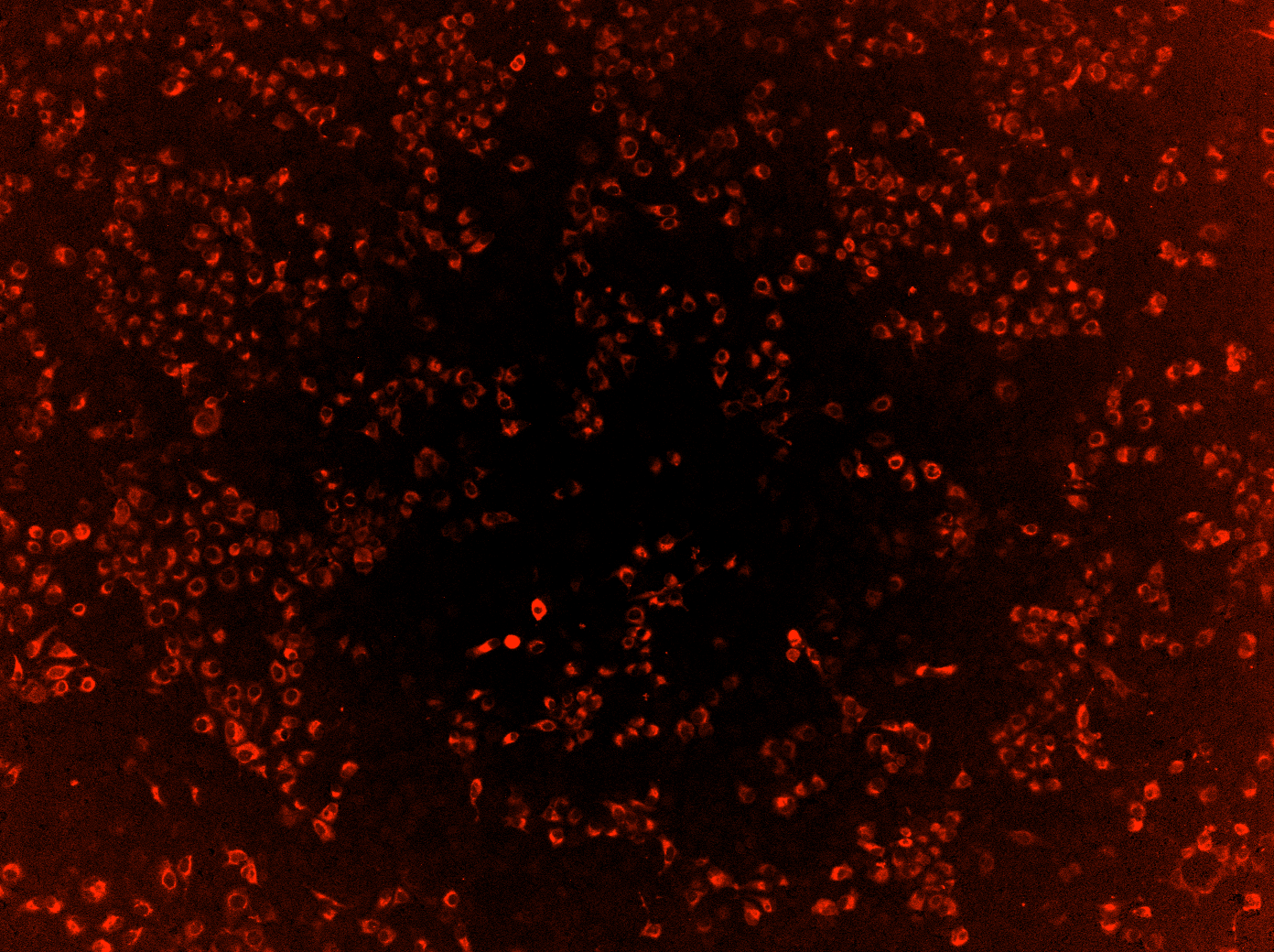

Supplement: Figure 7—figure supplement 1—source data 1. [file elife-52555-fig7-figsupp1-data1.zip › SD-figureS9/Steve G images/R21/SteveG_E4_1.tif]

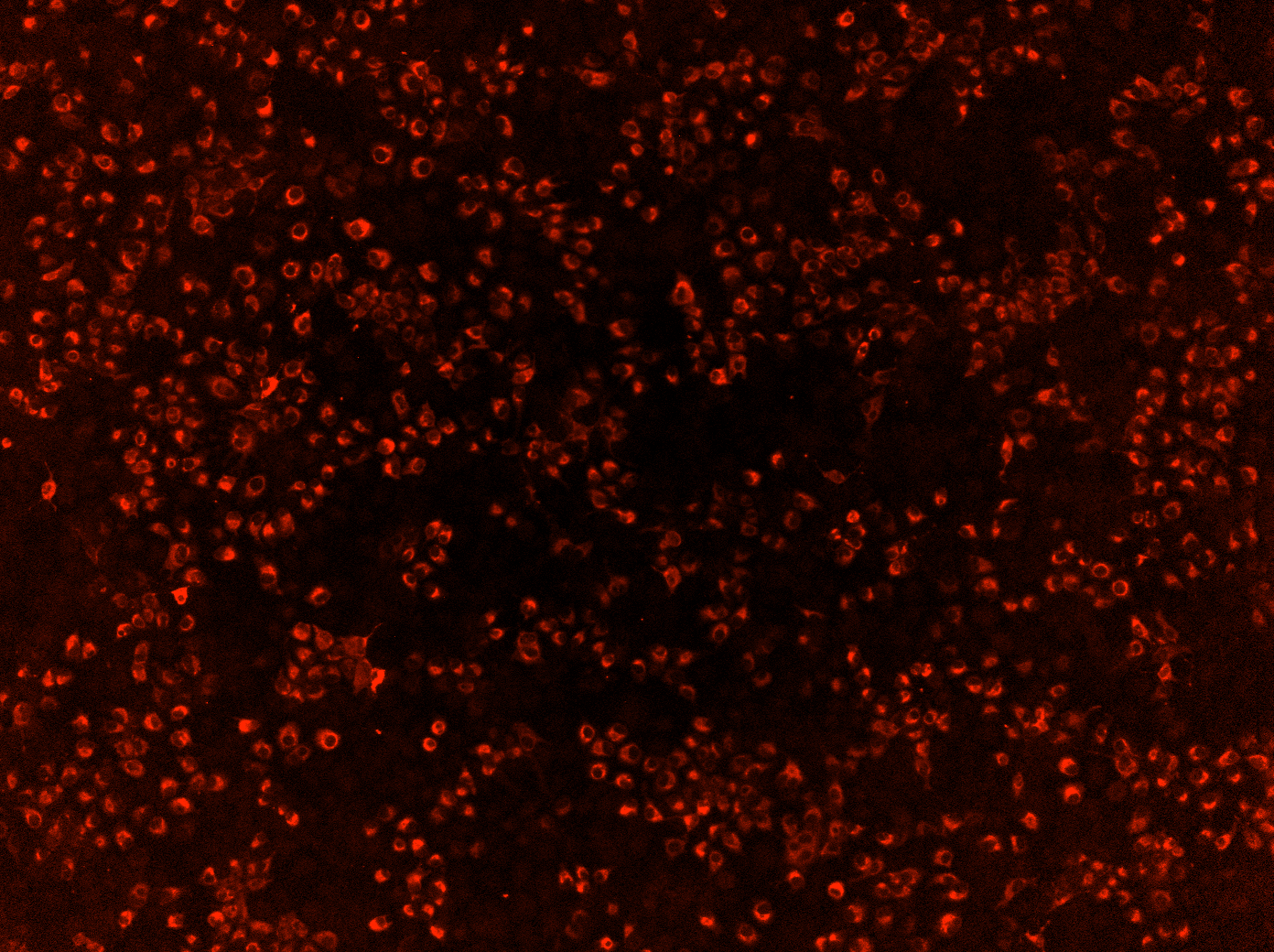

Supplement: Figure 7—figure supplement 1—source data 1. [file elife-52555-fig7-figsupp1-data1.zip › SD-figureS9/Steve G images/R21/SteveG_E5_1.tif]

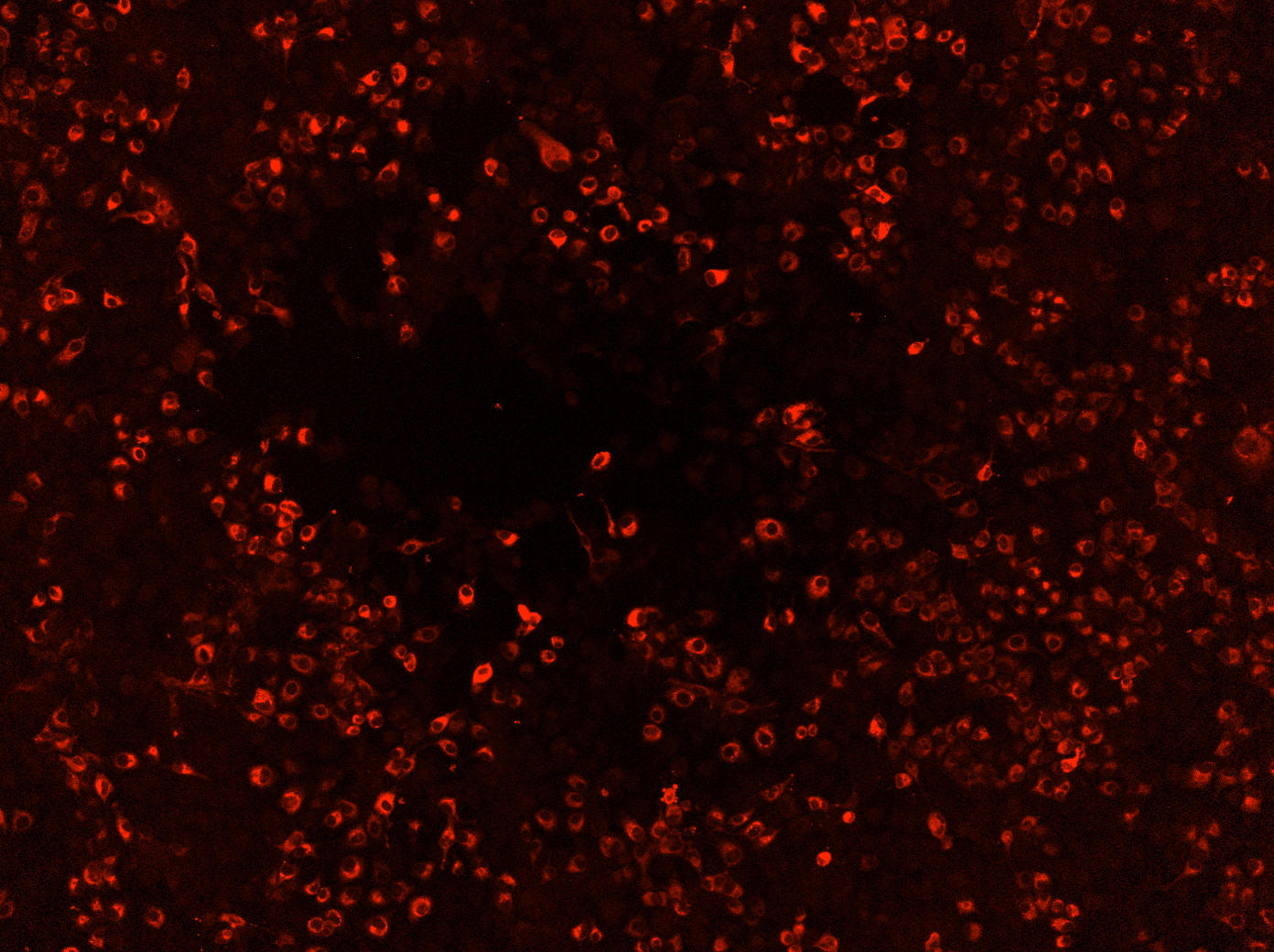

Supplement: Figure 7—figure supplement 1—source data 1. [file elife-52555-fig7-figsupp1-data1.zip › SD-figureS9/Steve G images/R21/SteveG_E6_1.tif]

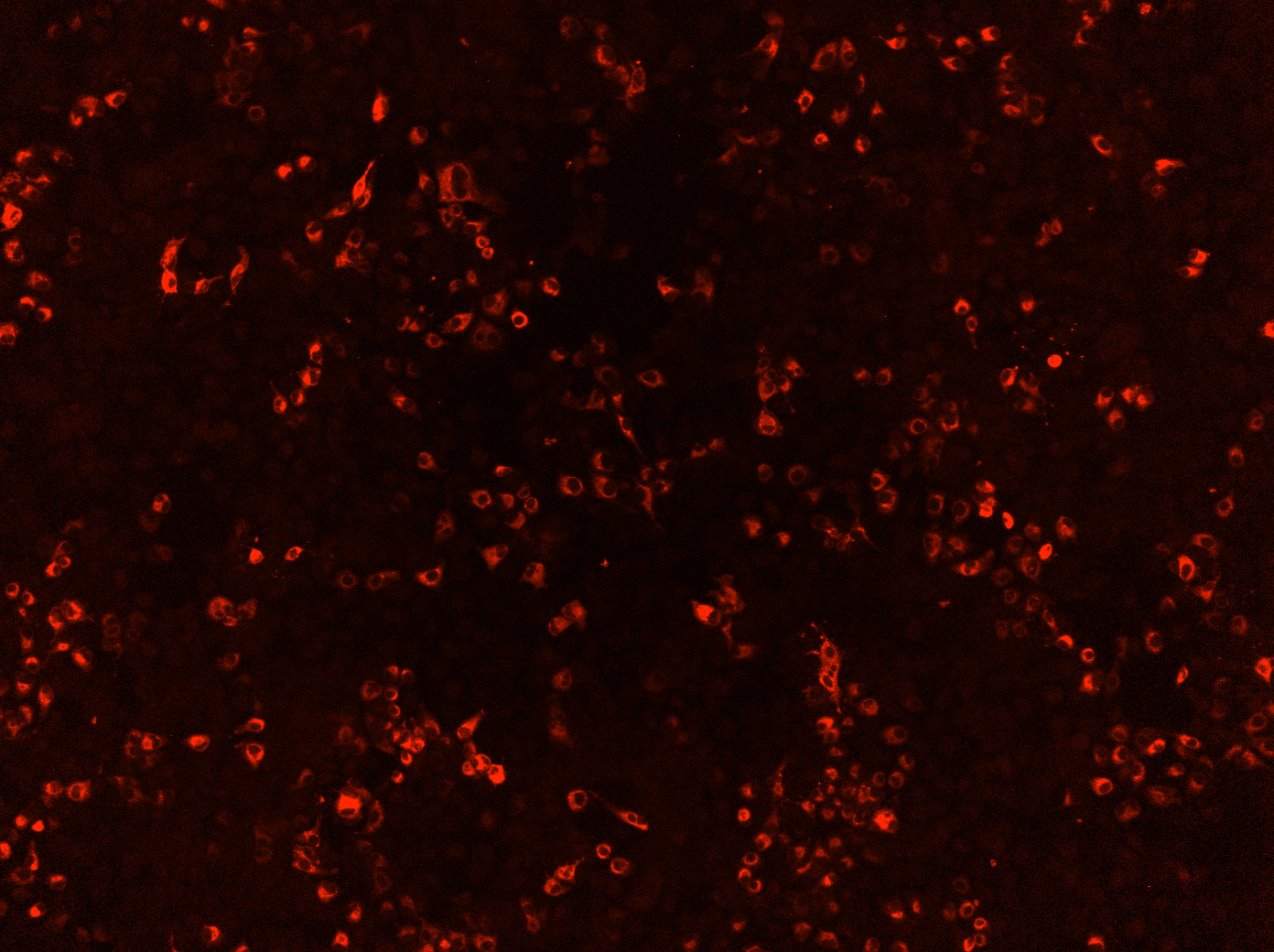

Supplement: Figure 7—figure supplement 1—source data 1. [file elife-52555-fig7-figsupp1-data1.zip › SD-figureS9/Steve G images/R21/SteveG_E7_1.tif]

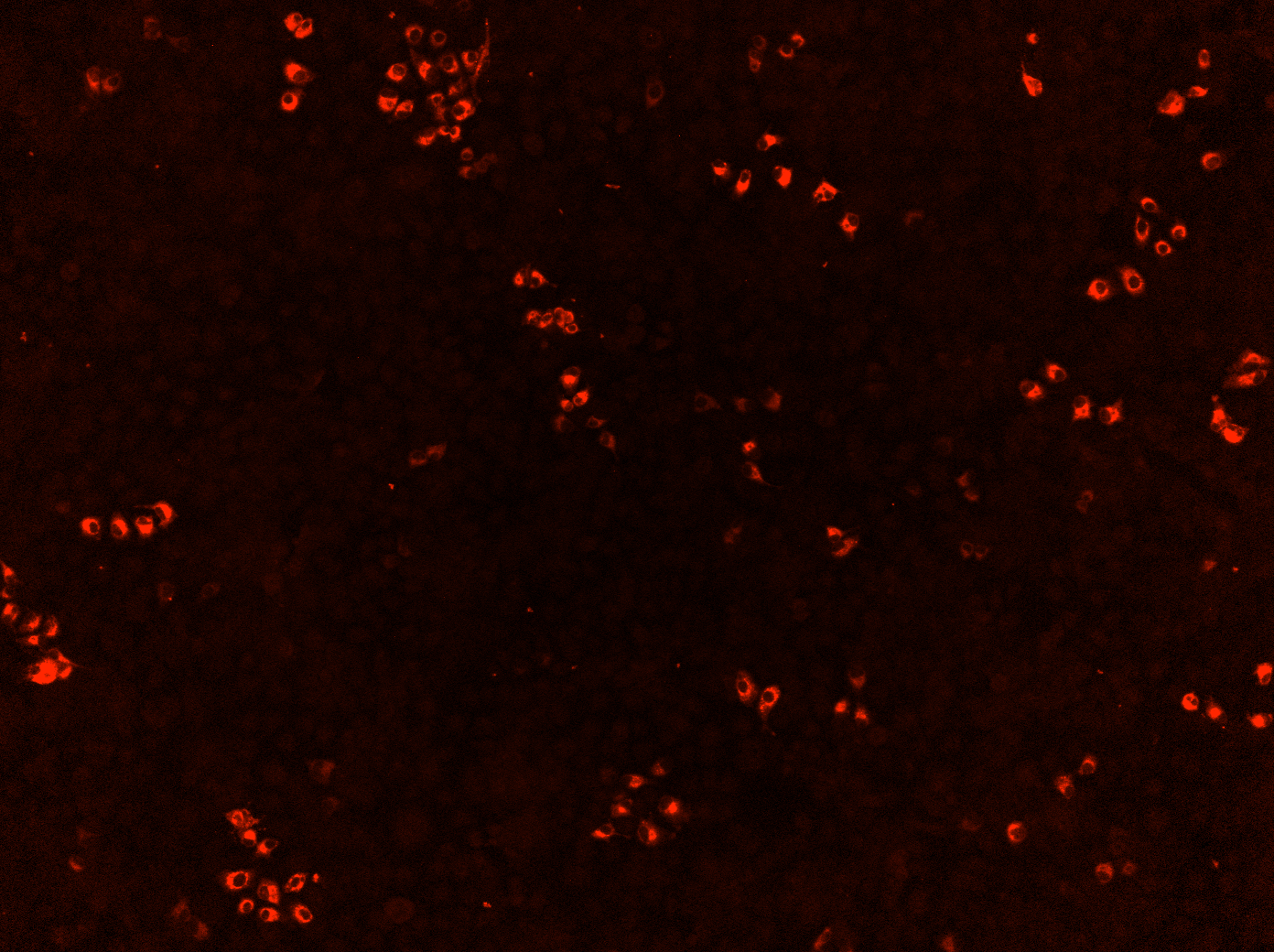

Supplement: Figure 7—figure supplement 1—source data 1. [file elife-52555-fig7-figsupp1-data1.zip › SD-figureS9/Steve G images/R21/SteveG_E8_1.tif]

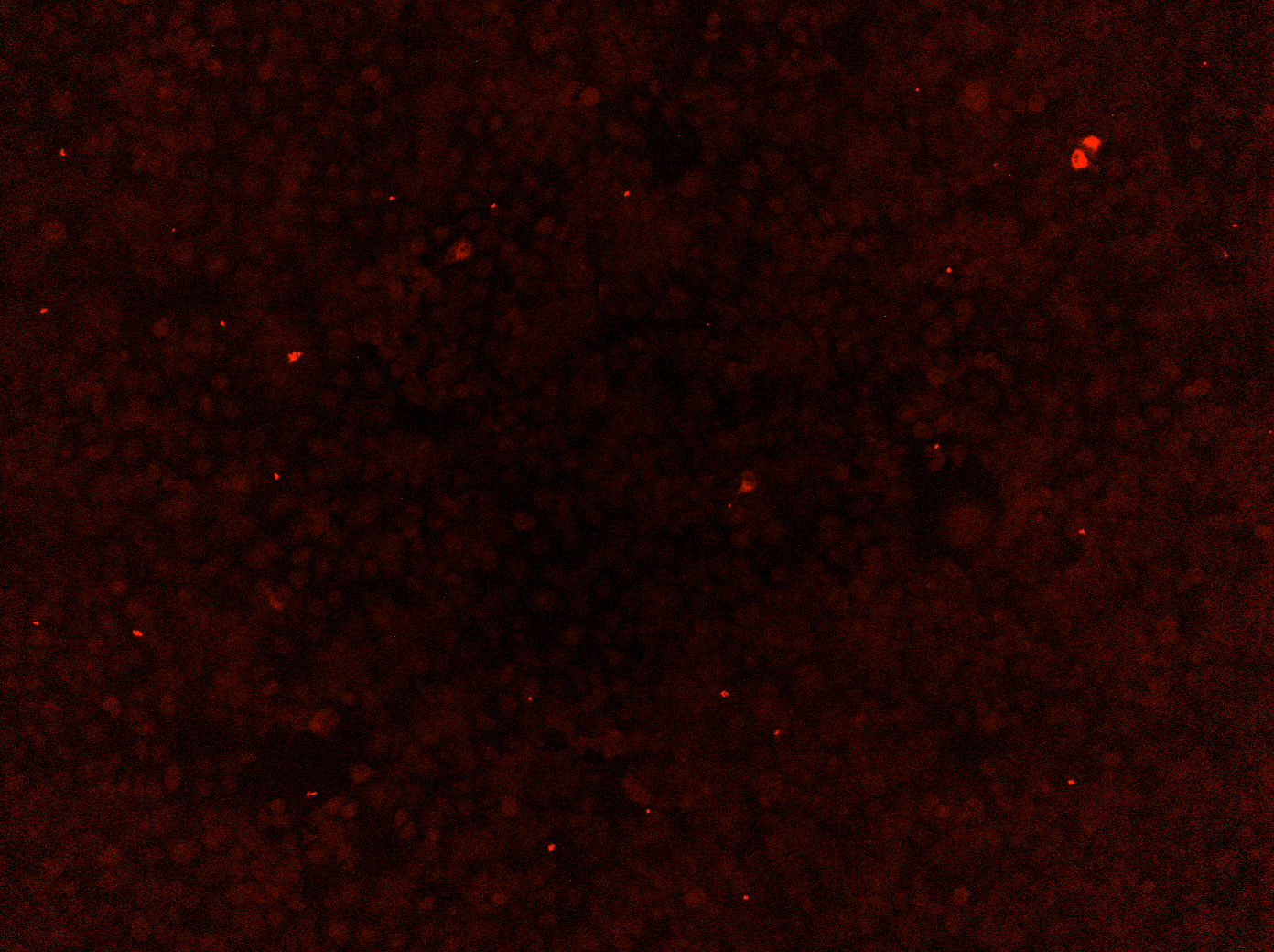

Supplement: Figure 7—figure supplement 1—source data 1. [file elife-52555-fig7-figsupp1-data1.zip › SD-figureS9/Steve G images/R21/SteveG_E9_1.tif]

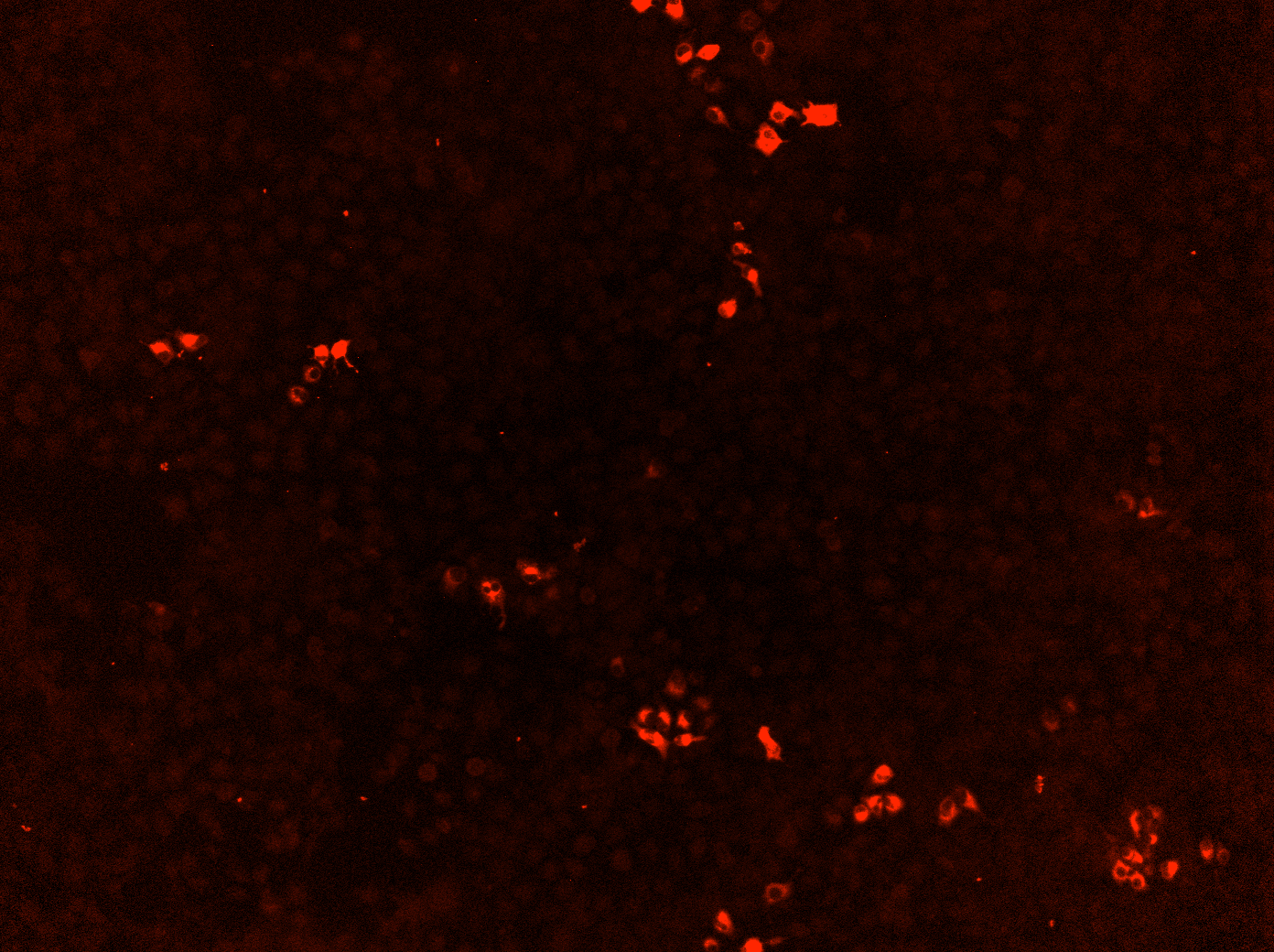

Supplement: Figure 7—figure supplement 1—source data 1. [file elife-52555-fig7-figsupp1-data1.zip › SD-figureS9/Steve G images/Wild type/SteveG_A10_1.tif]

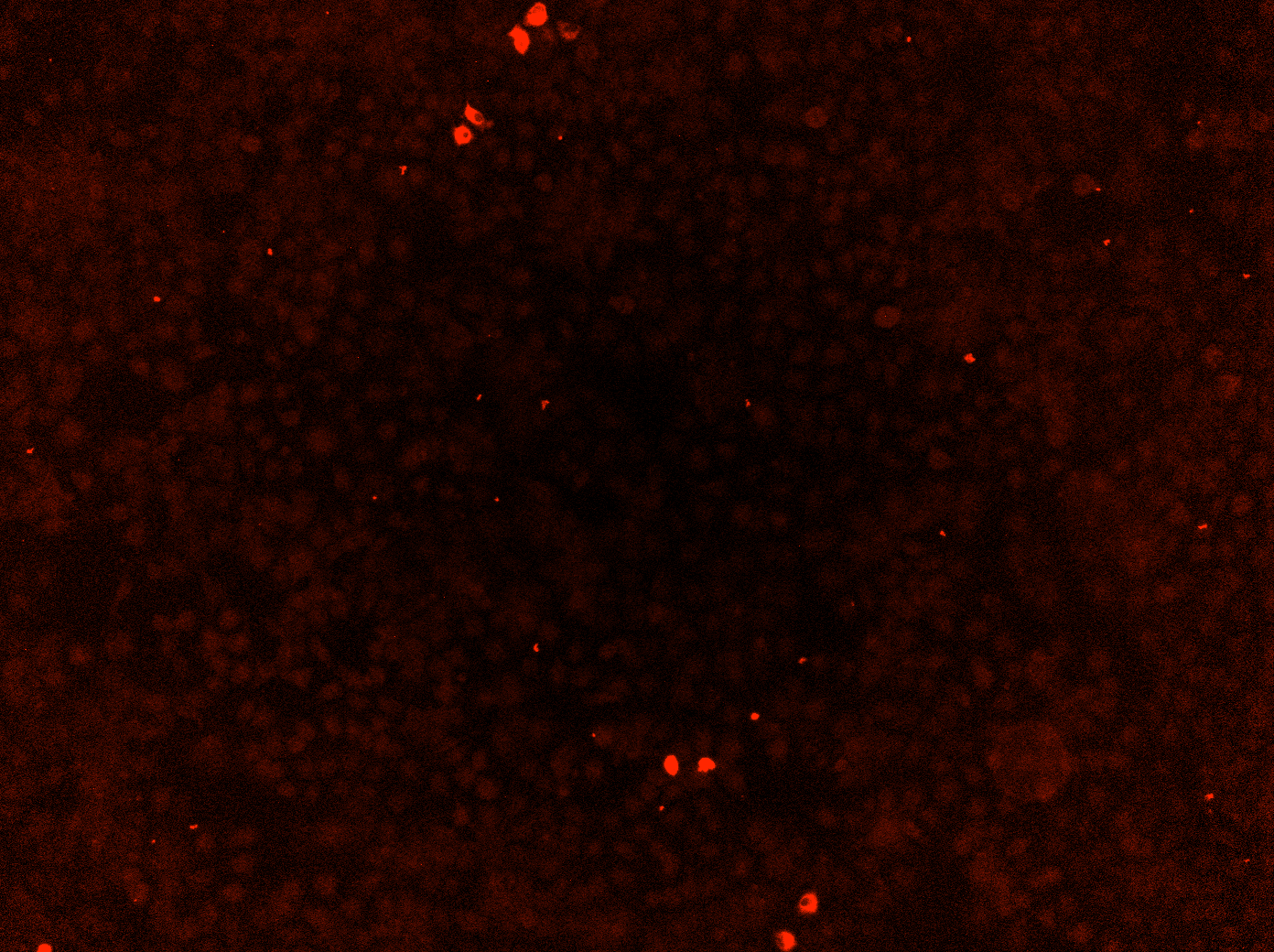

Supplement: Figure 7—figure supplement 1—source data 1. [file elife-52555-fig7-figsupp1-data1.zip › SD-figureS9/Steve G images/Wild type/SteveG_A11_1.tif]

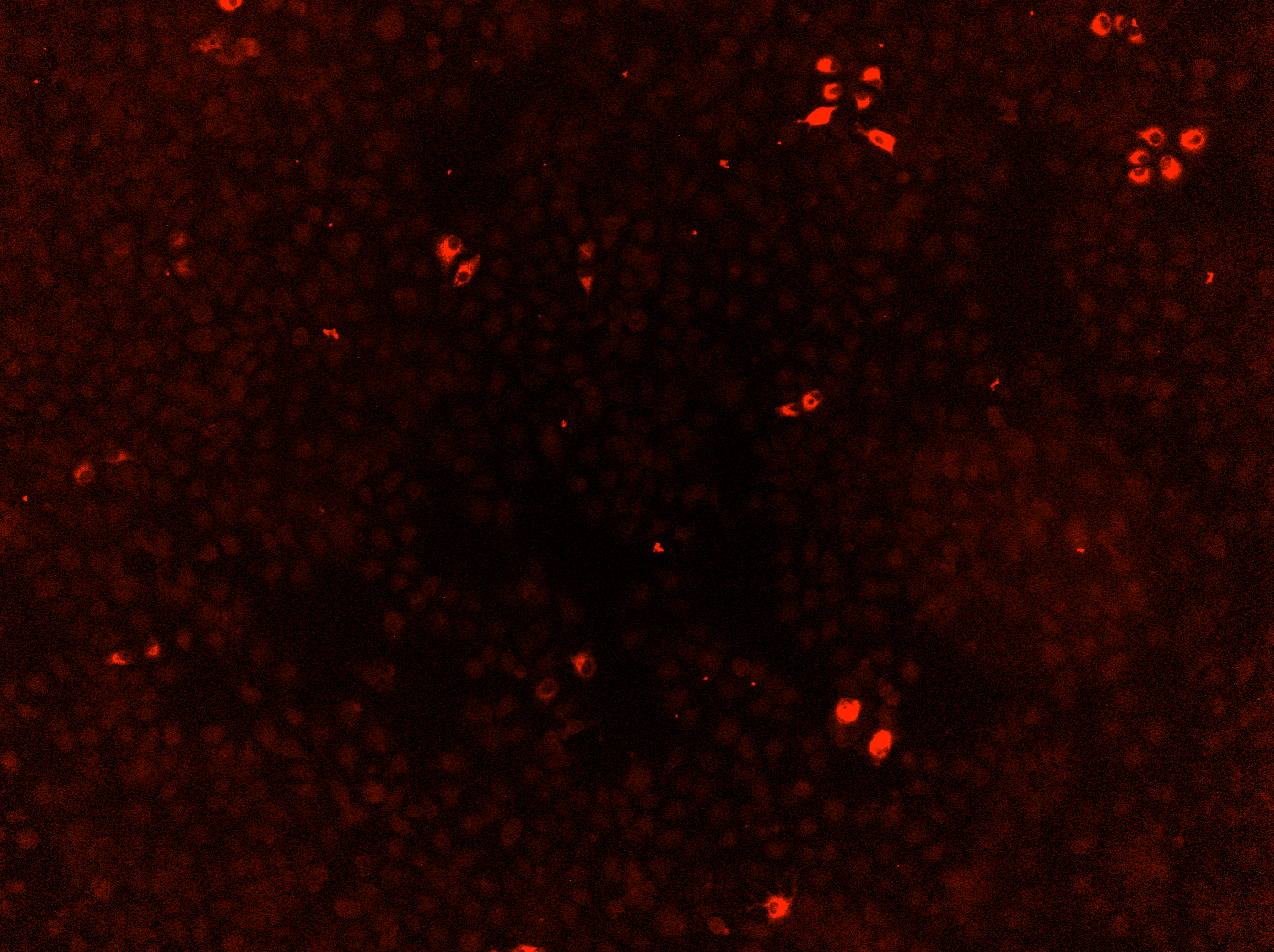

Supplement: Figure 7—figure supplement 1—source data 1. [file elife-52555-fig7-figsupp1-data1.zip › SD-figureS9/Steve G images/Wild type/SteveG_A12_1.tif]

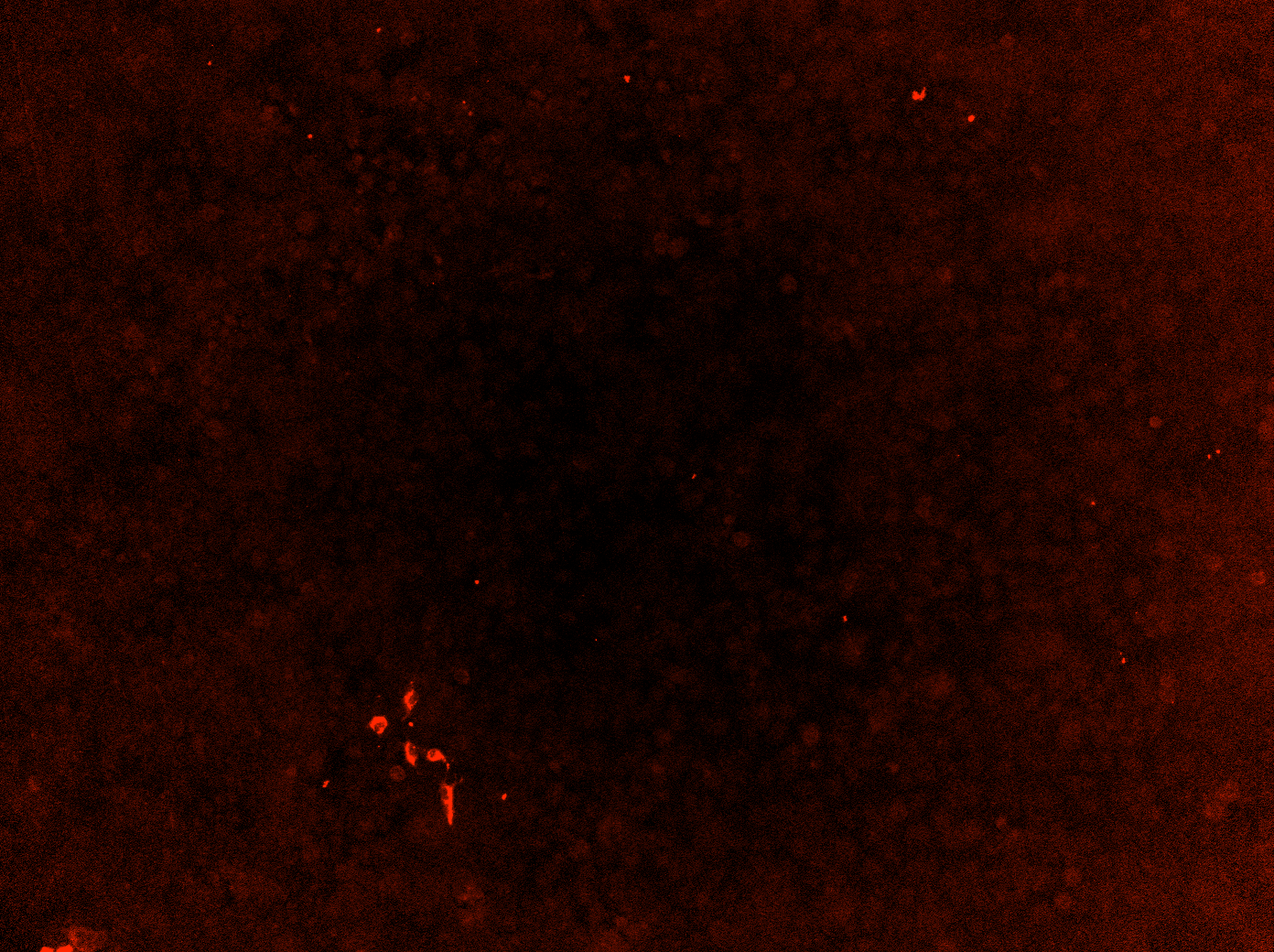

Supplement: Figure 7—figure supplement 1—source data 1. [file elife-52555-fig7-figsupp1-data1.zip › SD-figureS9/Steve G images/Wild type/SteveG_A1_1.tif]

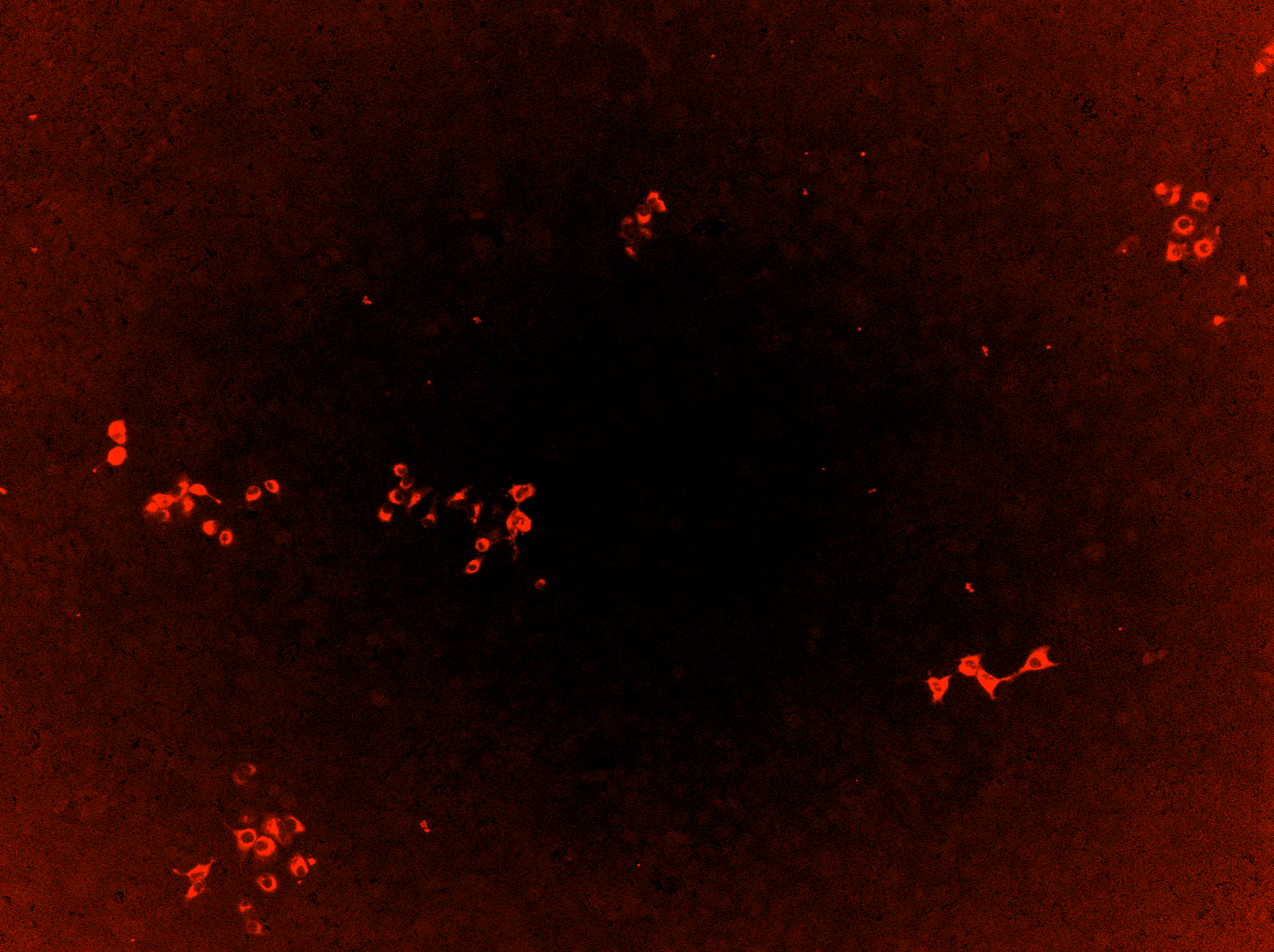

Supplement: Figure 7—figure supplement 1—source data 1. [file elife-52555-fig7-figsupp1-data1.zip › SD-figureS9/Steve G images/Wild type/SteveG_A2_1.tif]

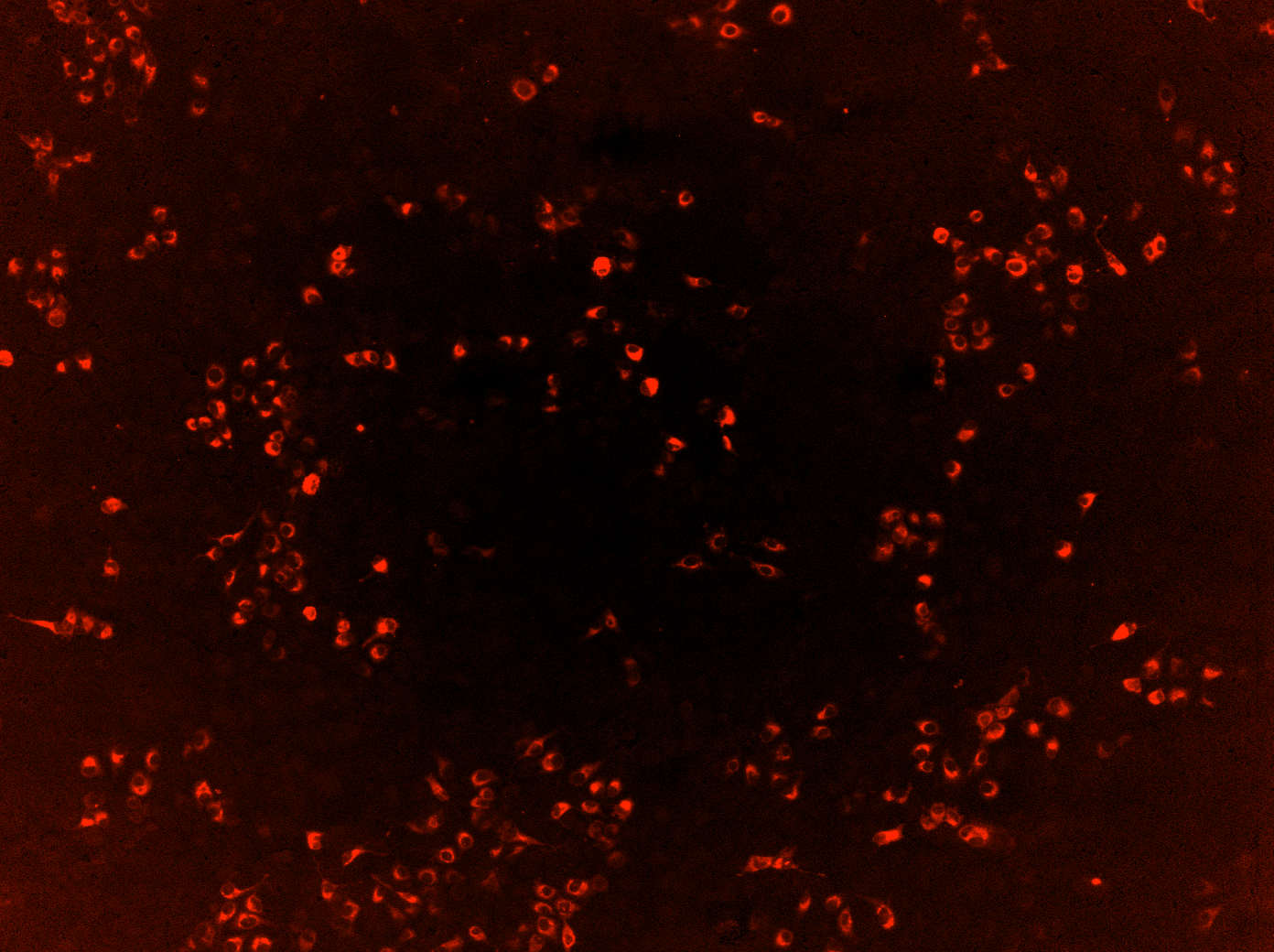

Supplement: Figure 7—figure supplement 1—source data 1. [file elife-52555-fig7-figsupp1-data1.zip › SD-figureS9/Steve G images/Wild type/SteveG_A3_1.tif]

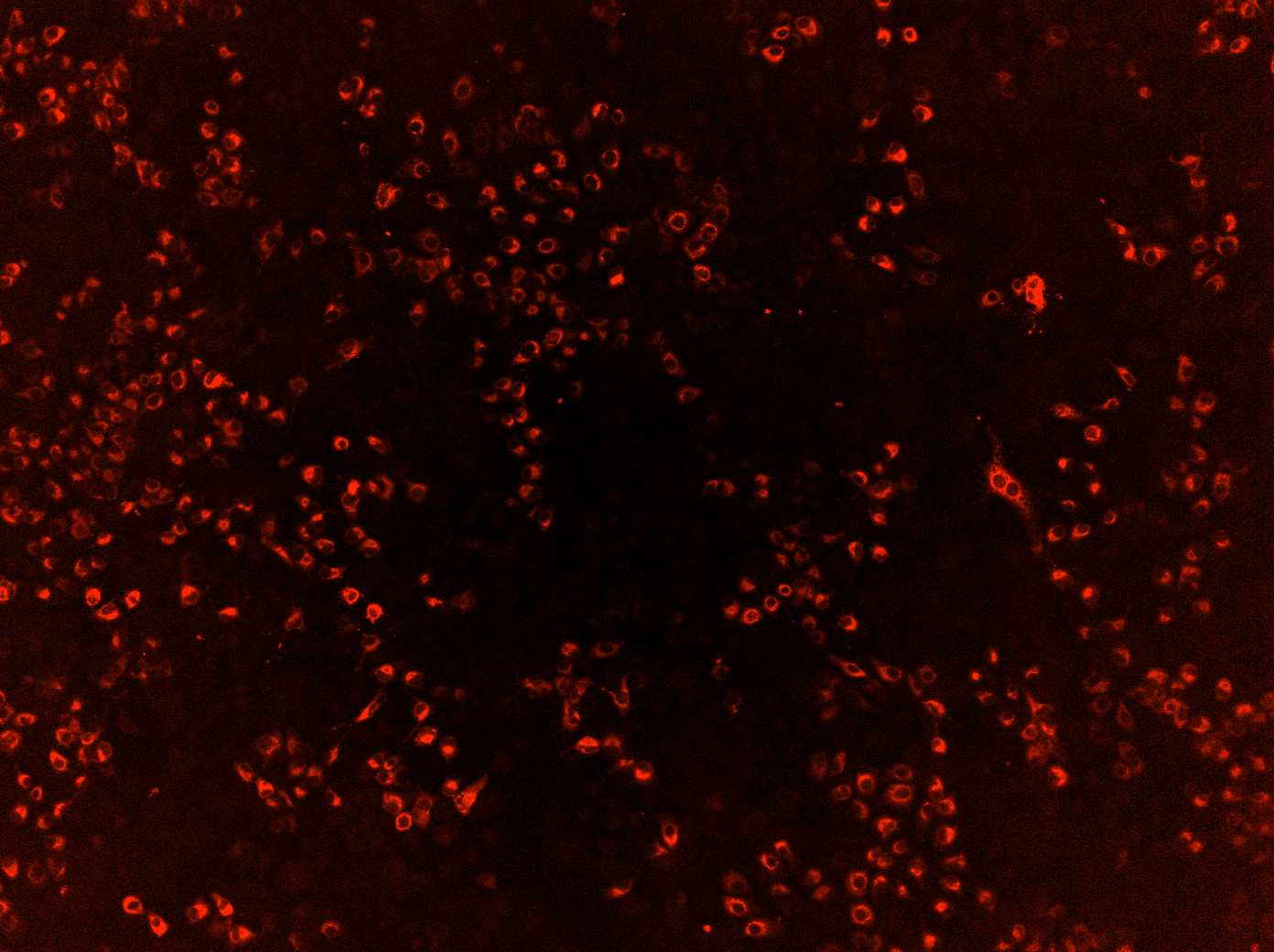

Supplement: Figure 7—figure supplement 1—source data 1. [file elife-52555-fig7-figsupp1-data1.zip › SD-figureS9/Steve G images/Wild type/SteveG_A4_1.tif]

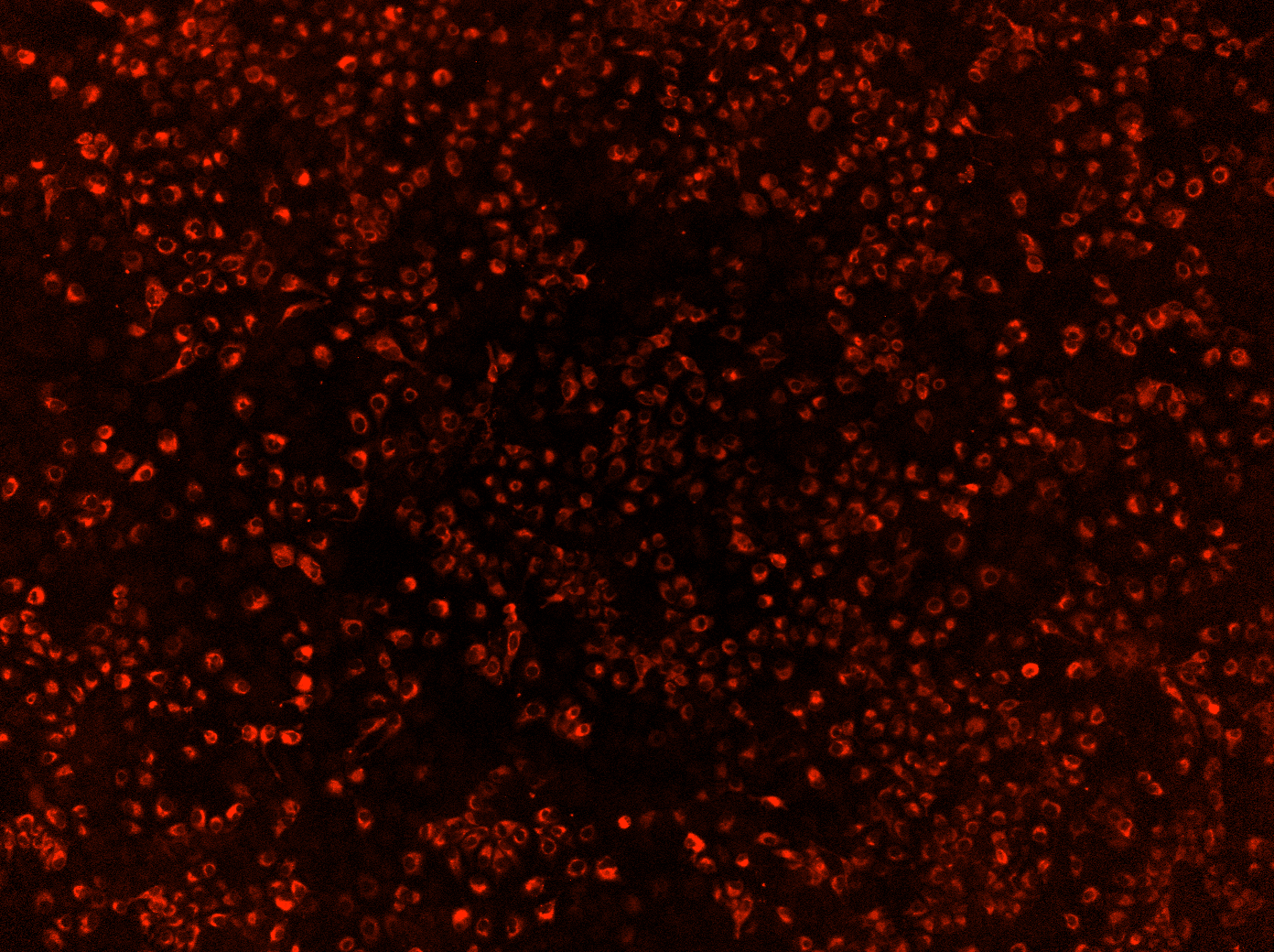

Supplement: Figure 7—figure supplement 1—source data 1. [file elife-52555-fig7-figsupp1-data1.zip › SD-figureS9/Steve G images/Wild type/SteveG_A5_1.tif]

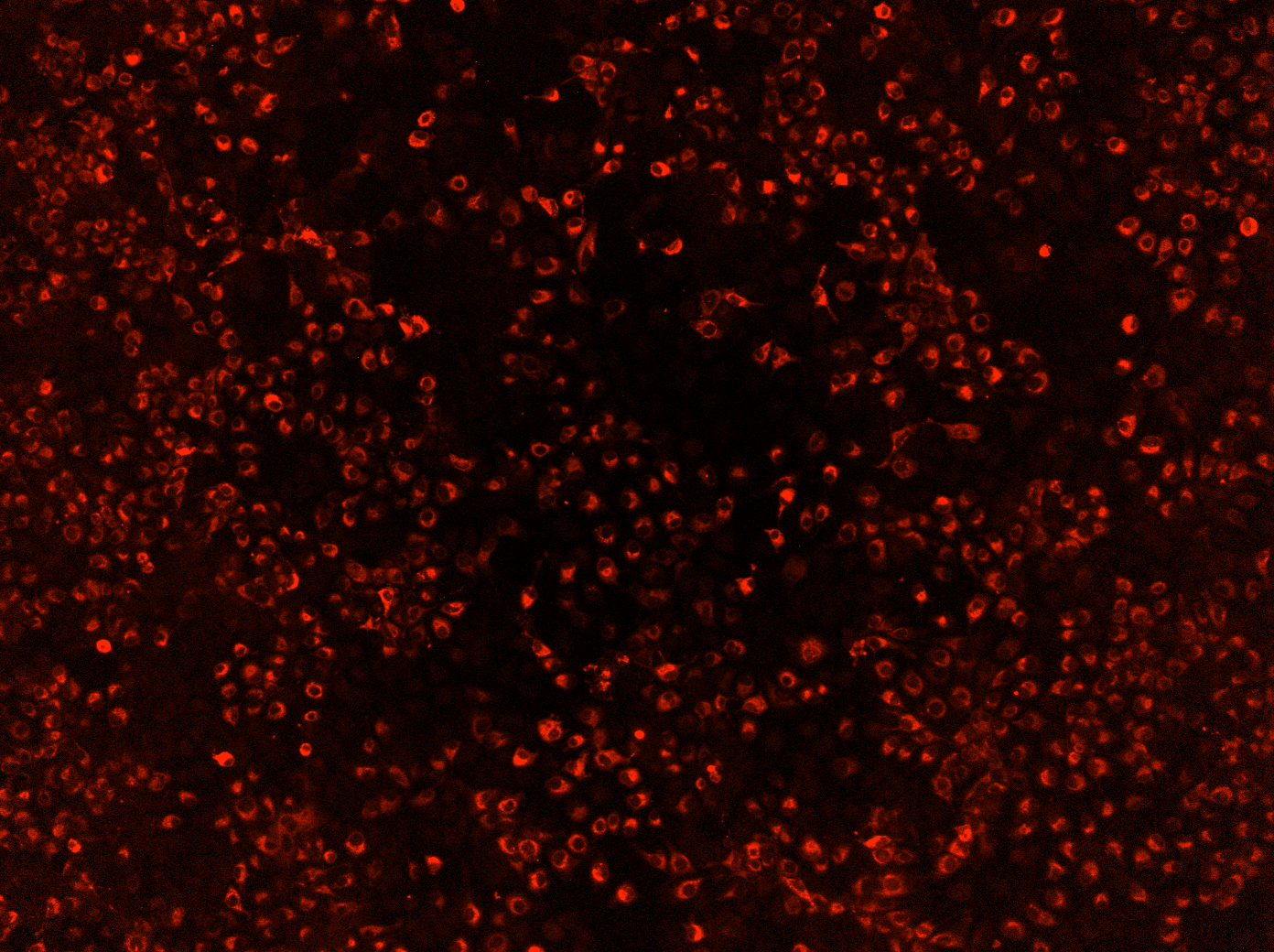

Supplement: Figure 7—figure supplement 1—source data 1. [file elife-52555-fig7-figsupp1-data1.zip › SD-figureS9/Steve G images/Wild type/SteveG_A6_1.tif]

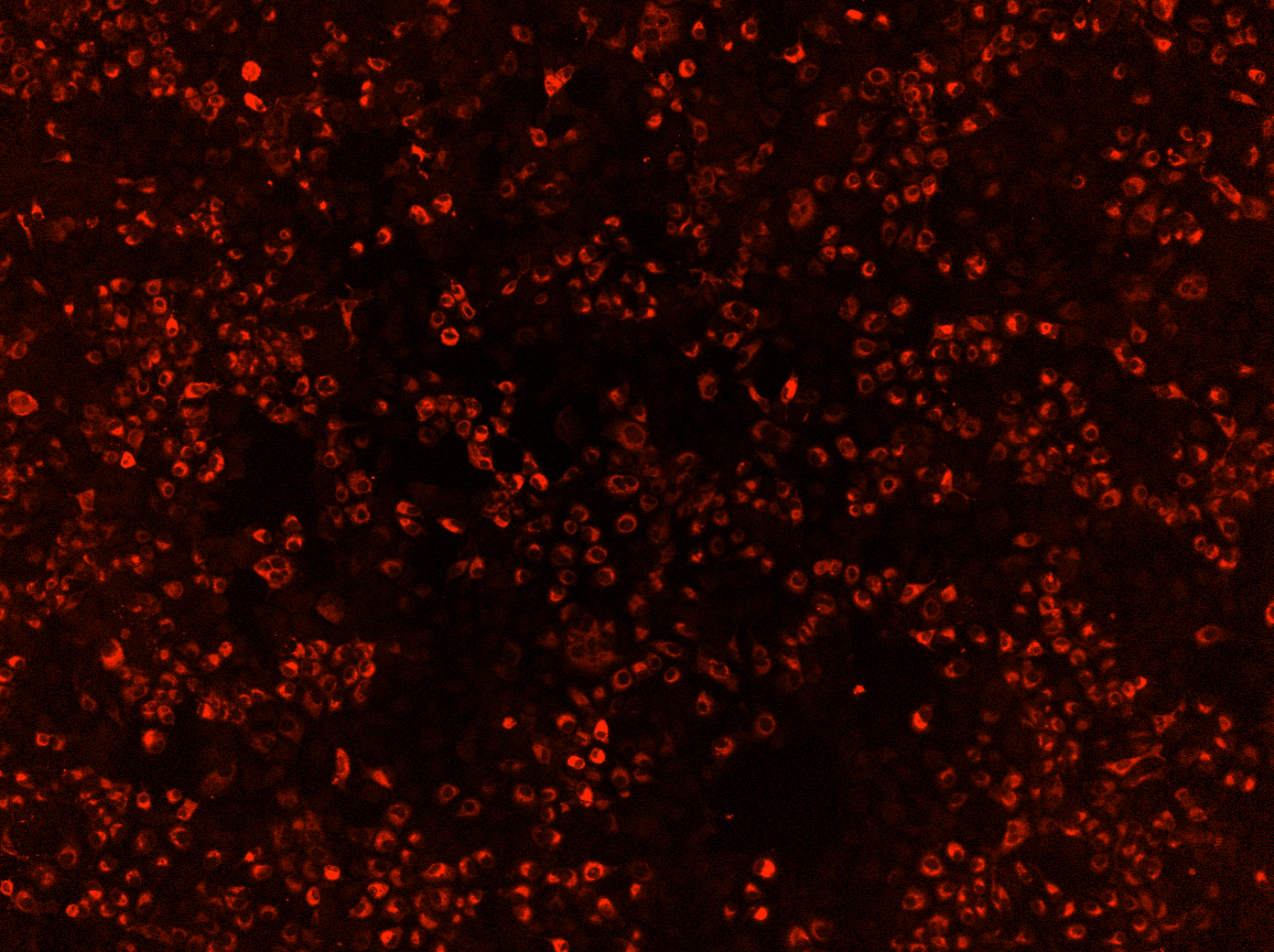

Supplement: Figure 7—figure supplement 1—source data 1. [file elife-52555-fig7-figsupp1-data1.zip › SD-figureS9/Steve G images/Wild type/SteveG_A7_1.tif]

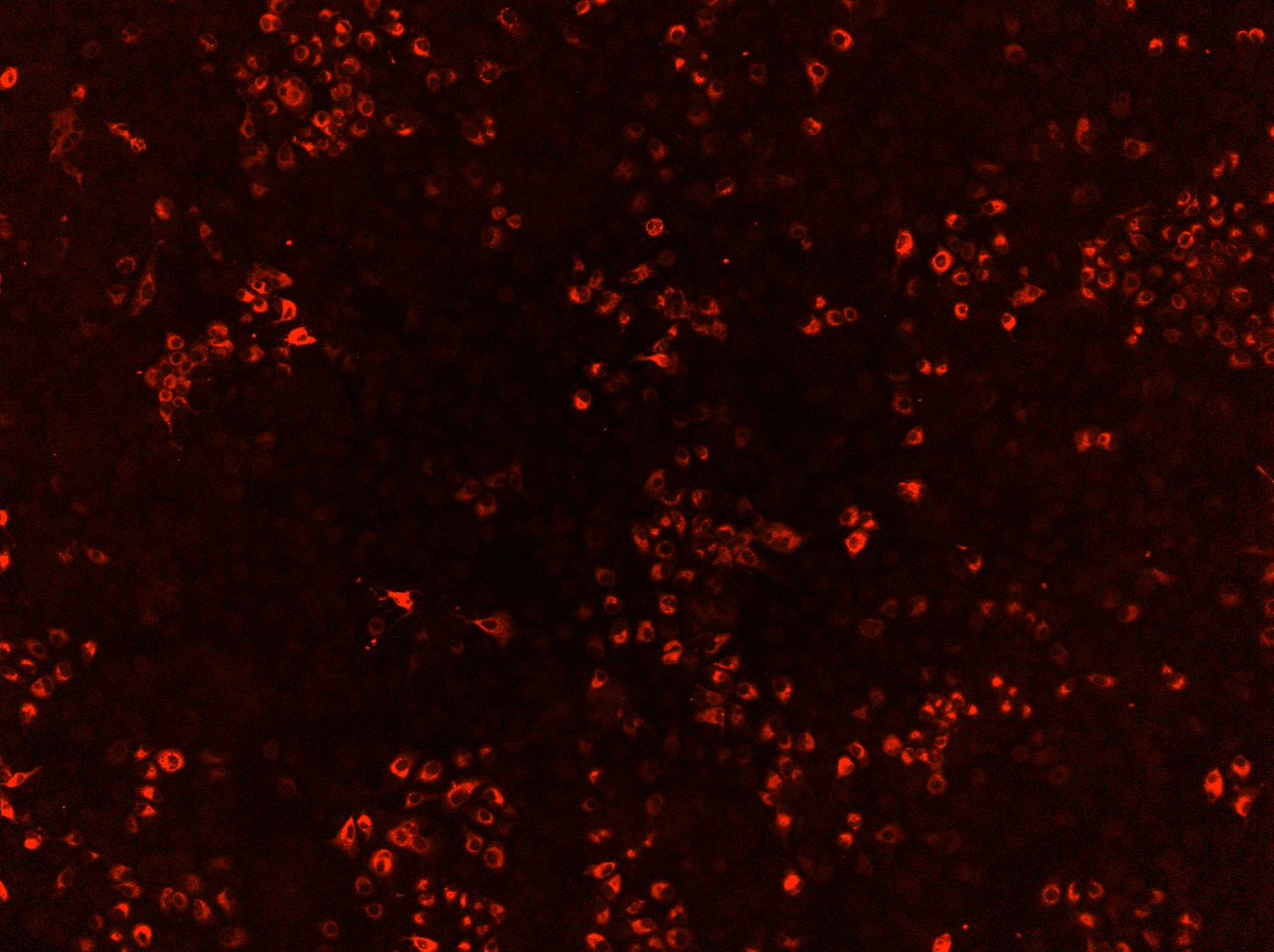

Supplement: Figure 7—figure supplement 1—source data 1. [file elife-52555-fig7-figsupp1-data1.zip › SD-figureS9/Steve G images/Wild type/SteveG_A8_1.tif]

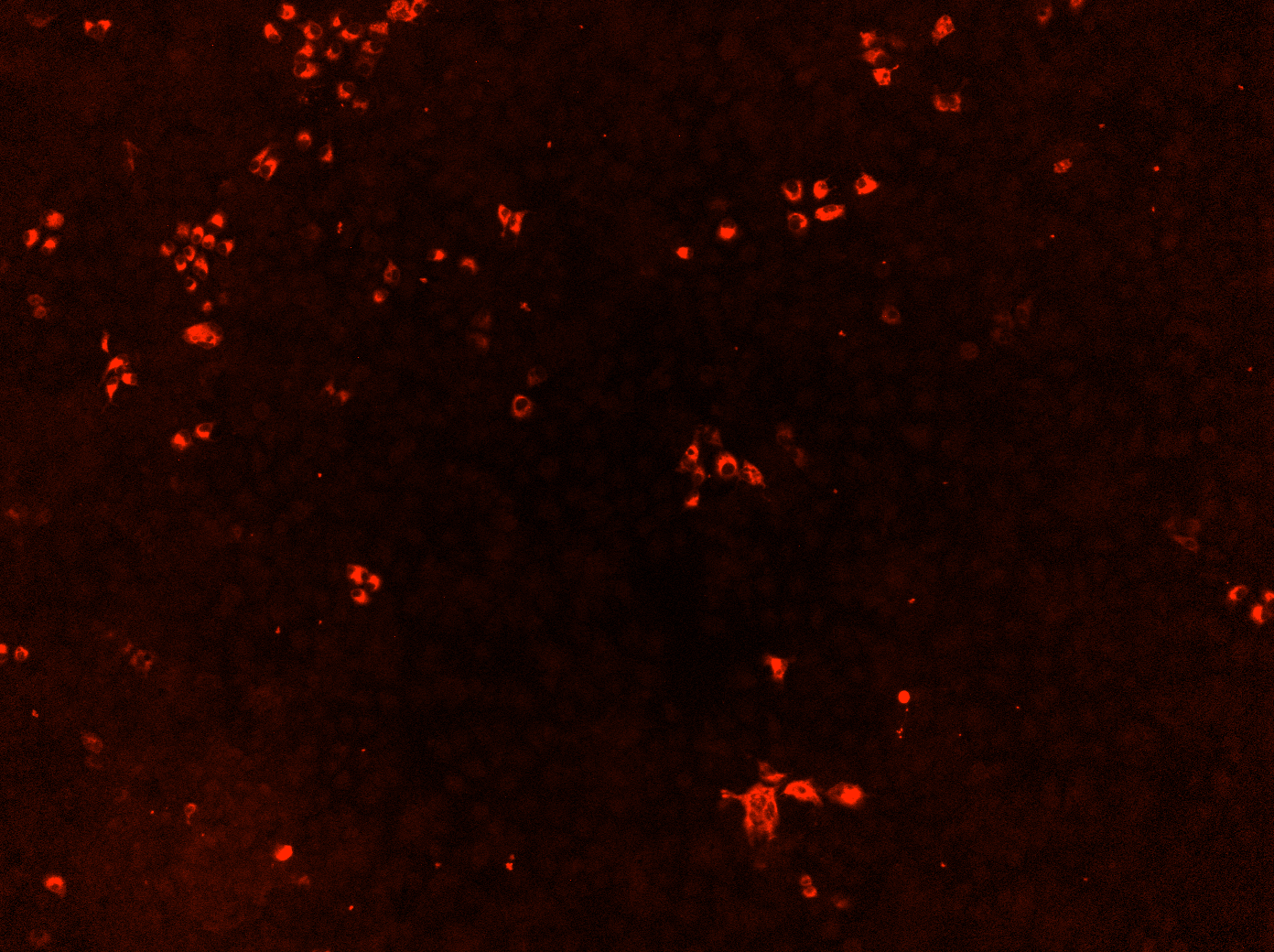

Supplement: Figure 7—figure supplement 1—source data 1. [file elife-52555-fig7-figsupp1-data1.zip › SD-figureS9/Steve G images/Wild type/SteveG_A9_1.tif]
