# Supplementary material for: Rationally derived inhibitors of hepatitis C virus (HCV) p7 channel activity reveal prospect for bimodal antiviral therapy
Source: eLife. 2020 Nov 10;9:e52555. doi: 10.7554/eLife.52555 (PMC7714397; doi:10.7554/eLife.52555)
Supplement: Table 1—source data 1. [file elife-52555-table1-data1.docx]

**Compound syntheses and purification**

***Synthesis of alexafluor-JK3/32 adduct (JK3/32-488):***

***3-[(dimethylamino)methylidene)]-2,3-dihydro-1H-indol-2-one*.**

***3-[(dimethylamino)methylidene]-1-{(4-fluorophenyl)methyl]-2,3-dihydro-1H-indol-2-one.***

***(3Z)-1-[(4-fluorophenyl)methyl]-3-({[4-(prop-2-yn-1-yloxy)phenyl]amino}methylidene)-2,3-dihydro-1H-indol-2-one (1191-146)***

***Alexafluor-JK3/32-triazole (JK3/32-488)*:**

**
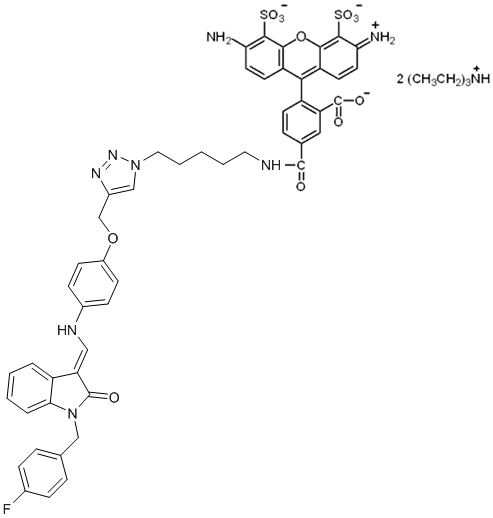
**

***Synthesis of JK3/32:***

***1-benzyl-3-[(dimethylamino)methylidene]-2,3-dihydro-1H-indol-2-one.***

***1-benzyl-3-[(4-methoxyphenyl)aminomethylidene]-2,3-dihydro-1H-indol-2-one (JK3/32)***

***(3Z)-3-[(4-methoxyanilino)methylidene]-1-phenyl-1,3-dihydro-2H-indol-2-one (JK3-42)***

***(3Z)-3-[(4-methoxyanilino)methylidene]-1,3-dihydro-2H-indol-2-one (JK3-38)***

***(Z)-1-benzyl-3-(2-(4-methoxyphenyl)hydrazono)indolin-2-one (21-RS-7)***


 ***(Z)-1-benzyl-3-(2-phenylhydrazono)indolin-2-one (21-RS-8):***

***(Z)-3-(2-(4-methoxyphenyl)hydrazono)-1-phenylindolin-2-one (21-RS-9):***

***1-benzyl-3-(2-(3,4-dimethoxyphenyl)-2-oxoethyl)-3-hydroxyindolin-2-one (21-RS-11 (aka “R21”)):***

***1-benzyl-3-(2-(3,4-dimethoxyphenyl)-2-oxoethylidene)indolin-2-one (21-RS-17)***

***(3Z)-1-benzyl-3-[(2-methoxyanilino)methylidene]-1,3-dihydro-2H-indol-2-one (1191-104)***

***4-{[(Z)-(1-benzyl-2-oxo-1,2-dihydro-3H-indol-3-ylidene)methyl]amino}benzonitrile (1191-112)***

***(3Z)-1-benzyl-6-fluoro-3-[(4-methoxyanilino)methylidene]-1,3-dihydro-2H-indol-2-one (1191-121)***

***4-({(3Z)-5-fluoro-3-[(4-methoxyanilino)methylidene]-2-oxo-2,3-dihydro-1H-indol-1-yl}methyl)benzonitrile (1191-120)***

***(3Z)-5-fluoro-1-[(4-fluorophenyl)methyl]-3-[(4-methoxyanilino)methylidene]-1,3-dihydro-2H-indol-2-one (1191-124)***

***(3Z)-1-[(3,5-dimethyl-1,2-oxazol-4-yl)methyl]-3-[(4-methoxyanilino)methylidene]-1,3-dihydro-2H-indol-2-one (1191-106)***

***(3Z)-3-[(4-methoxyanilino)methylidene]-1-(2-phenylethyl)-1,3-dihydro-2H-indol-2-one (1191-137)***

***(3Z)-1-[(4-fluorophenyl)methyl]-3-[(4-methoxyanilino)methylidene]-1,3-dihydro-2H-indol-2-one (1191-140)***

***(3Z)-3-[(4-fluoroanilino)methylidene]-1-[(4-fluorophenyl)methyl]-1,3-dihydro-2H-indol-2-one (****1191-141)*

***(3Z)-5-fluoro-3-[(anilino)methylidene]-1-[(4-fluorophenyl)methyl]-1,3-dihydro-2H-indol-2-one (****1191-125)*

***4-{[(Z)-(1-(4-fluoro)phenylmethyl-2-oxo-1,2-dihydro-5-fluoro-3H-indol-3-ylidene)methyl]amino}benzonitrile (****1191-126)*
