# Supplementary material for: Rationally derived inhibitors of hepatitis C virus (HCV) p7 channel activity reveal prospect for bimodal antiviral therapy
Source: eLife. 2020 Nov 10;9:e52555. doi: 10.7554/eLife.52555 (PMC7714397; doi:10.7554/eLife.52555)
Supplement: Figure 3—source data 1. [file elife-52555-fig3-data1.zip › SD-figure3/new run 2 files/Doc-01.docx]

The ligand is staying in the pocket in both cases, when simulated either with WT protein or mutant protein.

The carbonyl group of the ligand when simulated with the WT protein is forming a bifurcated hydrogen bond (see the pictures with interaction details) with the side chains of residues Y63 and W66. Within the first 5 ns simulation, the H-bond contact with Y63 is lost but maintains after about 15 ns solely with W66 throughout the rest of the simulation time.

In the mutant protein the phenylalanine causes a reorganization of the binding pocket. The rearrangement looks like crowding of the pocket stabilized by potential π-π stacking interactions between the F38 and Y63. During the rearrangement the carbonyl group of the ligand forms hydrogen bonds solely with the side chain of just Y63 (same as for WT) for about the first 50 ns simulation. In the second half of the simulation time the carbonyl group also engages in hydrogen bonding with W66, suggesting a bifurcated bond system with the side chains of the two amino acids. The second hydrogen bond of the ligand derives from the interaction of the amino group of the ligand with the respective amino acid side chains.
