# Supplementary material for: Rationally derived inhibitors of hepatitis C virus (HCV) p7 channel activity reveal prospect for bimodal antiviral therapy
Source: eLife. 2020 Nov 10;9:e52555. doi: 10.7554/eLife.52555 (PMC7714397; doi:10.7554/eLife.52555)
Supplement: Figure 3—figure supplement 2—source data 1. [file elife-52555-fig3-figsupp2-data1.zip › SD-figureS7/Simulation-result-Rimantadine.pdf]

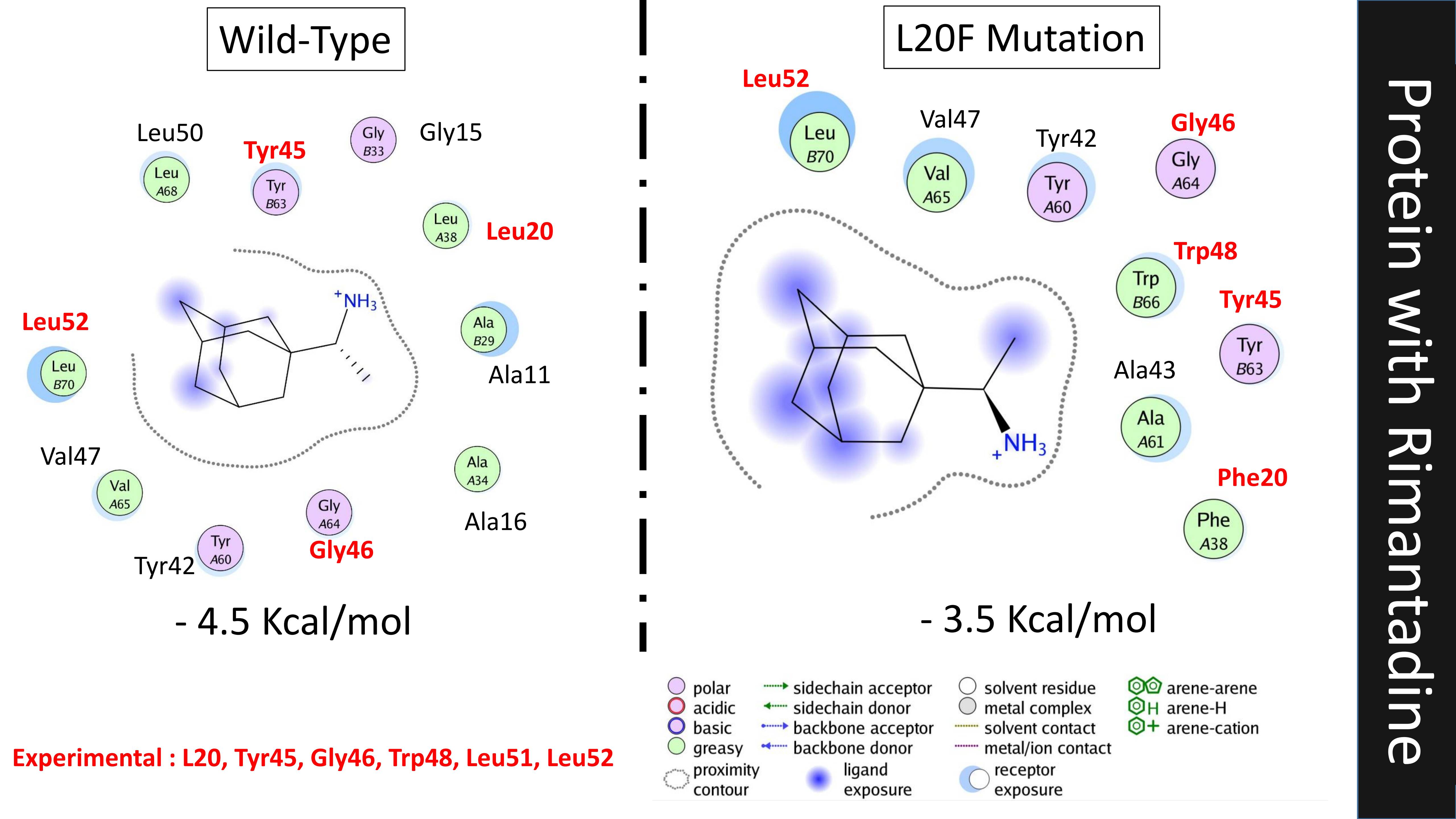
Wild-Type
Leu50
Tyr45
Gly B33
Gly15
Leu A68
Tyr B63
Leu A38
Leu20
+NH3
Ala B29
Ala11
Leu52
Leu B70
Val47
Val A65
Ala A34
Ala16
Gly A64
Gly46
Tyr A60
Tyr42
- 4.5 Kcal/mol
Experimental : L20, Tyr45, Gly46, Trp48, Leu51, Leu52
L20F Mutation
Leu52
Leu B70
Val47
Val A65
Tyr42
Tyr A60
Gly46
Gly A64
Trp48
Trp B66
Tyr45
Tyr B63
Ala43
Ala A61
+NH3
Phe20
Phe A38
- 3.5 Kcal/mol
polar
acidic
basic
greasy
proximity contour
sidechain acceptor
sidechain donor
backbone acceptor
backbone donor
ligand exposure
solvent residue
metal complex
solvent contact
metal/ion contact
receptor exposure
arene-arene
arene-H
arene-cation
Protein with Rimantadine

Simulation Results

All the simulations were carried out for 100ns. A blank simulation was carried out without any ligand docked into it.

Wild type protein

0ns

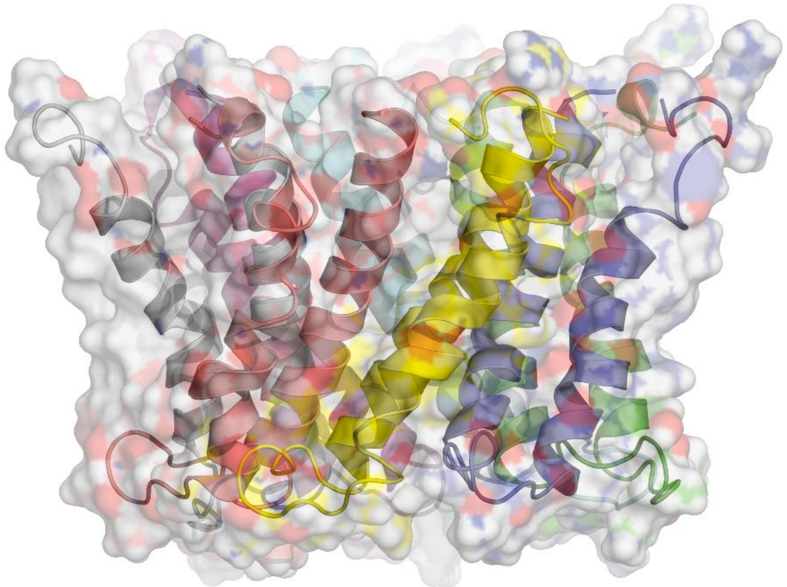

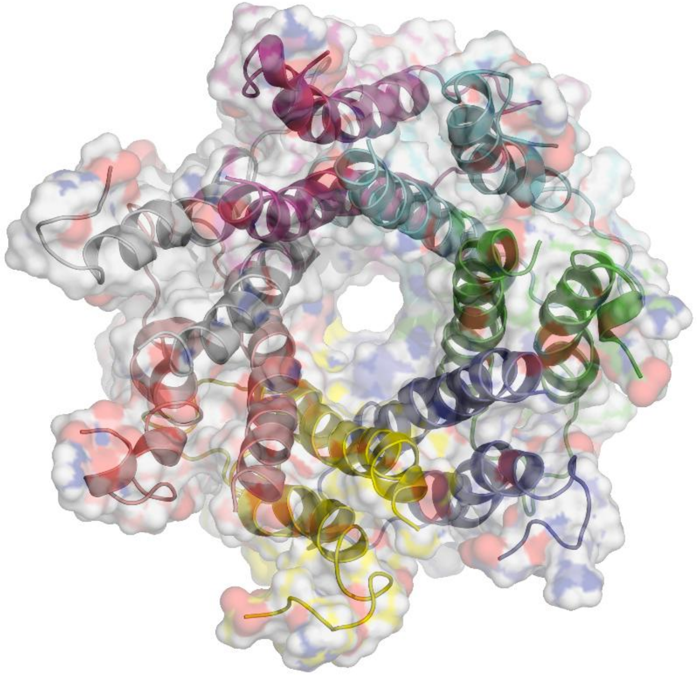

100ns

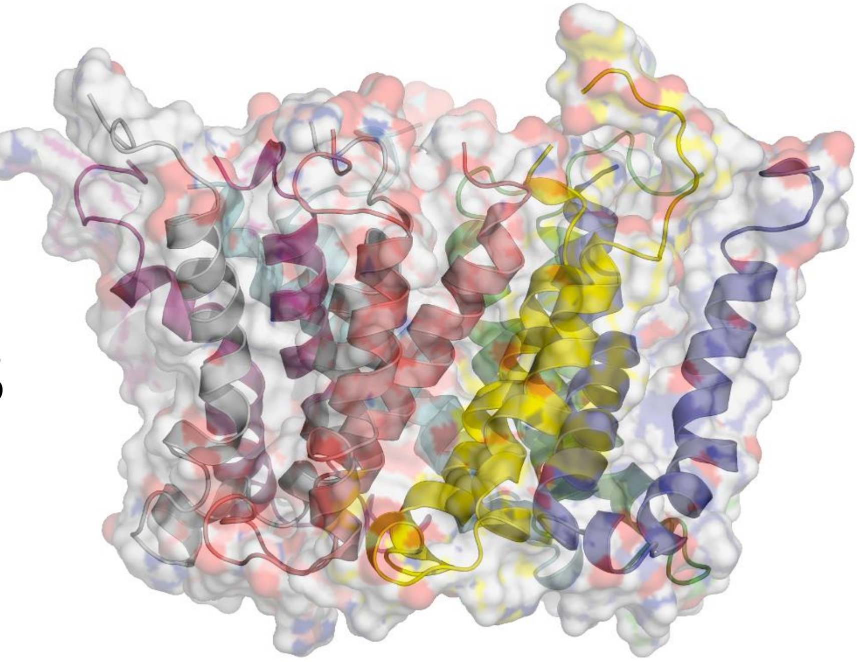

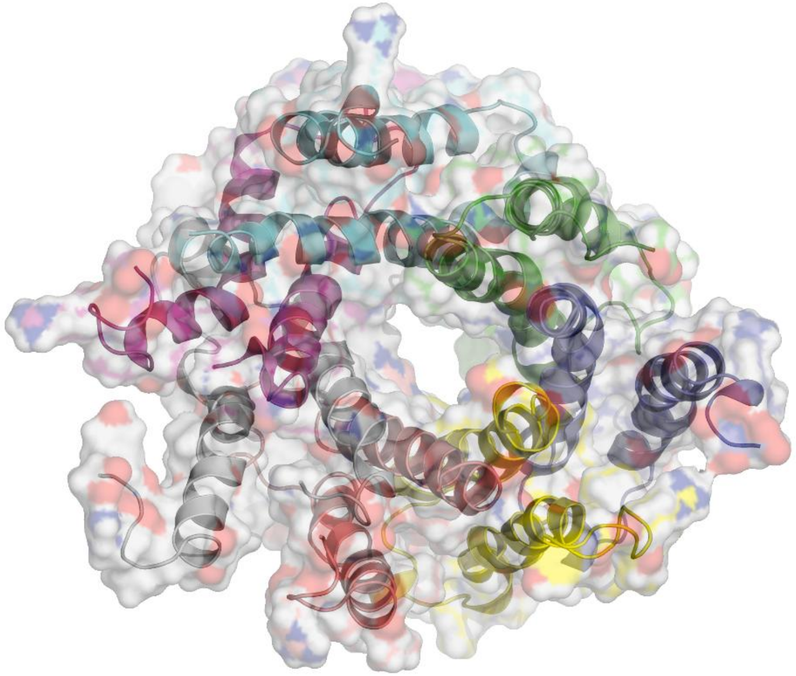

Protein without any ligand

Wild type protein

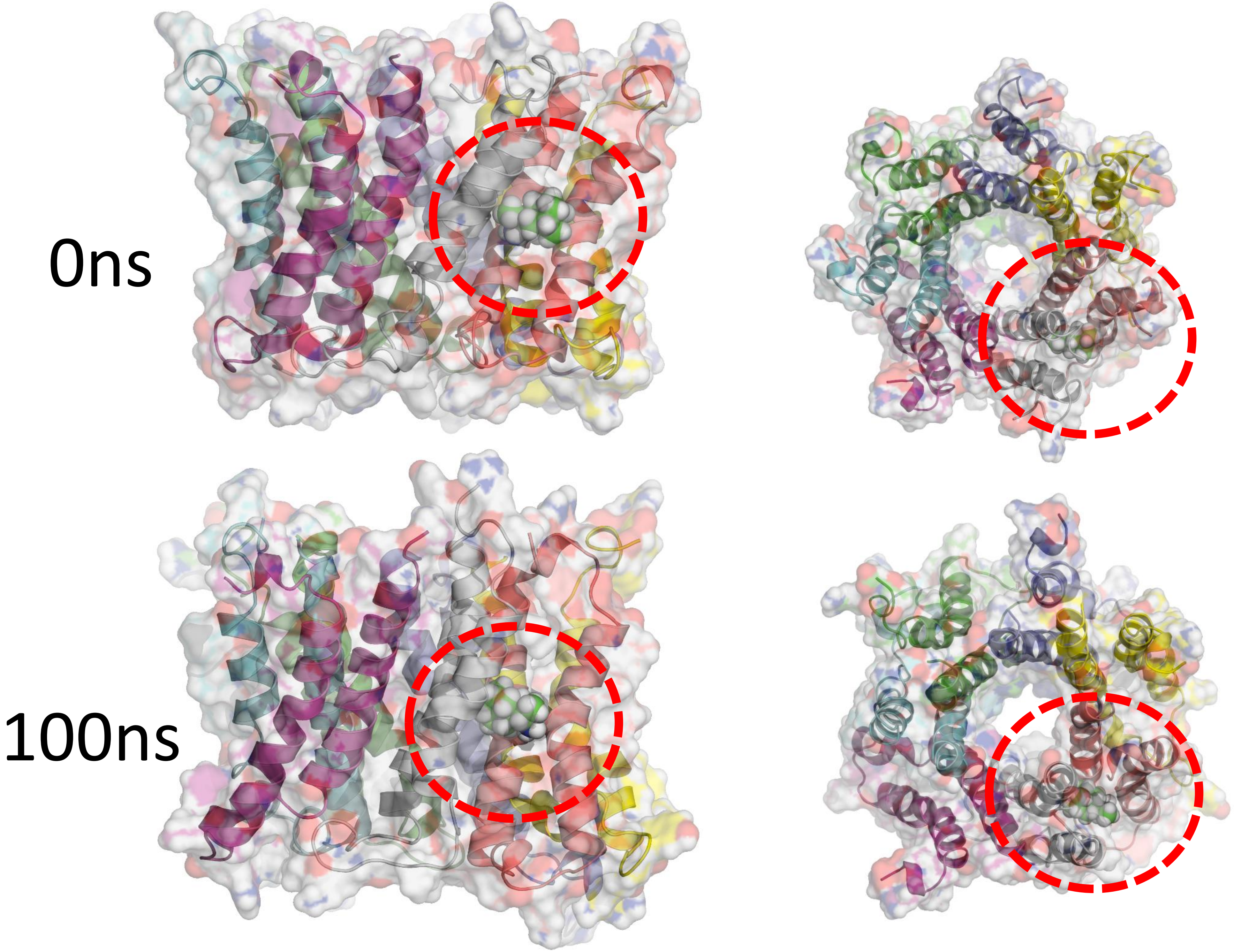

Protein with Rimantadine

Mutant protein

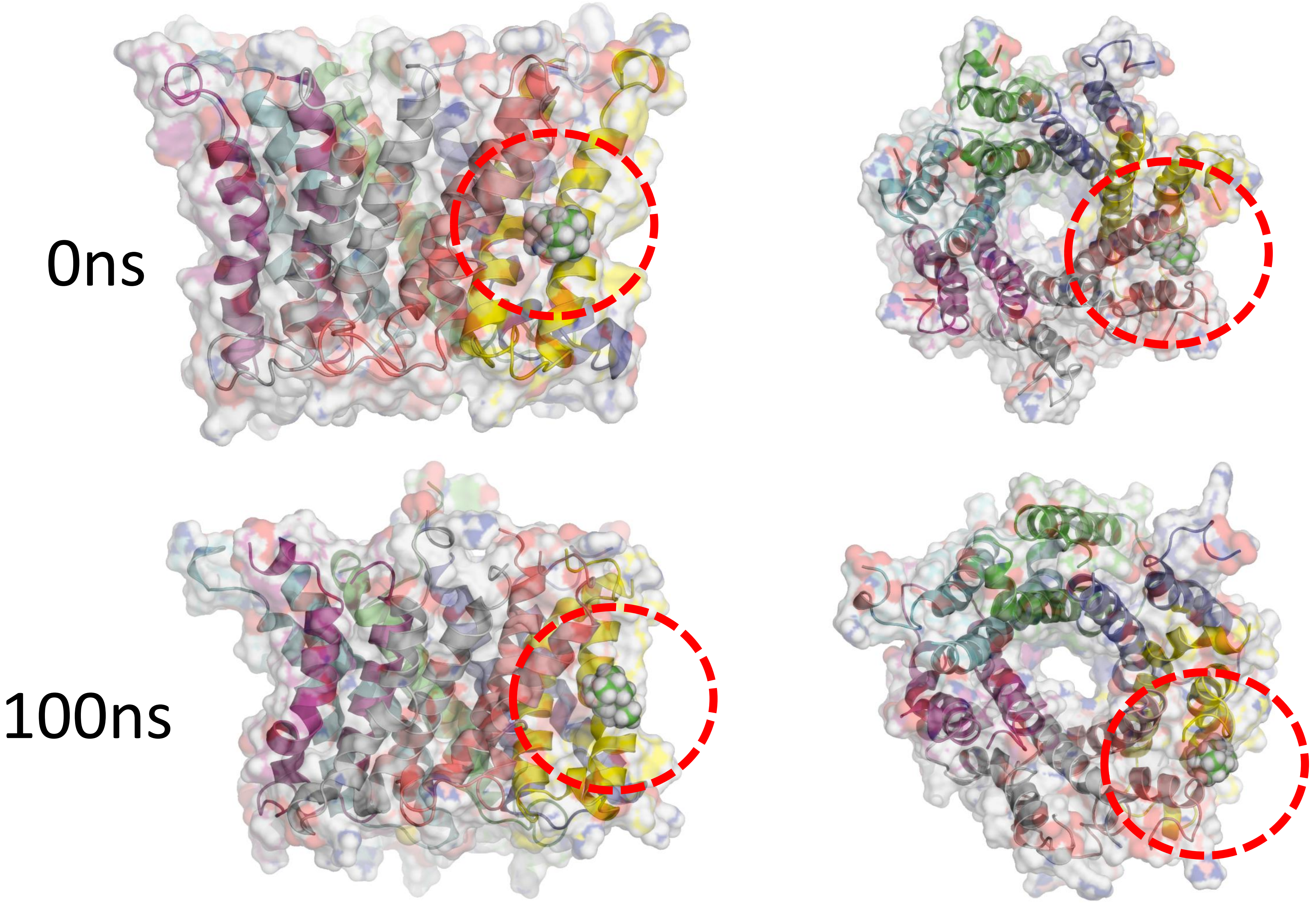

Protein with Rimantadine

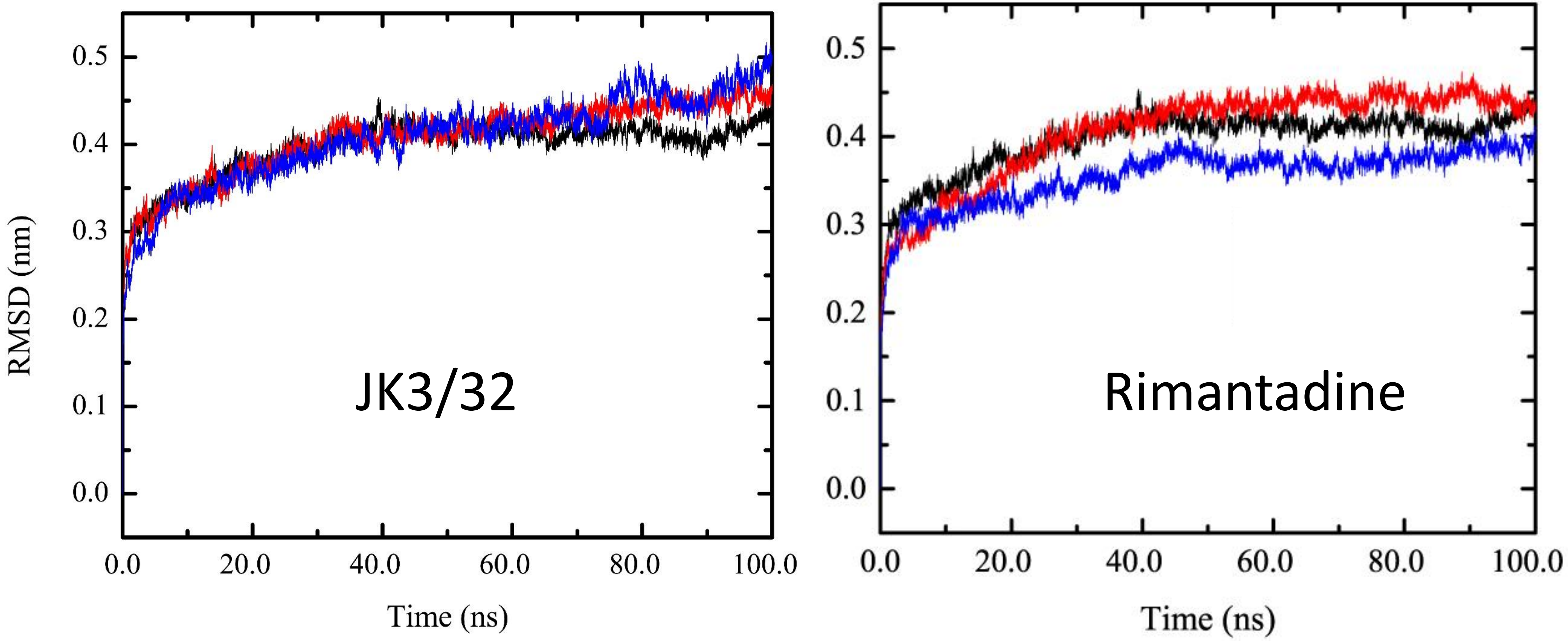

Plot of RMSD values over time for protein without any ligand (**black line**) and with ligand (wild-type: **red line**, L20F mutant: **blue line**)

Protein with Rimantadine

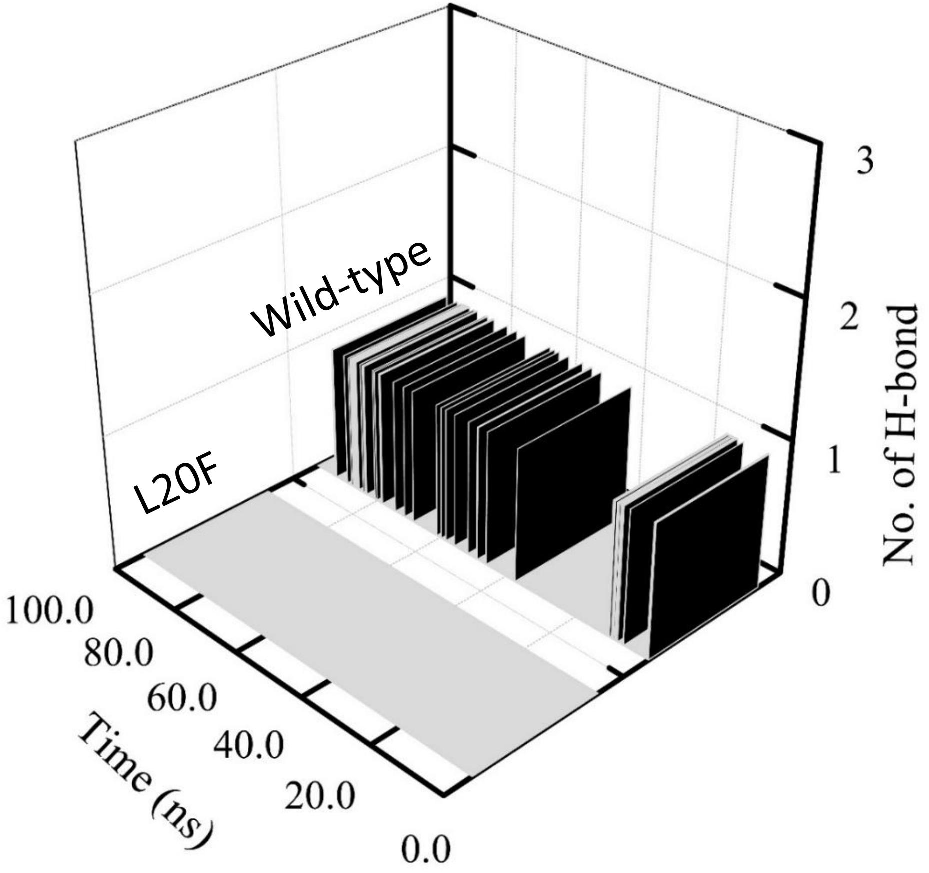

This figure describes the number of H-bonds between the ligand and the side-chains of the protein over the course of simulation

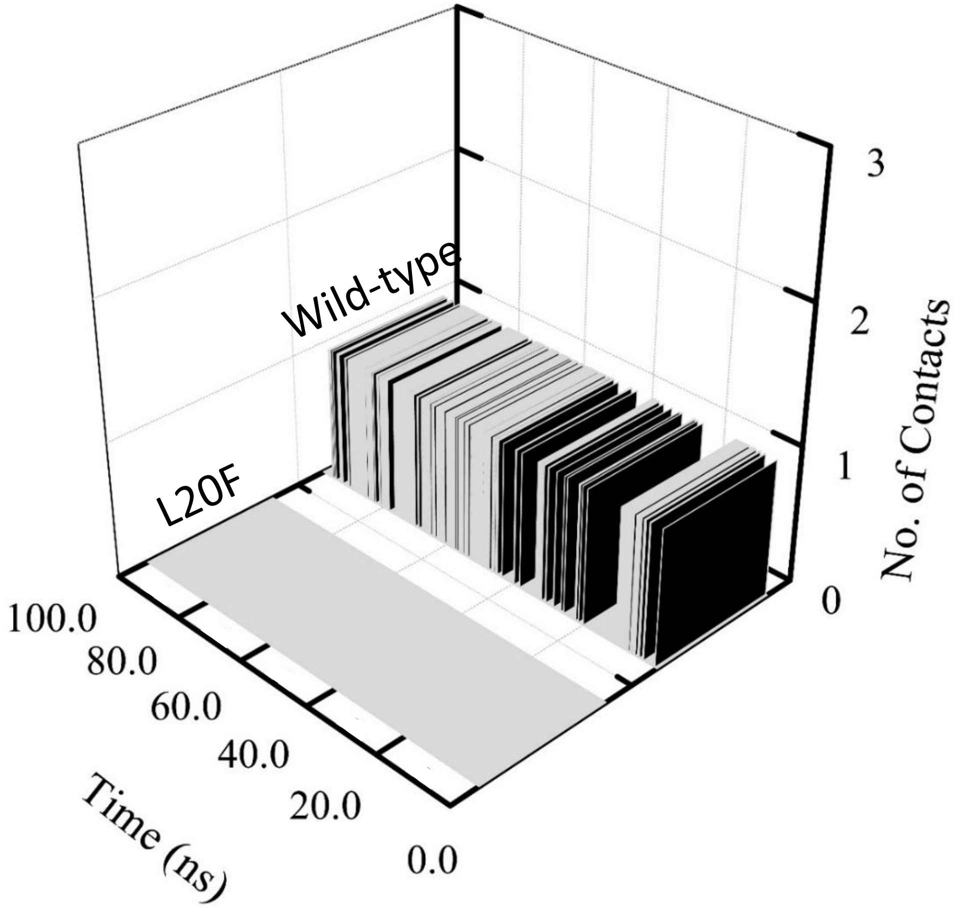

This figure describes the number of contacts (but not bonding) made within a range of 3.5 Å between the ligand and the side-chains of the protein over the course of simulation

From the simulation over 100ns it is evident that only in the **wild type** of the protein, the ligand makes H-bondings and contacts whilst it doesn't in **L20F mutant**. Moreover, in the **wild type**, JK3/32 is in partially continuous H-bonding with the side chains.
